# One-Pot Phosphonylation of Heteroaromatic Lithium Reagents: The Scope and Limitations of Its Use for the Synthesis of Heteroaromatic Phosphonates

**DOI:** 10.3390/molecules28073135

**Published:** 2023-03-31

**Authors:** Ewa Chmielewska, Natalia Miodowska, Błażej Dziuk, Mateusz Psurski, Paweł Kafarski

**Affiliations:** 1 Department of Bioorganic Chemistry, Faculty of Chemistry, Wrocław University of Science and Technology, Wybrzeże Wyspiańskiego 27, 50-370 Wrocław, Poland; 2 Department of Inorganic and Structural Chemistry, Faculty of Chemistry, University of Opole, ul. Oleska 48, 45-052 Opole, Poland; 3 Laboratory of Experimental Anticancer Therapy, Department of Experimental Oncology, Ludwik Hirszfeld Institute of Immunology and Experimental Therapy, Polish Academy of Sciences, Rudolfa Weigla 12, 53-114 Wrocław, Poland; 4 Department of Chemistry, Faculty of Agriculture and Forestry, University of Warmia and Mazury, Plac Łódzki 4, 10-721 Olsztyn, Poland

**Keywords:** aminophosphonates, heteroaromatic, lithiation, phosphonylation, phosphonic acids, organophosphorus chemistry, P-containing drugs

## Abstract

A one-pot lithiation–phosphonylation procedure was elaborated as a method to prepare heteroaromatic phosphonic acids. It relied on the direct lithiation of heteroaromatics followed by phosphonylation with diethyl chlorophosphite and then oxidation with hydrogen peroxide. This protocol provided the desired phosphonates with satisfactory yields. This procedure also had some limitations in its dependence on the accessibility and stability of the lithiated heterocyclic compounds. The same procedure could be applied to phosphonylation of aromatic compounds, which do not undergo direct lithiation and thus require the use of their bromides as substrates. The obtained compounds showed weak antiproliferative activity when tested on three cancer cell lines.

## 1. Introduction

Useful biological and practical applications of heteroaromatic phosphonates, as well as their utility as intermediates in organic synthesis, have stimulated intensive studies directed toward methods of their preparation [1]. As indicated, this is a challenging research endeavor, and quite frequently, the yields of the applied procedures are moderate or even low [2,3,4,5]. Therefore, it is not surprising that the number of papers devoted to the elaboration of methods to prepare these compounds has increased in recent years [6,7,8,9,10,11,12]. The most commonly used method for the introduction of a phosphonate moiety to an sp^2^ hybridized aromatic carbon atom is a palladium-catalyzed reaction [5,6,7,8,9,10,11]. Due to continual improvements in its conditions, catalysts, and procedures, this method provides high yields of products of variable structures. An alternative strategy is to use aryl bromides and the application of copper (I), nickel (II), or manganese (II) catalysts [2,6,13]. Finally, the formation of lithium derivatives or Grignard reagents using aryl halides as substrates may be also employed [2,6,7]. In the past decade, interesting methods of nontypical specific procedures for the synthesis of heteroaromatic phosphonates have been studied and elaborated [12,14,15,16,17,18,19,20,21,22,23,24,25].

It is well known that electron-rich heterocycles easily undergo direct lithiation at Position 2 when alkyllithium is used. The formed salts are important intermediates in the syntheses of many groups of organic compounds [26,27,28,29]. Thus, the objective of this paper is to evaluate the scope and limitations of the direct lithiation of heterocyclic compounds, followed by a one-pot reaction with chlorophosphates or chlorophosphites, applied as a method to synthesize heteroaromatic phosphonic acids. Additionally, the antiproliferative activity of the obtained phosphonic acids was evaluated using three cell lines, RAW 264.7 (used to screen for possible antiosteoporotic and anti-inflammatory activities), PC-3, and MCF-7 (human prostate cancer and breast cancer cell lines, respectively), showing a practical lack of activity of the obtained compounds.

## 2. Results

### 2.1. Chemistry

**Scheme 1:**
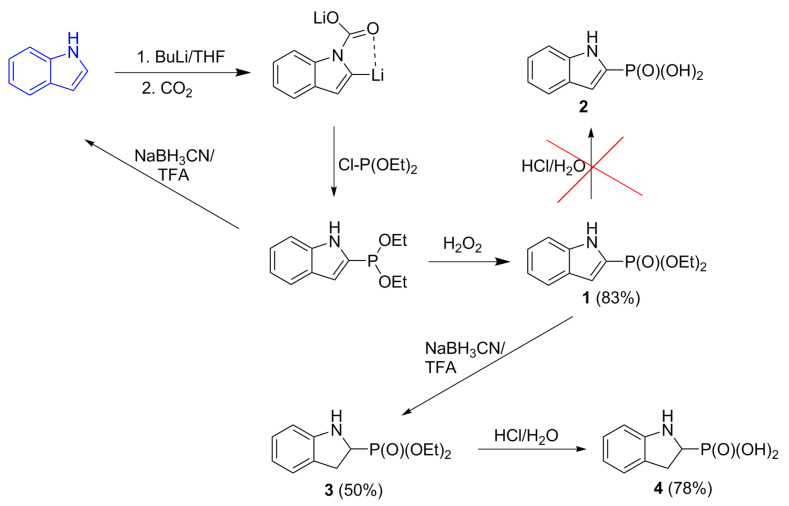
Phosphonylation of indole.

The usefulness of the direct lithiation of indole followed by phosphonylation of the formed lithium salt has been chosen as a model reaction. As shown previously, lithiation of indole with *n*-buthyllithium had to be preceded by the suitable protection of the reactive amino group [28]. The use of *t*-butoxycarbonyl-(*Boc*) protection followed by reaction with trivalent phosphoryl chloride, namely, with 1-chloro-*N*,*N*-diisopropyl-1-methoxyphosphinamine, followed by an aqueous work-up, was described as a method for the synthesis of methyl 2-indolyl phosphinate [30]. Unfortunately, lithiated *Boc*-indole, when reacted with both diethyl chlorophosphate and chlorophosphite, failed to produce the desired product. Thus, the one-pot synthesis of Kartitzky and Akutagawa was adopted [31], which relied on the conversion of indole into 1*H*-indolyl-1-carboxylic acid prior to phosphonylation (Scheme 1). Initially, the utility of diethyl chlorophosphate as an electrophile was planned; however, the literature reports [32] suggested that better results were obtained with the use of diethyl chlorophosphite. Hydrolysis of the resulting diethyl 1*H*-indol-2-ylphosphonate (compound **1a**) in concentrated hydrochloric caused the decomposition of the obtained esters with the simultaneous formation of phosphorous acid (H_3_PO_3_). Moreover, the application of mild conditions for the removal of ester groups from the phosphonate moiety, by conversion of **1** into its di(trimethylsilyl) ester with trimethylchlorosilane, followed by its cleavage with methanol, also failed to deliver the desired acid **2 [33]** and yielded similar products of decomposition. 

The reduction of diethyl 1*H*-indol-2-ylphosphonate **1** provided diethyl indoline-2-phosphonate (compound **3**), which was readily converted into the corresponding acid **4**, albeit with moderate yield. Quite unexpectedly, the hydrolysis of diethyl 1*H*-indol-2-ylphosphinate also failed, and the decomposition of the molecule with production of indole was observed.

**Scheme 2:**
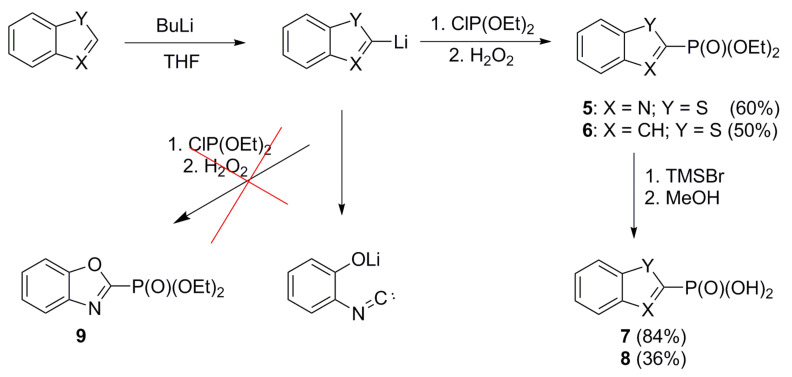
Phosphonylation of benzothiazole, benzothiophene and benzoxazole.

The elaborated procedure was then used for the phosphonylation of benzothiophene and benzothiazole (Scheme 2). There was no need to apply the Kartitzky and Akutagawa procedure in this case. The only disadvantage of this method was the extreme instability of the lithium derivative of benzothiazole. This problem was solved by slight modifications of the synthetic procedure and reversal of the order of addition of reagents; the heterocyclic compound was added to the solution of *n*-butyllithium. Because the resulting phosphonates esters, diethyl benzo[*d*]thiazol-2-ylphosphonate **5** and diethyl benzo[*b*]thiophen-2-ylphosphonate **6,** could be unstable under harsh acidic conditions, we applied transesterification with bromotrimethylsilane for the preparation of the corresponding acids benzo[*d*]thiazol-2-ylphosphonic acid **7** and benzo[*b*]thiophen-2-ylphosphonic acid **8**.

The phosphonylation of the lithiated benzoxazole failed, resulting in a complex mixture of products despite the modifications to the synthetic procedure. This may derive from the possible opening of the pentacyclic ring of this compound upon the action of lithium [34]. According to the published data, the opened product is in equilibrium with lithiated benzoxazole (Scheme 2); however, it did not react with diethyl chlorophosphite in the desired manner, and the compound **9** was not obtained.

**Scheme 3:**
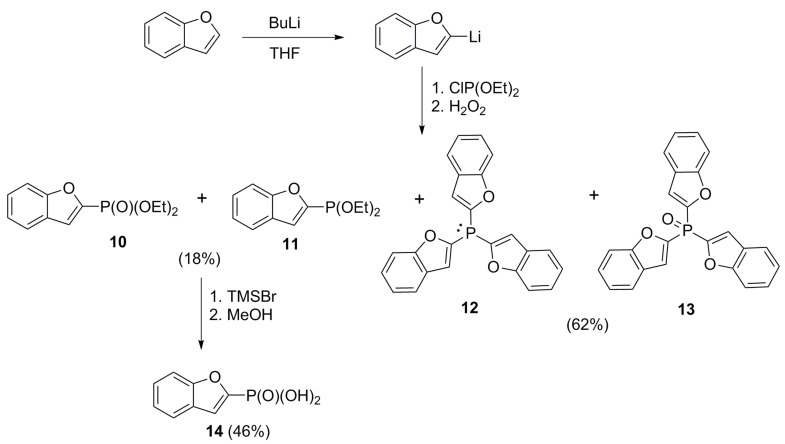
Phosphonylation of benzofurane.

In turn, the reaction of benzofuran yielded the inseparable mixture of phosphonate **10** and phosphinate **11** in moderate yield accompanied with the equimolar mixture of tri-2-benzofuryl phosphine **12** and its oxide **13**. This mixture was the major reaction product (Scheme 3). The mixture of compounds **12** and **13** readily crystallized out over time from the reaction mixture. The pure compound **13** was obtained in a small quantity only in a single case by crystallization from the spent solvent after the crystallization of the mixture of compounds **12** and **13**. The transesterification of the ester groups of the mixture of **10** and **11** with bromotrimethylsilane followed by methanolysis afforded benzofuran-2-ylphosphonic acid **14**.

The crystallographic data are given in Table 1. 

**Figure 1:**
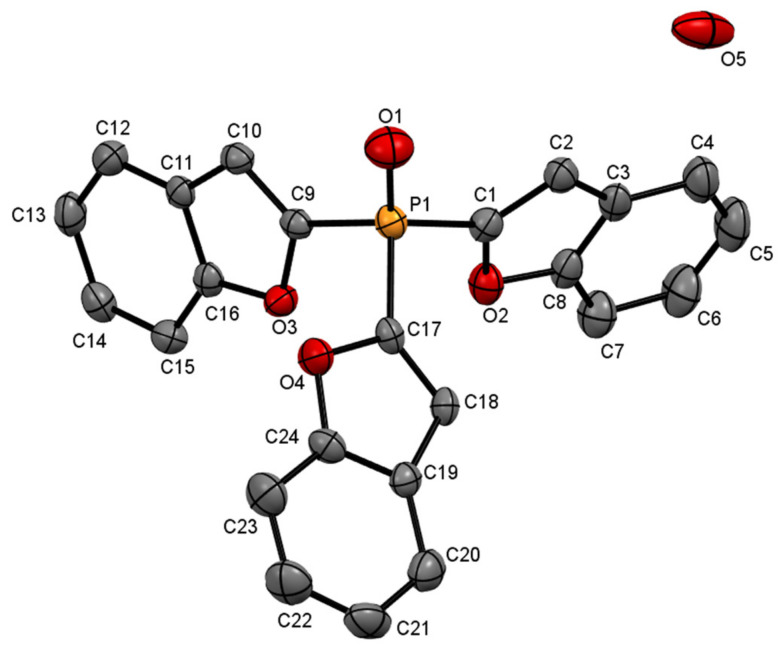
Compound **13** in the asymmetric part of the unit cell. The displacement ellipsoids are drawn at the 50% probability level. Hydrogen atoms are omitted for clarity.

**Figure 2:**
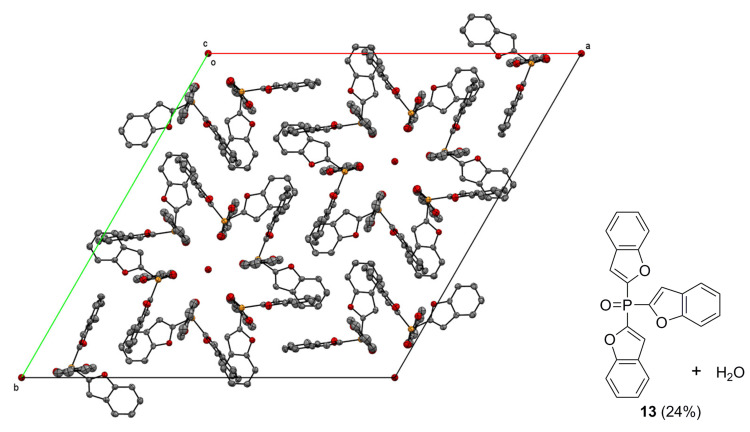
Crystal packing of the complex of tri-2-benzofuryl phosphine oxide **13.** The displacement ellipsoids are drawn at the 50% probability level. Hydrogen atoms are omitted for clarity.

The solid-state structure of the crystals was determined by the X-ray diffraction method. The structure was solved in the trigonal crystal system and the R $\overset{-}{3}$ space group, respectively. In the unit cell, there was tri-2-benzofuryl phosphine oxide (Figure 1) co-crystallized with a water molecule, which occurred on threefold axes. In the crystal structure, there were channels formed by phosphine along the c axis. The partial occupancy oxygen atoms O5 from the water molecules were located in the channels by O-H…O hydrogen bonds, arranging the molecules in crystal packing in quite a rare and specific network (Figure 2). 

**Scheme 4:**
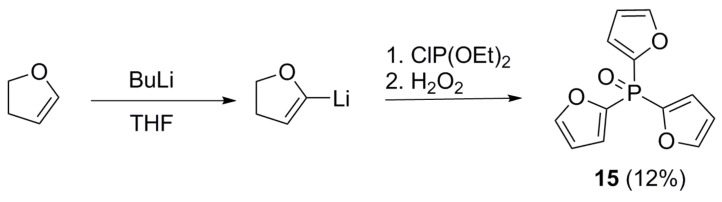
Phosphonylation of furane.

**Figure 3:**
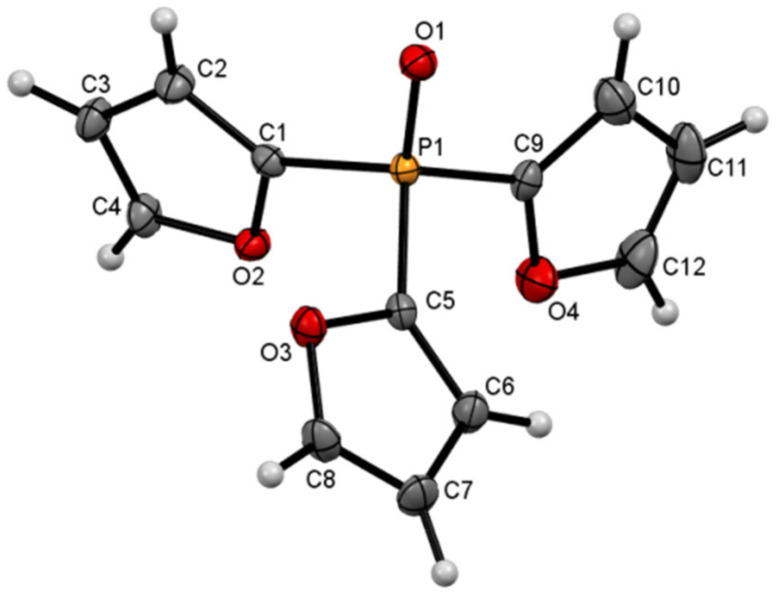
The crystal structure of tri-2-furylphosphinoxide **15**.

The reaction of furan afforded a complex mixture of products from which only tri-2-furylphosphinoxide **15** was isolated in low yield (Scheme 4). Its structure was also confirmed by X-ray analysis (Figure 3). 

The structure of tri-2-furylphosphinoxide **15** was solved in the orthorhombic crystal system and *P*2_1_2_1_2_1_ space group, respectively (Figure 3). This structure was measured at room temperature and compared with the one described earlier by Jenkis and coworkers [35]. In our investigation, for the measurement series, the structure was confirmed at 100 K. In the molecular structure, the bond lengths and angles were within normal ranges [36]. The crystal structure involved intermolecular C-H…O hydrogen bonds. 

It is well known that *N*-methylbenzimidazole and *N*-methylindoles undergo lithiation quite readily at C2 [27]. However, these compounds*,* similar to the unprotected benzimidazole, did not provide the desired phosphonates (reaction of butyllithium with trivalent phosphorus compound), and instead the predominant formations of tributyl phosphine or salts of ethyl butyl phosphinous acid were observed. Moreover, the application of the Kartitzky and Akutagawa procedure failed to provide the desired phosphinates in these cases.

**Scheme 5:**
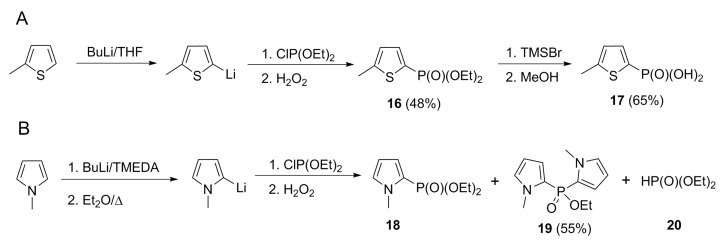
Phosphonylation of 2-methylthiophene (**A**) and N-methylpyrrole (**B**).

To enlarge the scope of the studied reaction, lithiation–phosphonylation of 2-methylthiophene and *N*-methylpyrrole was carried out. As expected, the 2-methylthiophene reacted readily, providing the desired diethyl 2-methyltihophen-5-yl-phosphonate **16**, which upon sequence of transesterification with trimethylbromosilane and methanolysis readily afforded 2-methylthiophene-5-yl-phosphonic acid **17** in good yield (Scheme 5A). Since *N*-methylpyrrole is not susceptible to direct lithiathion [37], addition of a known lithium chelator, tetramethylethylenediamine (TMEDA), and an elevated temperature of the reaction (boiling diethyl ether instead of −70 °C) were required to promote the reaction. In the reaction carried out in this way, an equimolar amount of chlorophosphite was added dropwise to the solution of the lithiated *N*-methylpyrrole. After oxidation with hydrogen peroxide provided the mixture of two esters, diethyl (1*H*-pyrrol-2-yl)phosphonate **18** and ethyl di(1*H*-pyrrol-2-yl)phosphinate **19** (Scheme 5B) were obtained, with compound **19** being the major. Diethyl phosphite **20** was the third product of this reaction. The reversal of the addition of the reagents (lithium reagent added to chlorophosphite solution) also provided the mixture of esters **18** and **19**, still with the predominance of compound **19**.

**Scheme 6:**
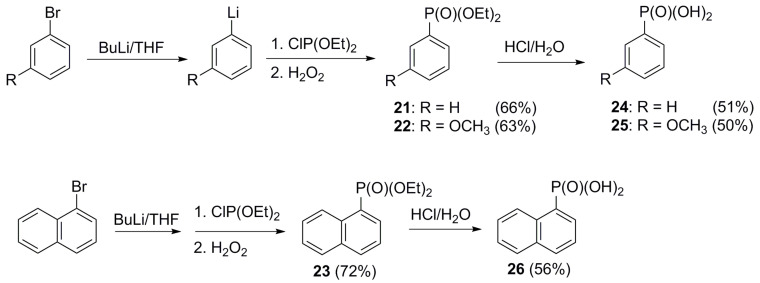
Phosphonylation of bromobenzene, 1-bromo-3-methoxybenzene, and 1-bromonaphthalene.

The utility of the elaborated procedure of direct phosphonylation was additionally proven using bromobenzene, 1-bromo-3-methoxybenzene, and 1-bromonaphthalene as substrates, since the direct lithiation of benzene, anisole, and naphtalene is impossible. As seen from Scheme 6, in this case also, the proposed one-pot procedure had satisfactory results providing the desired diethyl phosphonates (diethyl phenylphosphonate **21**, diethyl 3-methoxylphenylphosphonate **22**, and diethyl naphthalen-1-ylphosphonate **23**), which were converted into the corresponding phosphonic acids (compounds **24**, **25**, and **26**) by hydrolysis with 6N concentrated hydrochloric acid.

### 2.2. In Vitro Antiproliferative Evaluation

The antiproliferative activities of the obtained phosphonic acids were evaluated towards three cell cultures, namely, RAW 264.7, PC-3, and MCF-7. 

The choice of these lines was governed by the fact that the murine macrophage cell line, RAW 264.7, is often used to initially screen the immunomodulating activity of natural compounds [38] and is used as an osteoclast surrogate in preliminary screening for compounds inhibiting osteoclastogenesis [39], thus being promising antiosteoporotic agents. On the other hand, the PC-3 cell is considered as a classic prostate cancer cell line used as a model of androgen-independent prostate cancer [40], while MCF-7 is popular largely due to its exquisite hormone sensitivity through expression of the estrogen receptor, making it an ideal model to study the hormone response [41]. All the studied compounds appeared to be weakly active or lacked activity and could be considered as practically inactive towards these cell lines. The most active compounds are collected in Table 2.

As seen in Table 2, all the studied phosphonic acids more effectively inhibited the proliferation of the RAW 264.7 cells than the effective antiosteoporotic drug, zolendronate, simultaneously being significantly less active than the popular anticancer agent cisplatin. This suggests that they could rather be considered as antiosteoporotic than anti-inflammatory agents. However, in order to determine their potential as antiosteoporotics, more detailed studies are required. 

All the studied compounds exerted quite substantial antiproliferative activity towards PC-3 and MCF-7 cells, being nearly equipotent or even more active than cisplatin. In this respect, compounds **4**, **7**, **24**, and **26** were found to be the most effective. Thus, they might be considered lead substances for further studies. 

## 3. Conclusions

A one-pot lithiation–phosphonylation procedure starting from the lithiation of a heteroaromatic compound with butyllithium, with the further reaction of an intermediate lithium salt with diethylchlorophospite, followed by the oxidation of the product with hydrogen peroxide, appeared to be an interesting alternative for the synthesis of heteroaromatic phosphonates. This simple reaction, however, had significant limitations, which were dependent on the ease of the lithiation procedure, as well as on the stability and reactivity of the lithium intermediate. The elaborated procedure was also successfully used for the preparation of aromatic phosphonates when starting from simple bromoarenes. 

## 4. Materials and Methods

### 4.1. General Information

All solvents and reagents, purchased from commercial suppliers were of analytical grade and were used without further purification. Unless otherwise specified, the solvents were removed with a rotary evaporator. The ^1^H-, ^31^P-, and ^13^C-NMR spectroscopic experiments were performed on a Bruker Avance II Ultrashield Plus (Bruker, Rheinstetten, Germany) operating at 600.58 MHz (^1^H), 243.12 MHz (^31^P{^1^H}), and 151.016 MHz (^13^C), a Bruker Avance III 500 MHz (Bruker, Rheinstetten, Germany) operating at 500.14 MHz (^1^H), 202.46 MHz (^31^P{^1^H}), and 125.77 MHz (^13^C), and a JEOL JNM-ECZ 400S Research FT NMR Spectrometer (JEOL Ltd., Tokyo, Japan) operating at 399.78 MHz (^1^H), 161.83 MHz (^31^P{^1^H}), and 100.53 (^13^C). Measurements were made in CDCl_3_, D_2_O, and solutions of D_2_O + NaOD at 300 K, and all solvents were supplied by Merck Life Science (Darmstadt, Germany). The chemical shifts are reported in ppm relative to TMS and 85% H_3_PO_4_, used as external standards, and the coupling constants are reported in Hz. The melting points were determined on an SRS Melting Point Apparatus OptiMelt MPA 100 (Stanford Research Systems, Sunnyvale, CA, USA) and are reported uncorrected. The purity of all test compounds was higher than 95% by ^1^H NMR and LC-MS. The mass spectra were recorded at the Faculty of Chemistry, Wroclaw University of Science and Technology, using a Waters LCT Premier XE mass spectrometer (method of electrospray ionization, ESI) (Waters, Milford, MA, USA). 

### 4.2. Crystallography

The relevant crystallographic data for the molecules and the full geometrical information are summarized in Table 2 and Supplementary Materials Table S2. The crystals were mounted on a CCD Xcalibur diffractometer (Rigaku Oxford Diffraction, Sevenoaks, Kent, UK), equipped with a CCD detector and a graphite monochromator (Rigaku Oxford Diffraction), with MoKα radiation, λ = 0.71073 Å at 100.0 (1) K. The reciprocal space was explored by ω scans with detector positions at 60 mm distance from the crystal. The diffraction data processing of the studied compounds (Lorentz and polarization corrections were applied) was performed using the CrysAlis CCD software package version 1.171.37.33c; Oxford Diffraction Ltd: Abingdon, Oxfordshire, UK, 2005 [42]. Both crystal structures were solved by direct methods using the SHELXS-2013/1 and SHELXL-2014/7 programs [43,44]. All non-hydrogen atoms were located from difference Fourier synthesis and refined by the least squares method in the full-matrix anisotropic approximation using SHELXL14 software [42,43]. In both structures, the H atoms were located from difference Fourier synthesis. The structure drawings were prepared using the Mercury 2022.3.0 program [45].

The crystallographic data for **13** and **15** have been deposited at the Cambridge Crystallographic Data Centre as supplementary publication nos. CCDC 1904705 for 13 and CCDC 1960286 for 15. These data can be obtained free of charge via http://www.ccdc.cam.ac.uk/conts/retrieving.html (accessed on 5 March 2023) or from the Cambridge Crystallographic Data Centre, 12 Union Road, Cambridge CB2 1EZ, UK; fax: 144-1223-336-033; email: deposit@ccdc.cam.ac.uk.

### 4.3. General Procedure for the Synthesis

#### 4.3.1. Preparation of Diethyl 1*H*-indol-2-ylphosphonate (**1**)

First, 10 mmol of indole (1.17 g) was dissolved in 20 mL dry THF. The solution was cooled to −70 °C while a slow stream of N_2_ was passed through. Subsequently, 10.5 mmol of BuLi (6.6 mL, 1.6 M in hexane) was added to the solution resulting in the appearance of the suspension of the lithium salt. The mixture was left for 30 min at −70 °C. Then, dry CO_2_ was bubbled through the mixture until the solution became completely clear (ca. 10 min), and it was left for 10 min at −70 °C. In the next step, the solvent and excess of CO_2_ were evaporated under reduced pressure at the lowest possible temperature. The resulting solid residue was dissolved in 20 mL of dry THF and cooled to −70 °C under a nitrogen atmosphere. Then, 10.5 mmol of BuLi (6.6 mL, 1.6 M in hexane) was added, and the precipitation of the yellow intermediate was observed. The temperature was maintained at −70 °C for 1 h. Afterwards, 10 mmol (1.56 g, 1.42 mL) of ClP(OEt)_2_ dissolved in 5 mL dry THF was added dropwise, and the resulting mixture was allowed to warm to −40 °C. Subsequently 3 mL of a 30% solution of H_2_O_2_ was added dropwise, and the solution was left to reach room temperature. The resulting mixture was poured out into 30 mL of saturated solution of NH_4_Cl, acidified with sulfuric acid, and slightly warmed. The product was extracted with diethyl ether and purified by column chromatography (silica gel/ethyl acetate). Compound **1** was obtained as a cream-colored solid (2.10 g, 83%); m.p. 66–67 °C (lit.[46] m.p. 81–82 °C); ^31^P-NMR (243.12 MHz, CDCl_3_,): δ = 10.54 ppm; ^1^H-NMR (600.58 MHz, CDCl_3_): δ = 1.36 (t, 6H, *J =* 7.1 Hz, POCH_2_C*H_3_*), 4.12–4.25 (m, 4H, POC*H_2_*CH_3_), 7.09 (s, 1H, C*H* = CP), 7.19 (t, 1H, *J =* 7.4 Hz, Ar*H*), 7.33 (t, 1H, *J =* 8.3 Hz, Ar*H*), 7.51 (d, 1H, *J =* 7.9 Hz, Ar*H*), 9.67 (s, 1H, N*H*) ppm; ^13^C-NMR (151.02 MHz, CDCl_3_): δ = 16.14 (d, *J* = 7.55 Hz, POCH_2_*C*H_3_), 62.98 (d, *J =* 4.53 Hz, PO*C*H_2_), 112.15 (d, *J =* 9.06 Hz, P-C=***C***), 112.26 (d, *J =* 9.06 Hz), 120.48, 121.75, 123.01 (d, *J =* 220.49 Hz, P-***C***=C), 124.60, 127.23 (d, *J =* 15.10 Hz, P-C=C-***C***), 138.43 (d, *J =* 12.08 Hz, P-C=C-N-***C***) ppm; HRMS (ESI + TOF) *m*/*z*: [M + H]^+^. Calcd for C_12_H_16_NO_3_P: 254.0946; found: 254.0954.

#### 4.3.2. Preparation of 1*H*-indolin-2-ylphosphonic Acid (**4**)

To 1 mmol (0.25 g) of ester **1** dissolved in TFA (10 mL) and cooled in an ice bath, 6.3 mmol (0.40 g) of NaBH_3_CN was added carefully over a period of 30 min, and the mixture was left stirring for 24 h at room temperature. Then, the flask contents were poured into a solution prepared by dissolving 5 g of NaOH in a 20 mL water/ice mixture. The crude product was extracted with CHCl_3_ (4 × 20 mL) and purified by column chromatography (silica/ethyl acetate). Then, 0.10 g of the resulting diethyl ester of 1*H*-indolin-2-ylphosphonic acid (compound **3**, yield 50%) was refluxed for 4 h with 8 mL of a 1:1 mixture of concentrated HCl and H_2_O. Then, the volatile residues were removed by a rotary evaporator, and the solid material was heated with 3 mL of anhydrous EtOH. After cooling, the 1*H*-indolin-2-ylphosphonic acid crystalized from the dry ethanol. The solids were filtered, washed with cooled dry ethanol, and dried.

1*H*-indolin-2-ylphosphonic acid (**4**) was obtained as a white solid (60 mg, 78% of yield; m.p. 217–218 °C; ^31^P-NMR (202.46 MHz, D_2_O + NaOD) δ = 18.47 ppm; ^1^H-NMR (500.14 MHz, D_2_O + NaOD) δ = 3.17–3.31 (m, 2H, C*H*_2_CHP), 3.69–3.74 (m, 1H, CH_2_C*H*P), 6.85 (t, 1H, *J* = 9.35 Hz, Ar*H*), 6.86 (t, 1H, *J* = 7.35 Hz, Ar*H*), 7.14 (t, 1H, *J* = 7.60 Hz, Ar*H*), 7.24 (d, 1H, *J* = 7.25 Hz, Ar*H*) ppm; ^13^C-NMR (125.76 MHz, D_2_O + NaOD) δ = 32.11, 56.87 (d, *J* = 137.07 Hz, *C*P), 111.15, 119.63, 124.91, 127.23, 131.09, 150.84; HRMS (ESI + TOF) *m*/*z*: [M − H]^+^. Calcd. for C_8_H_10_NO_3_P: 198.0320; found: 198.0318.

#### 4.3.3. General Procedure for the Preparation of Benzo[*b*]tiophen-2-ylphosphonic Acid (**8**), Tri-2-benzofuryl Phosphine Oxide (**13**), Benzofuran-2-ylphosphonic Acid (**14**), Tri-2-furyl oxide (**15**), 5-Methyltiophen-2-ylphosphonic Acid (**17**), Phenylphosphonic Acid (**24**), 3-Methoxyphenylphosphonic Acid (**25**), and Aphtha-1-ylphosphonic Acid (**26**)

To the mixture of an appropriate substrate (20 mmol of benzotiophene, benzofuran, furan, 2-methyltiophene, 1-bromo-naphthalene, bromobenzene, or 3-bromoanisole) dissolved in 30 mL anhydrous THF and cooled to −70 °C, BuLi (16.2 mL, 26.0 mmol, 1.6 M in hexane) was added dropwise for 30 min under a stream of N_2_. Then, the resulting solution or suspension was stirred for 30 min at −70 °C followed by the dropwise addition of a solution of 20 mmol of ClP(OEt)_2_ (3.13 g, 2.85 mL) in 30 mL of dry THF, maintaining the temperature below −65 °C. After the addition was complete, the reaction was continued for 60 min, and then the cooling bath was removed. When the temperature of the mixture reached −30 °C, 5 mL of 30% aqueous solution of hydrogen peroxide was added very cautiously drop by drop, and the mixture was allowed to warm to room temperature. The resulting solution was poured into 200 mL of water. The crude phosphonic ester was extracted with dichloromethane (3 × 20 mL). The organic layers were combined, dried over anhydrous MgSO_4_, filtered, and evaporated under reduced pressure. The products were purified by column chromatography (silica/diethyl ether or ethyl acetate) or used directly in the next step. The aryl derivatives were hydrolyzed by refluxing in diluted hydrochloric acid (20%), and the crude products were recrystallized from water. Phosphonate esters of heterocyclic derivatives were deprotected by application of TMSBr. Thus, 10.0 mmol of the ester was mixed with dry CH_2_Cl_2_ and 40 mmol (6.12 g) of TMSBr and stirred for 72 h. The volatile components were evaporated under reduced pressure to give a solid residue to which 15 mL of MeOH was added, and the mixture was stirred for 1 h at room temperature. The precipitated product was filtered off, washed with MeOH and Et_2_O, and recrystallized from anhydrous ethanol.

Benzo[*b*]tiophen-2-ylphosphonic acid (**8**) was obtained as creamy prisms (36% of yield); m.p. 198–199 °C; ^31^P-NMR (202.46 MHz, D_2_O + NaOD) δ = 4.33 ppm; ^1^H-NMR (500.14 MHz, D_2_O + NaOD) δ = 7.22–7.28 (m, 2H, Ar*H*), 7.44 (d, 1H, *J* = 7.95 Hz, Ar*H*), 7.76 (d, 1H, *J* = 7.45 Hz, Ar*H*), 7.81 (d, 1H, *J* = 7.70 Hz, Ar*H*) ppm; ^13^C-NMR (125.76 MHz, D_2_O + NaOD) δ = 122.45 (d, *J* = 1.79 Hz), 123.99, 124.30, 124.60, 126.78 (d, *J* = 9.39 Hz), 140.15 (d, *J* = 14.91Hz, *C*S), 141.43 (d, *J* = 6.36 Hz, *C*HCP), 144.17 (d, *J* = 170.89 Hz, ***C***P); HRMS (ESI + TOF) *m*/*z*: [M − H]^+^. Calcd. for C_8_H_7_O_3_P: 212.9775; found: 212.9774. 

#### 4.3.4. Mixture of Diethyl Benzofuran-2-ylphosphonate (**10**) and Diethyl Benzofuran-2-ylphosphinate (**11**) 

After work-up with water, the mixture of products was extracted to dichloromethane (3 × 20 mL), dried over anhydrous MgSO_4_, filtered, and evaporated under reduced pressure. This resulted in a mixture of an oily and crystalline product. The first fraction of crystalline product (mixture of compound **12** and **13**) was isolated by treatment of the mixture with methanol and filtration. The remaining oil was purified by column chromatography (silica/diethyl ether), which resulted in the separation of the desired compound **10** from the mixture of **11** and **12**.

The mixture of products **10** (major one) and **11** (minor one) was obtained as a yellow oil (0.90 g, 18%); ^31^P-NMR (151.02 MHz, CDCl_3_) δ = 4.57 and −0.79 ppm (respectively); ^1^H-NMR (600.58 MHz, CDCl_3_) δ = 1.368 and 1.369 (t each, *J*_1_ = 7.07 Hz, minor, OCH_2_C*H*_3_), 1.40 (t, *J* = 7.07 Hz, major, OCH_2_C*H*_3_), 4.05–4.31 (m, both compounds, OC*H*_2_CH_3_), 7.330 (t, *J* = 7.51 Hz, minor, Ar*H*), 7.334 (t, *J* = 7.50 Hz, major, Ar*H*), 7.44 (t, *J* = 7.75 Hz, minor, Ar*H*), 7.46 (t, *J* = 7.75 Hz, major, Ar*H),* 7.53 (dd, *J*_1_ = 2.67 Hz, *J*_2_ = 0.73 Hz, major, Ar*H*), 7.57 (dd, *J*_1_ = 3.42 Hz, *J*_2_ = 0.72 Hz, minor, Ar*H*), 7.59 (bd, *J* = 8.38 Hz, minor, Ar*H*), 7.60 (bd, *J* = 8.38 Hz, major, Ar*H*), 7.71 (bd, *J* = 7.8 Hz, both compounds, Ar*H*); ^13^C-NMR (151.02 MHz, CDCl_3_) δ = 16.16 (d, *J* = 6.63 Hz, minor, POCH_2_*C*H_3_), 16.29 (d, *J* = 6.40 Hz, major, POCH_2_*C*H_3_), 63.22 (d, *J* = 5.36 Hz, major, PO*C*H_2_CH_3_), 63.64 (d, *J* = 5.66 Hz, minor, PO*C*H_2_CH_3_), 112.14 (minor), 112.18 (major), 118.53 (d, *J* = 17.54 Hz, minor); 119.05 (d, *J* = 24.12 Hz, major), 122.48 (major), 122.50 (minor), 123.61 (major), 123.67 (minor), 126.92 (minor), 127.00 (major), 145.97 (d, *J* = 236.47 Hz, major, ***C***P), 148.00 (d, *J* = 214.03 Hz, minor, ***C***P) ppm; HRMS (ESI + TOF) *m*/*z*: [M + H]^+^. Calcd. for C_12_H_15_O_4_P (**10**): 255.0786; found: 255.0778; 277.0663 (M + Na); 531.1357 (2M + Na).

The mixture of tri-2-benzofuryl phosphine and its oxide (**12** and **13**) was obtained as white crystals (1.75 g, 62%); m.p. 210–211 °C; ^31^P-NMR (243.12 MHz, CDCl_3_) δ = −67.73 and −8.26 ppm; ^1^H-NMR (600.58 MHz, CDCl_3_) δ = 7.27 (td, 1H, *J*_1_ = 7.15 Hz, *J*_2_ = 0.70 Hz, Ar*H*), 7.30 (dd, 1H, *J*_1_ = 1.83 Hz, *J*_2_ = 0.89 Hz, Ar*H*), 7.36 (m, 2H, Ar*H*), 7.48 (t, 1H, *J* = 7.52 Hz, Ar*H*), 7.57 (dd, 1H, *J*_1_ = 8.37 Hz, *J*_2_ = 0.62 Hz, Ar*H*), 7.61 (dd, 1H, *J*_1_ = 1.57 Hz, *J*_2_ = 0.61 Hz, Ar*H*), 7.63 (m, 1H, Ar*H*), 7.72 (dd, 1H, *J*_1_ = 2.78 Hz, *J*_2_ = 0.76 Hz, Ar*H*) 7.74 (d, 1H, *J* = 7.84 Hz, Ar*H*) ppm; ^13^C-NMR (151.02 MHz, CDCl_3_) δ = 111.76, 112.43, 118.39 (d, *J* = 22.39 Hz), 120.53 (d, *J* = 20.92 Hz), 121.47, 122.75, 123.04, 123.88, 125.60, 126.50 (d, *J* = 10.02Hz), 127.53, 127.84 (d, *J* = 6.42 Hz), 147.09 (d, *J* = 152.99 Hz, *C*P(O)), 150.88 (d, *J* = 4.98 Hz, *C*P), 158.14 (d, *J* = 5.48 Hz, *C*OCP), 158.09 (d, *J* = 11.61 Hz, *C*OCP(O)) ppm; HRMS (ESI + TOF) *m*/*z*: [M + H]^+^. Calcd. for C_24_H_15_O_4_P: 399.0786; found: 399.0792.

Tri-2-benzofuryl phosphine oxide (**13**) was obtained as a white solid (24%) ^31^P-NMR (161.83 MHz, CDCl_3_) δ = −7.63 ppm; ^1^H-NMR (399.78 MHz, CDCl_3_) δ = 7.32 (ddd, 1H, *J*_1_ = 0.95 Hz, *J*_2_ = 7.20 Hz, *J*_3_ = 8.03 Hz, Ar*H*), 7.42–7.46 (m, 1H, Ar*H*), 7.568 (ddd, 1H, *J*_1_ = 0.88 Hz, *J*_2_ = 1.73 Hz, *J*_3_ = 8.44 Hz, Ar*H*), 7.67 (dd, 1H, *J*_1_ = 0.98 Hz, *J*_2_ = 2.86 Hz, Ar*H*), 7.67–7.71 (m, 1H, Ar*H*) ppm; ^13^C-NMR (100.53 MHz, CDCl_3_) δ = 112.50 (d, *J* = 1.16 Hz), 120.62 (d, *J* = 21.01 Hz), 122.84, 123.96 (d, *J* = 1.15 Hz), 126.56 (d, *J* = 10.07 Hz), 127.61, 147.13 (d, *J* = 153.07 Hz, ***C***P), 158.16 (d, *J* = 9.29 Hz) ppm; HRMS (ESI + TOF) *m*/*z*: [M + H]^+^. Calcd. for C_24_H_15_O_4_P: 399.0786; found: 399.0854; [M + Na]^+^ 421.0623.

Benzofuran-2-ylphosphonic acid (**14**) was obtained as orange crystals (0.90 g, 46%); decomposed around 400^o^C; ^31^P-NMR (161.83 MHz, D_2_O) δ = 0.62 ppm; ^1^H-NMR (399.78 MHz, D_2_O) δ = 7.17 (dd, *J*_1_ = 0.95 Hz, *J*_2_ = 2.66 Hz, 1H, Ar*H*), 7.23 (td, *J*_1_ = 0.97 Hz, *J*_2_ = 7.62 Hz, 1H, Ar*H*), 7.32–7.36 (m, 1H, Ar*H*), 7.52 (dd, *J*_1_ = 0.84 Hz, *J*_2_ = 8.36 Hz, 1H, Ar*H*), 7.64 (d, *J* = 7.72 Hz, 1H, Ar*H*), ppm; ^13^C-NMR (100.53 MHz, D_2_O) δ = 111.82 (d, *J* = 1.27 Hz), 113.70, 113.92, 122.39, 123.38, 126.13, 127.02 (d, *J* = 11.08 Hz), 152.72 (d, *J* = 217.97 Hz, ***C***P), 156.41 (d, *J* = 10.56 Hz, ***C***OCP) ppm; HRMS (ESI + TOF) *m*/*z*: [M − H]^−^. Calcd. for C_8_H_7_O_4_P: 197.0004; found: 197.0001; [2M − H] 395.0263.

Tri-2-furyl oxide (**15**) was obtained as a white solid (0.6 g, 12%); ^31^P-NMR (161.83 MHz, CDCl_3_) δ = −11.04 ppm; ^1^H-NMR (399.78 MHz, CDCl_3_) δ = 6.54 (dt, 1H, *J*_1_ = 1.66 Hz, *J*_2_ = 3.40 Hz, Ar*H*), 7.15 (ddd, 1H, *J*_1_ = 0.72 Hz, *J*_2_ = 2.05 Hz, *J*_3_ = 3.49 Hz, Ar*H),* 7.72 (ddd, 1H, *J*_1_ = 0.72. Hz, *J*_2_ = 1.70 Hz, *J*_3_ = 2.53 Hz, Ar*H)* ppm; ^13^C-NMR (100.53 MHz, CDCl_3_) δ = 111.15, (d, *J* = 9.41 Hz), 123.60 (d, *J* = 21.05 Hz), 146.02 (d, *J* = 158.84 HZ, ***C***P), 148.98 (d, J = 8.81 Hz) ppm; HRMS (ESI + TOF) *m*/*z*: [M + H]^+^. Calcd. for C_12_H_9_O_4_P: 249.0317; found: 249.0354; [M + Na]^+^ 271.0129.

Diethyl 2-Methylthiophen-5-yl-phosphonate *(***16***)* was obtained as a yellow oil (2.22 g, 48%); ^31^P-NMR (243.12 MHz, CDCl_3_) δ = 12.10 ppm; ^1^H-NMR (600.58 MHz, CDCl_3_) δ = 1.35 (t, 6H, *J* = 7.11 Hz, 6H, OCH_2_C*H*_3_), 2.56 (s, 3H, CC*H*_3_), 4.08–4.19 (m, 4H, OC*H*_2_CH_3_), 6.85 (q, *J*_1_ = 2.65 Hz, 1H Ar*H*), 7.49 (dd, *J*_1_ = 8.52 Hz, *J*_2_ = 3.51 Hz, 1H Ar*H*) ppm; ^13^C-NMR (151.03 MHz, CDCl_3_) δ = 15.26 (d, *J* = 1.38 Hz, C*C*H_3_), 16.21 (d, *J* = 6.87 Hz, POCH_2_*C*H_3_), 62.44 (d, *J* = 5.32 Hz, PO*C*H_2_CH_3_), 124.91(d, *J* = 211.78 Hz, ***C***P), 126.59 (d, *J* = 17.07 Hz), 137.28 (d, *J* = 11.53 Hz), 148.90 (d, *J* = 7.15 Hz) ppm; HRMS (ESI + TOF) *m*/*z*: [M + H]^+^. Calcd. for C_9_H_15_O_3_PS: 235.0558; found: 235.0567; [M + Na]^+^ 257.0345; [2M + H]^+^ 469.0922; [2M + Na]^+^ 491.0674.

5-Methylthiophen-2-ylphosphonic acid (**17**) was obtained as an amorphous solid (65% yield), m.p. 50 °C; ^31^P-NMR (161.98 Hz, CDCl_3_) δ = 15.26 ppm; ^1^H-NMR (400.13 MHz, CDCl_3_) δ = 2.52 (s, 3H, CC*H*_3_), 6.78–6.80 (m, 1H, Ar*H*), 7.43 (dd, *J*_1_ = 3.40 Hz, *J*_2_ = 9.26 Hz, 1H, Ar*H*), 7.96–8.21 (m, 2H, PO_3_*H*_2_) ppm; ^13^C-NMR (151.02 MHz, CDCl_3_) δ = 15.26 (d, *J* = 1.38 Hz, C*C*H_3_), 124.91 (d, *J* = 211.78 Hz, C***C***P), 126.59 (d, *J* = 17.07 Hz), 137.28 (d, *J* = 11.63 Hz), 148.90 (d, *J* = 7.15 Hz) ppm; HRMS (ESI + TOF) *m*/*z*: [M − H]^−^. Calcd. for C_5_H_7_SO_3_P: 176.9775; found: 176.9773; [2M + Na]^-^ 375.5813.

Diethyl phenylphosphonate (**21**) was obtained as a dense oil (66% yield); ^31^P-NMR (202.46 MHz, CDCl_3_) δ = 19.08 ppm; ^1^H-NMR (500.14 MHz, CDCl_3_) δ = 1.23 (t, 6H, *J* = 7.05 Hz, OCH_2_C*H*_3_), 3.93–4.08 (m, 4H, OC*H*_2_), 7.36–7.41 (m, 2H, Ar*H*), 7.45–7.49 (m, 1H, Ar*H*), 7.67–7.72 (m, 2H, Ar*H*) ppm; ^13^C-NMR (125.76 MHz, CDCl_3_) δ = 16.05 (d, *J* = 6.45 Hz, POCH_2_*C*H_3_), 62.25 (d, *J* = 5.57 Hz, PO*C*H_2_), 127.69 (d, *J* = 198.520 Hz, P*C*), 128.46 (d, *J* = 15.08 Hz), 131.53 (d, *J* = 9.96 Hz), 132.51 (d, *J* = 3.04 Hz) ppm; HRMS (ESI + TOF) *m*/*z*: [M + H]^+^. Calcd. for C_6_H_7_O_3_P: 159.0211; found: 159.0209. 

Phenylphosphonic acid (**24**) was obtained as a white solid (51% yield from bromobenzene); m.p. 161–163 °C (lit.[47] m.p. 166); ^31^P-NMR (202.46 MHz, D_2_O + NaOD) δ = 11.31 ppm; ^1^H-NMR (500.14 MHz, D_2_O + NaOD) δ = 7.10–7.24 (m, 3H, Ar*H*), 7.42–7.55 (m, 2H, Ar*H*) ppm; ^13^C-NMR (125.76 MHz, D_2_O + NaOD) δ = 127.70 (d, *J* = 12.56 Hz), 128.71 (d, *J* = 2.15 Hz), 130.07 (d, *J* = 8.75 Hz) ppm; HRMS (ESI + TOF) *m*/*z*: [M − H]^+^. Calcd. for C_6_H_7_O_3_P: 157.0055; found: 157.0048. 

Diethyl 3-methoxyphenylphosphonate (**22**) was obtained as a dense oil (63%, yield); ^31^P-NMR (202.46 MHz, CDCl_3_) δ = 18.64 ppm; ^1^H-NMR (500.14 MHz, CDCl_3_) δ = 1.32 and 1.36 (t, 6H, *J*_1_ = 7.05 Hz, *J*_2_ = 7.10 Hz, OCH_2_C*H*_3_), 3.84 (s, 3H, OC*H*_3_), 4.04–4.18 (m, 4H, OC*H*_2_), 7.07–7.09 (m, 1H, Ar*H*), 7.31–7.35 (m, 1H, Ar*H*), 7.36–7.39 (m, 2H, Ar*H*) ppm; ^13^C-NMR (125.76 MHz, CDCl_3_) δ = 16.30 (d, *J* = 6.49 Hz, POCH_2_*C*H_3_), 55.40 (O*C*H_3_), 62.16 (d, *J* = 5.39 Hz, PO*C*H_2_), 116.37 (d, *J* = 11.36 Hz), 118.72 (d, *J* = 3.18 Hz), 118.72 (d, *J* = 3.18 Hz), 123.93 (d, *J* = 9.28 Hz), 129.55 (d, *J* = 186.89 Hz, ***C***P), 129.73 (d, *J* = 17.58 Hz), 159.43 (d, *J* = 18.89 Hz) ppm; HRMS (ESI + TOF) *m*/*z*: [M + H]^+^. Calcd. for C_11_H_17_O_4_P: 245.0943; found: 245.0940. These data are in good agreement with the literature [48].

3-Methoxyphenylphosphonic *acid* (**25**) was obtained as a brownish solid (50% yield from bromoanisole) m.p. 124–126 °C (lit.[49] m.p. 139–142 °C); ^31^P-NMR (202.46 MHz, D_2_O + NaOD) δ = 10.82 ppm; ^1^H-NMR (500.14 MHz, D_2_O + NaOD) δ = 3.75 (s, 3H, OC*H*_3_), 6.88 (d, 1H, *J* = 7.05 Hz, Ar*H*), 7.19–7.27 (m, 3H, Ar*H*) ppm; ^13^C-NMR (125.76 MHz, D_2_O + NaOD) δ = 55.38 (O*C*H_3_), 114.33 (d, *J* = 2.64 Hz), 115.59 (d, *J* = 9.85 Hz), 123.14 (d, *J* = 8.23 Hz), 129.10 (d, *J* = 14.46 Hz), 142.72 (d, *J* = 165.84 Hz, *C*P), 158.04 (d, *J* = 15.82 Hz) ppm; HRMS (ESI + TOF) *m*/*z*: [M − H]^+^. Calcd. for C_7_H_9_O_4_P: 187.0160; found: 187.0169. 

Naphth-1-ylphosphonic acid (**26**) was obtained as a white solid (56% yield from 1-bromonaphtalene) m.p. 203–204 °C (lit. [50] m.p. 204–206 °C); ^31^P-NMR (202.46 MHz, D_2_O + NaOD) δ = 9.09 ppm; ^1^H-NMR (500.14 MHz, D_2_O + NaOD) δ = 7.34 (t, 2H, *J* = 7.40 Hz, Ar*H*), 7.42 (t, 1H, *J* = 7.40 Hz, Ar*H*), 7.75 (d, *J* = 8.10 Hz, Ar*H*), 7.83 (dd, *J* = 6.90 Hz, *J* = 13.85 Hz, PC = C*H*), 8.59 (d, 1H, *J* = 8.45 Hz, Ar*H*) ppm; ^13^C-NMR (125.76 MHz, D_2_O + NaOD) δ = 125.09 (d, *J* = 13.69 Hz), 125.65 (d, *J* = 19.86 Hz), 128.24, 128.62 (d, *J* = 4.55 Hz), 129.41 (d, *J* = 2.64 Hz), 129.99 (d, *J* = 7.58 Hz), 132.97 (d, *J* = 8.79 Hz), 133.28 (d, *J* = 10.16 Hz), 137.70 (d, *J* = 163.22 Hz, *C*P) ppm; HRMS (ESI + TOF) *m*/*z*: [M − H]^+^. Calcd. for C_10_H_9_O_3_P: 207.0211; found 207.0212. 

#### 4.3.5. Preparation of Benzo[*b*]thiazol-2-ylphosphonic Acid (**7**)

The mixture of 30 mL anhydrous THF containing 16 mmol of BuLi (16.2 mL, 1.6 M in hexane) was placed in a round-bottom flask and cooled to −70 °C, while 20 mmol of benzothiazole (2.70 g) was added dropwise under a stream of N_2_. The resulting yellow suspension of lithium salt was stirred for an additional 30 min at −70 °C. In the meantime, in a separate flask, 20 mmol of ClP(OEt)_2_ (3.13 g) in 30 mL dry THF was prepared and cooled in an ice bath, and the suspension of the lithium salt to the solution of ClP(OEt)_2_ in one batch was added to this solution. The resulting orange solution was stirred for the next 30 min, while the temperature was maintained at 0 °C followed by the dropwise addition of 3 mL of 30% aqueous solution of H_2_O_2_, which resulted in the appearance of a yellow precipitate. The reaction was continued for an additional 1 h. After that, the flask contents were poured into 200 mL of cold water, and the crude phosphonic ester was extracted with diethyl ether (3 × 20 mL). The organic phases were combined, dried over anhydrous MgSO_4_, filtered, and evaporated under reduced pressure. The product was purified by column chromatography (SiO_2_/Et_2_O) to yield 3.24 g (60%) of diethyl benzo[*b*]thiazol-2-ylphosphonate, which was used immediately in the next reaction. Thus, 10.0 mmol (2.71 g) of the resulting ester was mixed with dry CH_2_Cl_2_ and 40 mmol (6.12 g) of TMSBr and stirred for 72 h. The volatile components were evaporated under reduced pressure to give a solid residue to which 15 mL of MeOH was added, and the mixture was stirred for 1 h at room temperature. The precipitated product was filtered off, washed with MeOH and Et_2_O, and dried. 

Compound **7** was obtained as a yellowish solid (84% yield (total yield 50%) m.p. 231–232 °C (lit.[51] m.p. 161–163 °C); ^31^P-NMR (202.46 MHz, D_2_O + NaOD) δ = 0.14 ppm; ^1^H-NMR (500.14 MHz, D_2_O + NaOD) δ = 7.34 (t, 1H, *J* = 7.40 Hz, Ar*H*), 7.42 (t, 1H, *J* = 7.10 Hz, Ar*H*), 7.93 (t, 2H, *J* = 7.50 Hz, Ar*H*) ppm; ^13^C-NMR (125.76 MHz, D_2_O + NaOD) δ = 122.26, 122.43, 125.57, 126.23, 135.45, 153.76 (d, *J* = 20.00 Hz), 176.30 (d, *J* = 187.65 Hz, *C*P) ppm; HRMS (ESI + TOF) *m*/*z*: [M − H]^+^. Calcd. for C_7_H_6_SNO_3_P: 213.9728; found: 213.9728.

#### 4.3.6. Preparation of Ethyl Bis(*N*-methylpyrrol-2-yl)phosphinate (**19**)

To the mixture of *N-*methylpyrrole (0.81 g, 10 mmol) and TMEDA (1.42 g, 15 mmol) in 15 mL of dry diethyl ether, a solution of *n*-butyllithium (10.6 mL, 17 mmol, 1.6 M in hexane) was added dropwise, while a slow stream of N_2_ was passed through. Then, the resulting solution was refluxed for 1 h, and ClP(OEt)_2_ (1.56 g, 10 mmol) in 5 mL of Et_2_O was added dropwise with cooling in an ice bath. The reaction was then continued for 8 h at room temperature, and the solution was once again cooled in an ice bath followed by the slow addition of 3 mL of a 30% aqueous solution of H_2_O_2_. When the addition was complete, the flask contents were poured onto 30 mL of water. The crude product containing mostly compound **19** was extracted with CH_2_Cl_2_ (3 × 20 mL) and purified by column chromatography (silica/AcOEt) to yield 0.66 g (52%) of ethyl bis(1-methylpyrrol-2-yl)phosphinate as a colorless oil. ^31^P-NMR (202.46 MHz, CDCl_3_): δ = 14.92 ppm. ^1^H-NMR (500.14 MHz, CDCl_3_): δ = 1.30 (t, 3H, *J* = 7.05 Hz, POCH_2_CH_3_), 3.68 (s, 6H, NC*H*_3_), 4.08–4.13 (m, 2H, POC*H*_2_), 6.03–6.06 (m, 2H, Ar*H*); 6.53 (td, 2H, *J* = 3.62 Hz, *J* = 1.70 Hz, Ar*H*), 6.73–6.74 (m, 2H, Ar*H*) ppm; ^13^C-NMR (125.76 MHz, CDCl_3_): 16.47 (d, *J* = 6.99 Hz), 36.09, 61.36 (d, *J* = 5.51 Hz), 108.20 (d, *J* = 13.42 Hz), 121.32 (d, *J* = 17.79 Hz), 122.48 (d, *J* = 179.35 Hz, ***C***P), 129.38 (d, *J* = 6.95 Hz) ppm; HRMS (ESI + TOF) *m*/*z*: [M + H]^+^. Calcd. For C_12_H_17_N_2_O_2_P: 253.1106; found: 253.1112.

For all the spectral data, see the Supplementary Information.

### 4.4. Antiproliferative Activity

#### 4.4.1. Cell Culture

Mycoplasma-free MCF-7, PC-3, and RAW 264.7 cell lines were purchased from the European Collection of Authenticated Cell Cultures (ECACC) and maintained at the Institute of Immunology and Experimental Therapy (IIET), Wroclaw, Poland. The MCF-7 cell line was cultured in an Eagle medium (Life Technologies, Warszawa, Poland), supplemented with 10% (*v/v*) FBS, 2 mM L-glutamine, 1% NEAA, and 0.01 mg/mL insulin (all Sigma-Aldrich, Poznań, Poland). The PC-3 cell line was cultured in RPMI-1640 medium (Life Technologies, Scotland), supplemented with 10% (*v/v*) FBS and 2 mM L-glutamine. The RAW 264.7 cell line was cultured in DMEM (Life Technologies, Scotland), supplemented with 10% (*v/v*) FBS and 2 mM L-glutamine. All culture media were additionally supplemented with 100 µg/mL streptomycin and 100 U/mL penicillin. All cell lines were cultured during all experiments in a humid atmosphere at 37 °C and 5% CO_2_ and passaged twice a week using EDTA-Trypsin (pH 8; IIET, Wroclaw, Poland) solution as a detachment agent.

#### 4.4.2. SRB Antiproliferative Assay

Twenty-four hours before adding the tested compounds, the cells were seeded on 96-well plates (Sarstedt, Germany) in an appropriate culture medium with 0.75 × 105 cells/mL for MCF-7, 105 cells/mL for PC-3, and 0.1 × 105 cells/mL for RAW 264.7. The cells were treated with each compound in at least four concentrations in the range of 1000 µM^–1^ µM for 72 h. The 0.2 M NaOH, used as a stock solution solvent, was tested for antiproliferative activity, and it did not affect the cell proliferation at 1 mM, the highest concentration used in the compound solutions.

This was used as previously described [52], with minor modifications for all adherent cells. Briefly, the cells were fixed with 50 µL/well of 50% (*w/v*) trichloroacetic acid (Avantor Performance Materials, Gliwice, Poland). After 1 h incubation, the plates were washed several times with tap water, and 50 µL of a 0.1% (*w/v*) solution of sulforhodamine B (Sigma-Aldrich, Schnelldorf, Germany) in 1% (*v/v*) acetic acid (Avantor Performance Materials, Gliwice, Poland) was added to each well. After 30 min incubation at room temperature, the unbound dye was washed out with 1% (*v/v*) acetic acid, whereas the bound dye was solubilized with 10 mM of unbuffered TRIS (Avantor Performance Materials, Gliwice, Poland) solution. The entire procedure was performed using a Biotek EL-406 washing station (BioTek Instruments, Winooski, VT, USA). The absorbance was then read using a Biotek Hybrid H4 reader (BioTek Instruments, USA) at a 540 nm wavelength. 

The compounds at each concentration were tested in triplicate in a single experiment, and each experiment was repeated at least three times independently. The results are presented as the mean IC_50_ ± standard deviation (SD) calculated using the Prolab-3 system based on Cheburator 0.4 software [53].

## Figures and Tables

**Table 1:** X-ray crystallography experimental details.

	13	15
Crystal data		
Chemical formula	C_144_H_92_O_22_P_6_	C_12_H_9_O_4_P
*M*_r_	2359.99	248.16
Crystal system, space group	Trigonal, R$\overset{-}{3}$	Orthorhombic, *P*2_1_2_1_2_1_
*a*, *b*, *c* (Å)	30.3245 (9), 30.3245 (9), 10.5270 (3)	7.9719 (2), 10.8701 (3), 13.3094 (4)
α, β, γ (°)	90, 90, 120	90, 90, 90
*V* (Å^3^)	8383.4 (6)	1153.33 (6)
*Z*	3	4
µ (mm^−1^)	0.18	0.24
Crystal size (mm)	0.3 × 0.25 × 0.2	0.4 × 0.3 × 0.2
Data collection		
No. of measured, independent and observed [*I* > 2σ(*I*)] reflections	19,039, 3651, 2079	5500, 2274, 2103
*R*_int_	0.072	0.022
(sin θ/λ)_max_ (Å^−1^)	0.617	0.617
Refinement		
*R*[*F*^2^ > 2σ(*F*^2^)], *wR*(*F*^2^), *S*	0.039, 0.063, 0.83	0.027, 0.066, 1.02
No. of reflections	3651	2274
No. of parameters	271	154
Δρ_max_, Δρ_min_ (e Å^−3^)	0.30, −0.21	0.31, −0.25

**Table 2:** Structures and in vitro antiproliferative activities of the phosphonic acids against RAW 264.7 mouse macrophages, PC-3 human prostate cancer cells, and MCF-7 human breast cancer cells.

Compound	Structure	IC_50_^a^ [μM]		
		RAW 264.7	PC-3	MCF-7
**4**	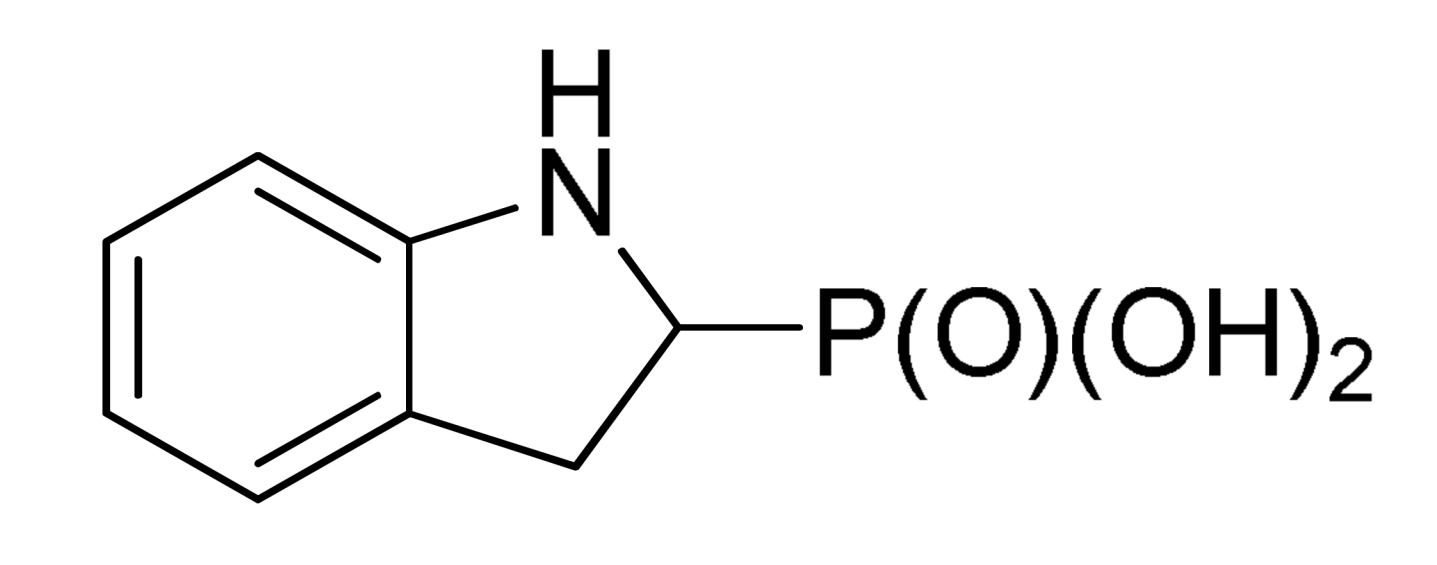	6.49 ± 0.139	4.78 ± 3.69	1.25 ± 0.47
**7**	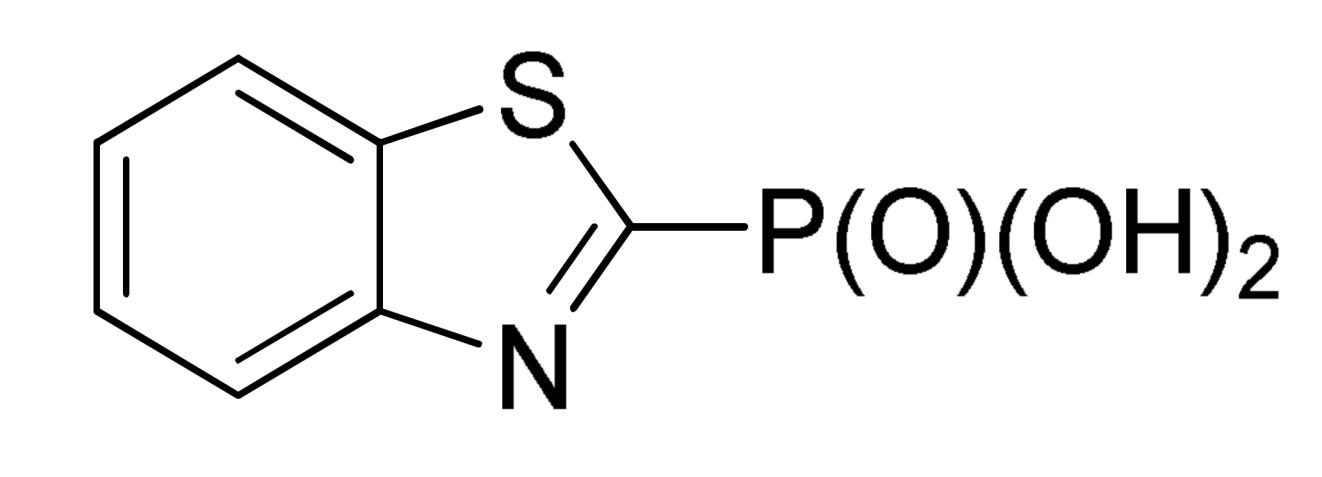	16.95 ± 2.11	4.942± 4.08	5.83 ± 2.26
**8**	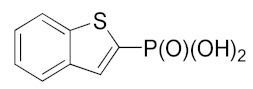	28.07 ± 17.8	16.03 ± 3.40	5.40 ± 1.55
**14**	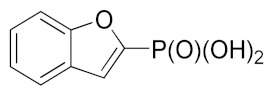	18.21 ± 2.11	3.95 ± 4.18	15.78 ±7.55
**17**	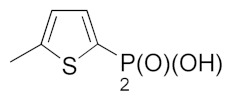	15.08 ± 2.47	10.00 ± 2.83	4.00 ± 2.83
**24**	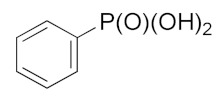	38.91 ± 0.132	6.18 ± 1.02	8.55 ±1.48
**26**	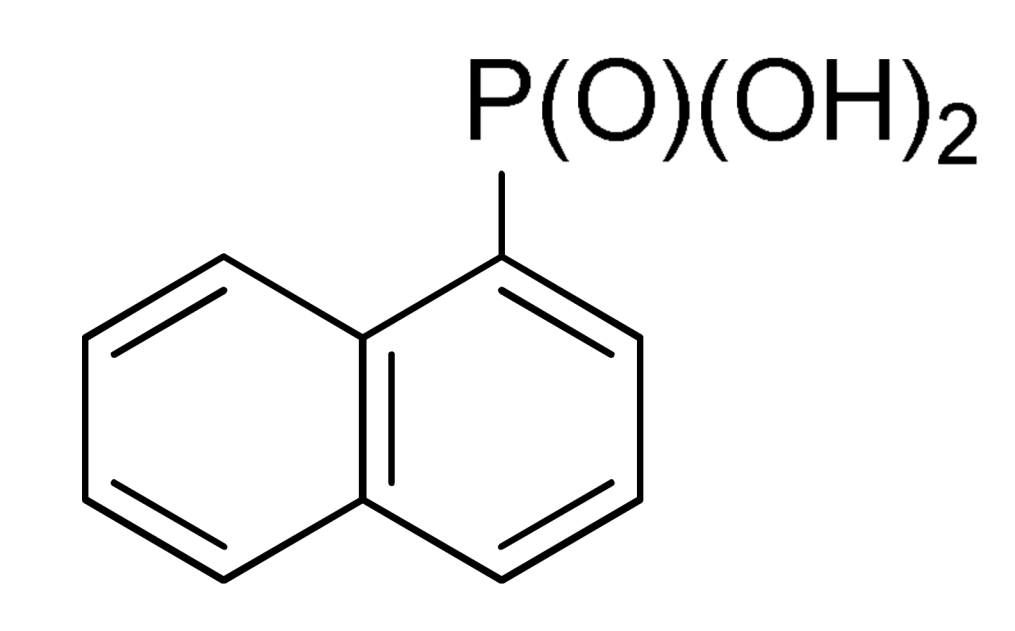	14.05 ± 3.37	3.96 ± 1.63	6.09 ± 1.73
Zoledronic acid	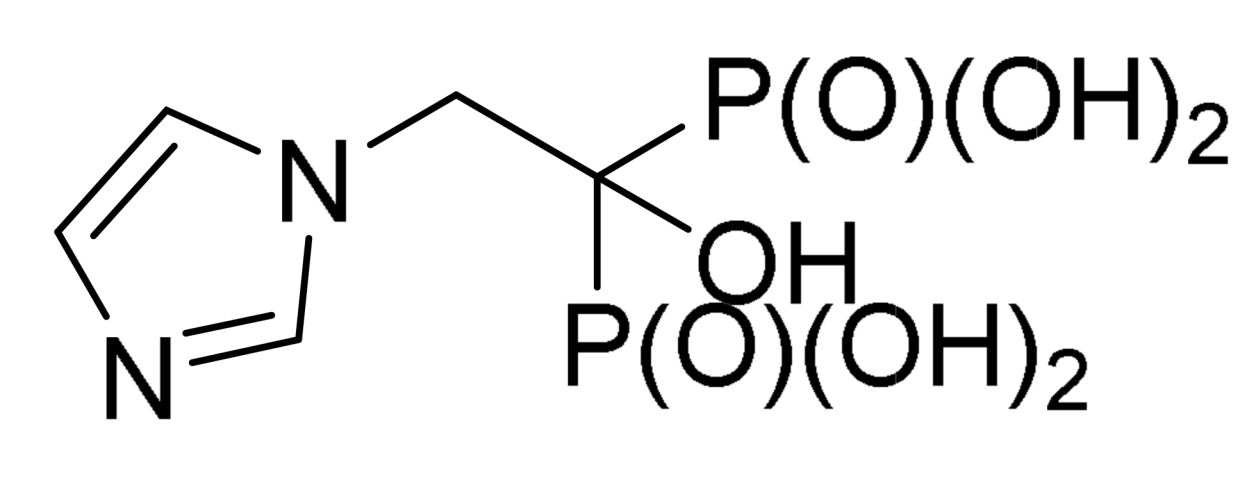	42.2 ± 8.40	146.0 ± 67.4	115.3 ± 87.6
Cisplatin	(H_2_N)_2_PtCl_2_	0.93 ± 0.40	9.83 ± 1.70	6.37 ± 0.80

^a^ IC_50_ values were determined at concentrations in the range of 1–1000 μg/mL. Values are the mean ± standard deviation from at least three experiments, performed in triplicate.

## Data Availability

The data presented in this study and associated additional data are available upon request.

## Supplementary Materials

The following supporting information can be downloaded at: https://www.mdpi.com/article/10.3390/molecules28073135/s1, Figures S1–S77: The ^31^P-NMR, ^1^H-NMR, ^13^C-NMRand HRMS spectra of the representative compounds; Table S1: Relevant crystallographic data for the molecule and the full geometrical information (Å, °); Table S2: Selected hydrogen-bond parameters.
