# Supplementary material for: One-Pot Phosphonylation of Heteroaromatic Lithium Reagents: The Scope and Limitations of Its Use for the Synthesis of Heteroaromatic Phosphonates
Source: Molecules. 2023 Mar 31;28(7):3135. doi: 10.3390/molecules28073135 (PMC10095984; doi:10.3390/molecules28073135)
Supplement: Supplementary file 1 [file molecules-28-03135-s001.zip › molecules-2298233-supplementary.pdf]

## Supporting Information for

# One-Pot Phosphonylation of Heteroaromatic Lithium Reagents: The Scope and Limitations of Its Use for the Synthesis of Heteroaromatic Phosphonates

**Ewa Chmielewska [1,*], Natalia Miodowska [1], Błażej Dziuk [2], Mateusz Psurski [3] and Paweł Kafarski [4]**

[1] Department of Bioorganic Chemistry, Faculty of Chemistry, Wrocław University of Science and Technology, Wybrzeże Wyspiańskiego 27, 50-370 Wrocław, Poland; natalia.miodowska@pwr.edu.pl
[2] Department of Inorganic and Structural Chemistry, Faculty of Chemistry, University of Opole, ul. Oleska 48, 45-052 Opole, Poland; bdziuk@uni.opole.pl
[3] Laboratory of Experimental Anticancer Therapy, Department Of Experimental Oncology, Ludwik Hirszfeld Institute of Immunology and Experimental Therapy Polish Academy of Sciences, Rudolfa Weigla 12, 53-114 Wrocław, Poland; mateusz.psurski@hirszfeld.pl
[4] Department of Chemistry, Faculty of Agriculture and Forestry, University of Warmia and Mazury, Plac Łódzki 4, 10-721 Olsztyn, Poland; pawel.kafarski@uwm.edu.pl
* Correspondence: ewa.chmielewska@pwr.edu.pl; Tel.: +48-71-320 2977

## Copies of $^{31}$P NMR, $^{1}$H NMR, $^{13}$C NMR, MS spectra for representative compounds and relevant crystallographic data for the molecule and the full geometrical information

### Table of contents

***Diethyl 1H-indol-2-ylphosphonate*** **(1)** – $^{31}$P NMR, $^{1}$H NMR, $^{13}$C NMR, MS, ……………………………..……**2**

***1H-indolin-2-ylphosphonic acid*** **(4)** - $^{31}$P NMR, $^{1}$H NMR, $^{13}$C NMR, MS……………………………………**5**

***Benzo[b]tiophen-2-ylphosphonic acid*** **(8)** - $^{31}$P NMR, $^{1}$H NMR, $^{13}$C NMR, MS…………………………....**7**

***Diethyl phenylphosphonate*** **(21)** - $^{31}$P NMR, $^{1}$H NMR, $^{13}$C NMR, MS ……………………………..……..…**9**

***Phenylphosphonic acid*** **(24)** - $^{31}$P NMR, $^{1}$H NMR, $^{13}$C NMR, MS……………………………………………...**11**

***Diethyl 3-methoxyphenylphosphonate*** **(22)** - $^{31}$P NMR, $^{1}$H NMR, $^{13}$C NMR, MS……………………………......…**13**

***3-Methoxyphenylphosphonic acid*** **(25)** - $^{31}$P NMR, $^{1}$H NMR, $^{13}$C NMR, MS…………………………......**16**

***Naphth-1-ylphosphonic acid*** **(26)** - $^{31}$P NMR, $^{1}$H NMR, $^{13}$C NMR, MS………….……………………..**18**

***Benzo[b]thiazol-2-ylphosphonic acid*** **(7)** - $^{31}$P NMR, $^{1}$H NMR, $^{13}$C NMR, MS…………………...….......**20**

***Mixture of compounds* 18, 19 *and* 20** - $^{31}$P NMR, $^{1}$H NMR, $^{13}$C NMR, HMBC(H-P)..……………......……**23**

***Ethyl bis(N-methylpyrrol-2-yl)phosphinate*** **(19)** MS …………………………………………………….………**26**

***Mixture of diethyl benzofuran-2-ylphosphonate (*10*) and diethyl benzofuran-2-ylphosphinate*** **(11)** - $^{31}$P NMR, $^{1}$H NMR, $^{13}$C NMR, MS……………………………………………………………………………………**26**

***Mixture of tri-2-benzofuryl phosphine*** **(12)** ***and its oxide*** **(13)**- $^{31}$P NMR, $^{1}$H NMR, $^{13}$C NMR, C-H, H-P, MS**28**

***Tri-2-benzofuryl phosphine oxide (13)*** - $^{31}$P NMR, $^{1}$H NMR, $^{13}$C NMR, MS………………………………..**32**

***Tri-2-furyl phosphine oxide* (*15*)** $^{31}$P NMR, $^{1}$H NMR, $^{13}$C NMR, MS…………….…………………………….**34**

***Benzofuran-2-ylphosphonic acid* (14) -** $^{31}$P NMR, $^{1}$H NMR, $^{13}$C NMR, MS …………………………..…….**36**

***Diethyl 2-methylthiophen-5-yl-phosphonate* (16) -** $^{31}$P NMR, $^{1}$H NMR, $^{13}$C NMR, MS …………………….**38**

***2-Methylthiophen-5-yl-phosphonic acid* (17) -** $^{31}$P NMR, $^{1}$H NMR, $^{13}$C NMR, MS………………………….**40**

**Relevant crystallographic data for the molecule and the full geometrical information** …………………**43**

**1** 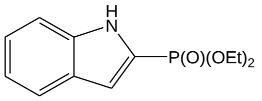


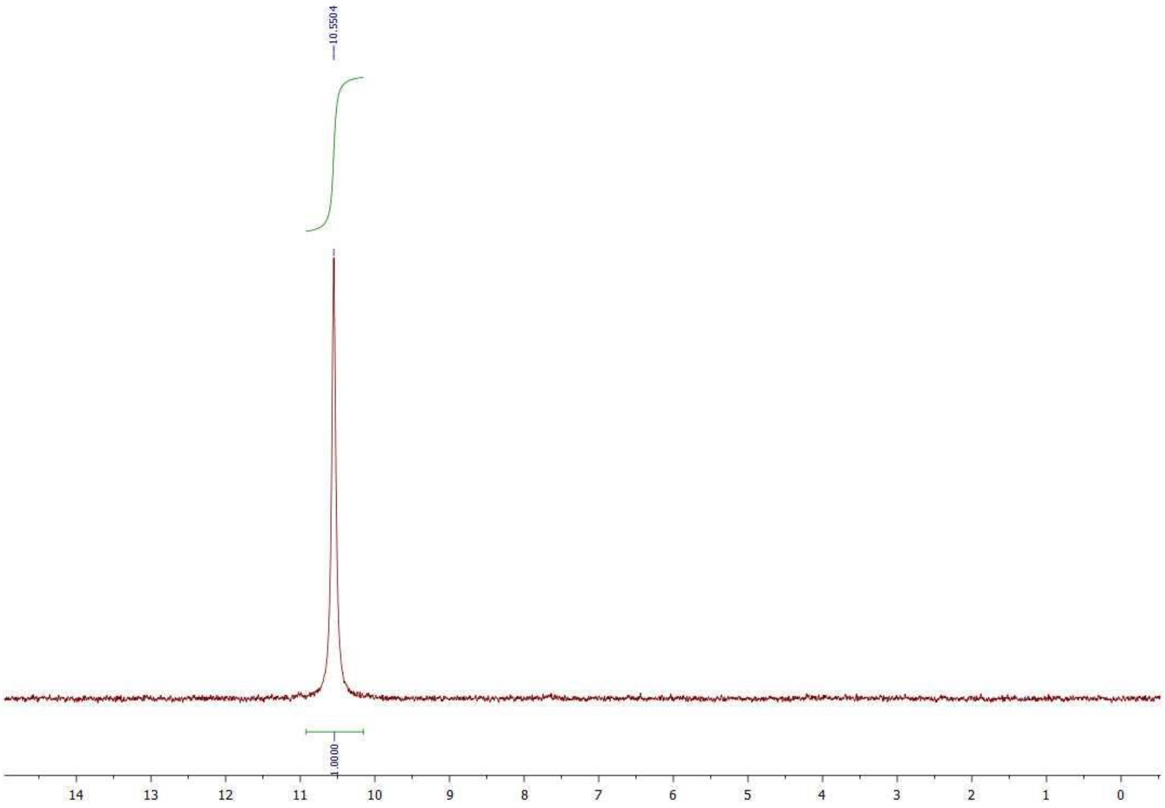


Figure S1: The $^{31}$P NMR spectra of the compound **1**

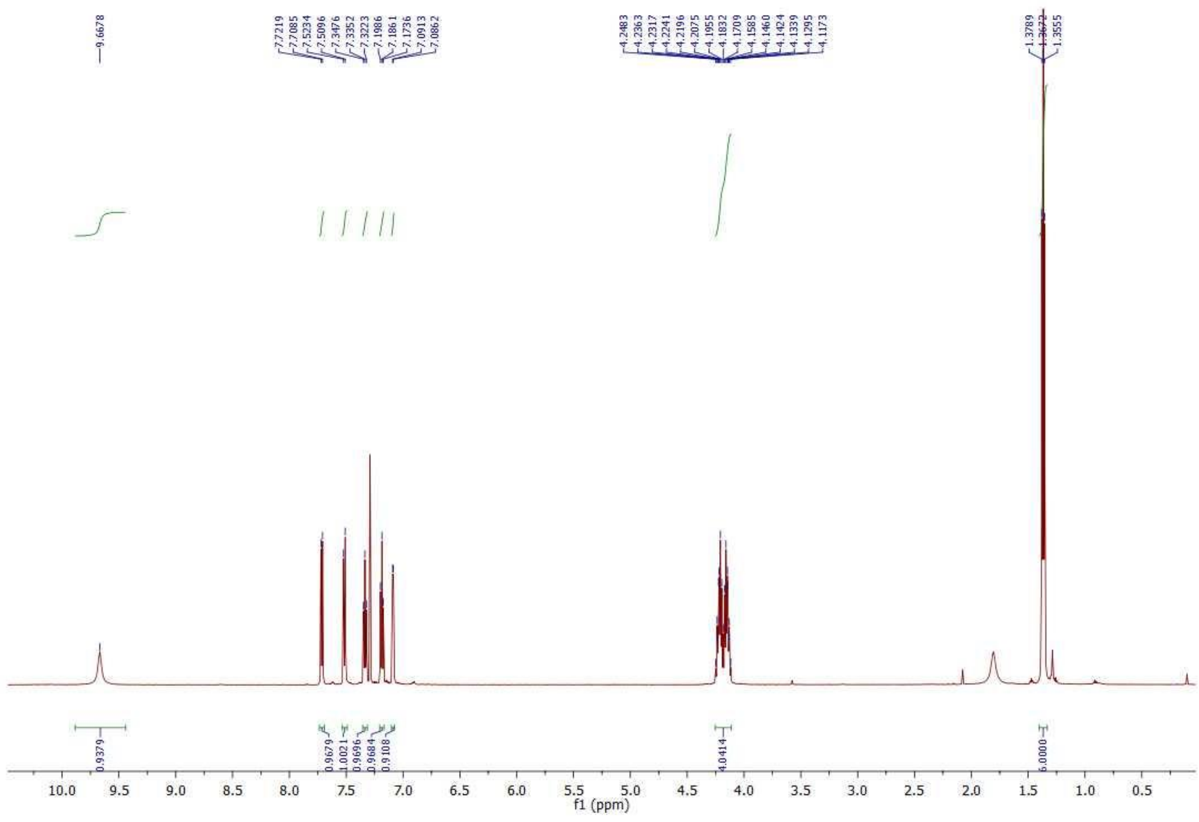

Figure S2: The $^{1}H$ NMR spectra of the compound **1**

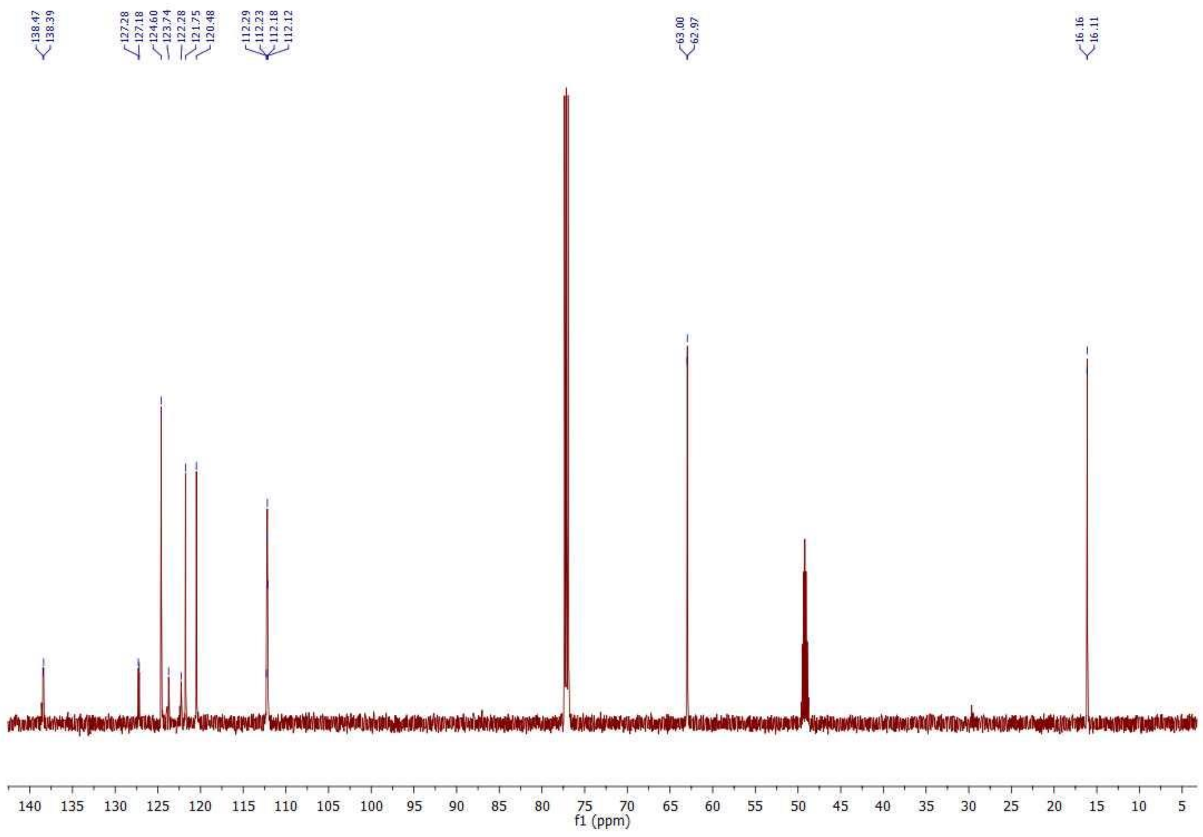


Figure S3: The $^{13}$C NMR spectra of the compound **1**

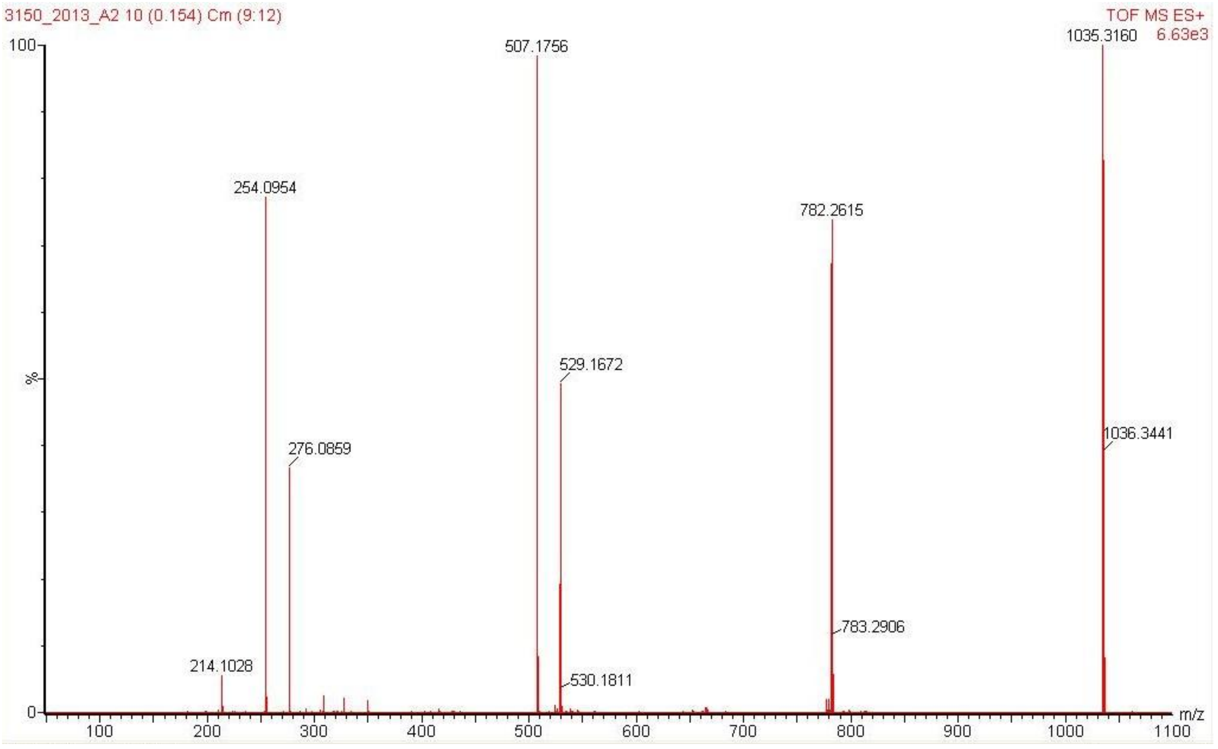


Figure S4: The HRMS spectra of the compound **1**

4

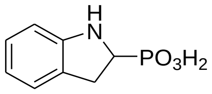


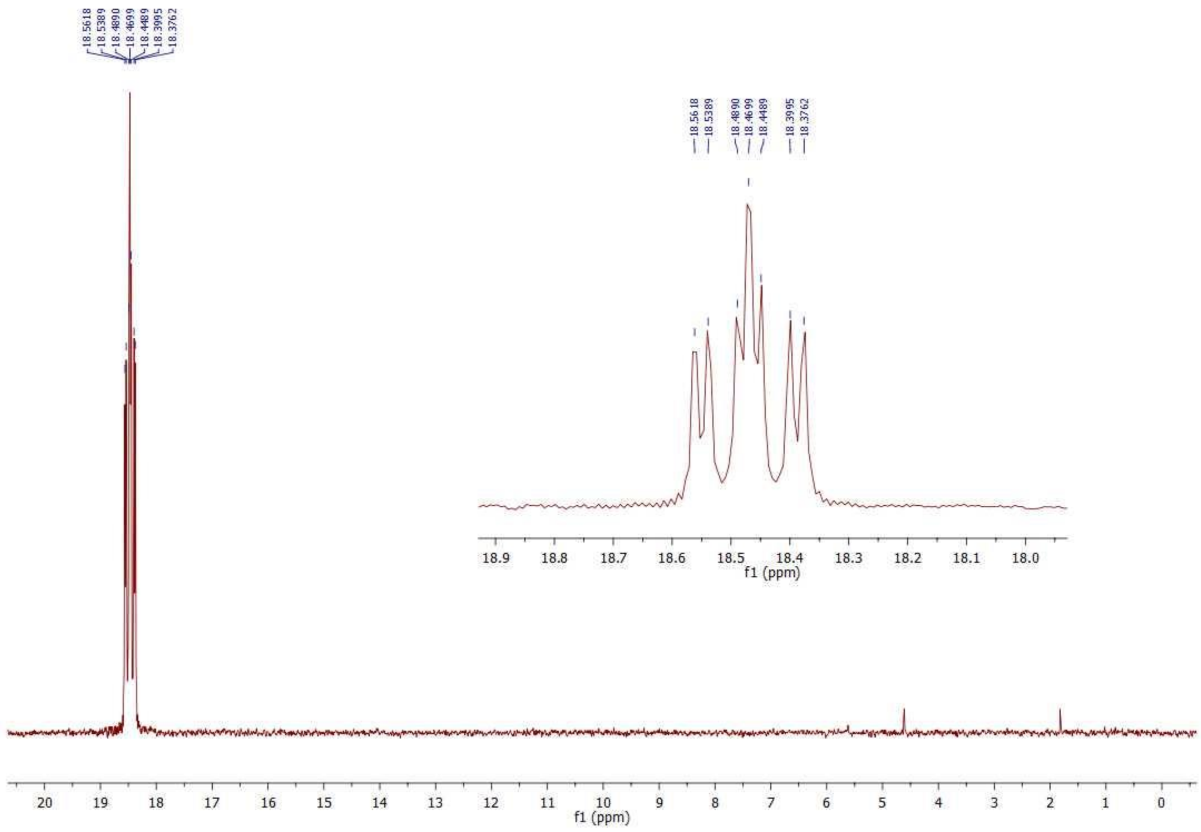


Figure S5: The ${}^{31}P$ NMR spectra of the compound **4**

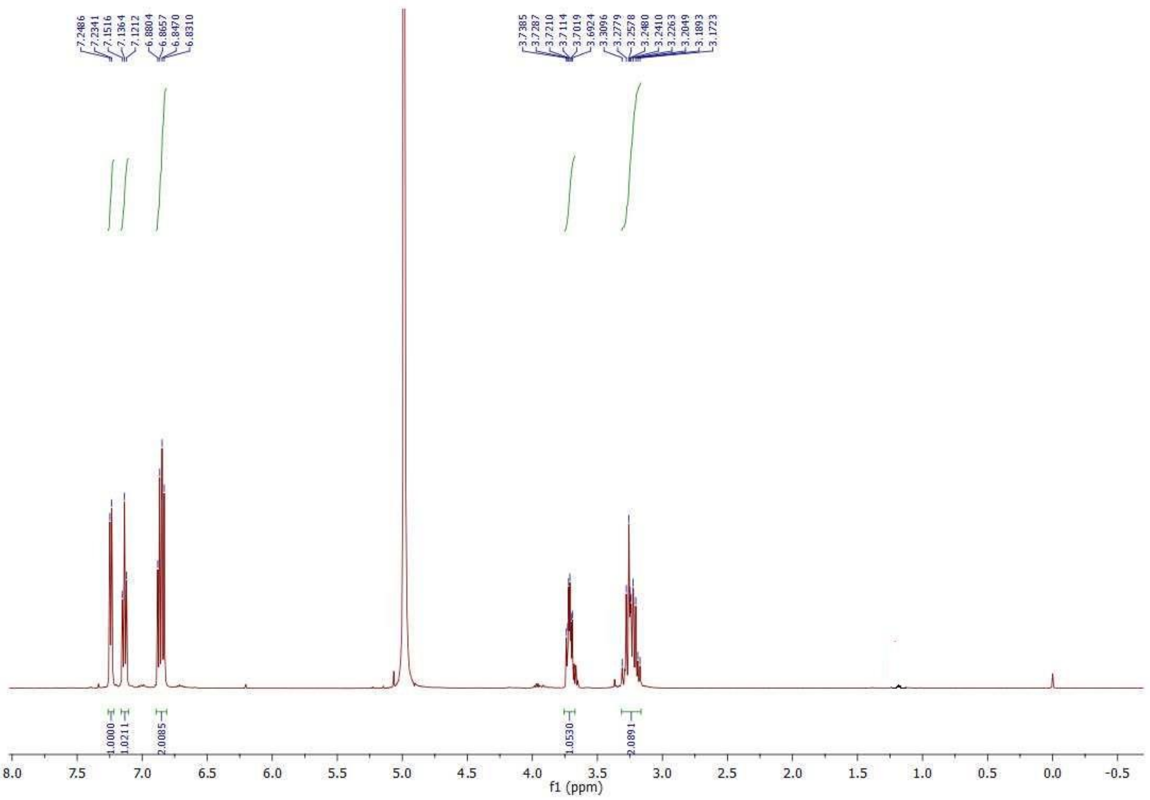


Figure S6: The $^{1}$H NMR spectra of the compound **4**

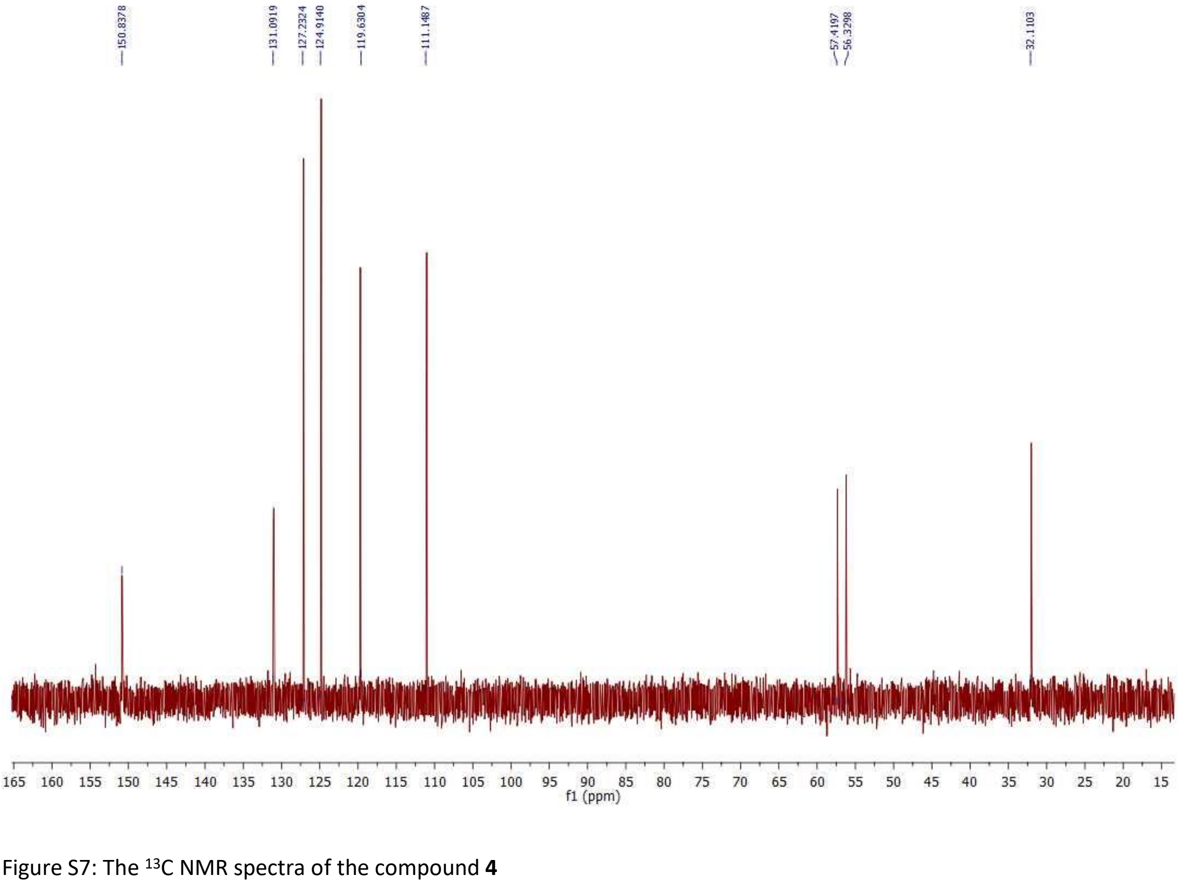


Figure S7: The $^{13}$C NMR spectra of the compound **4**

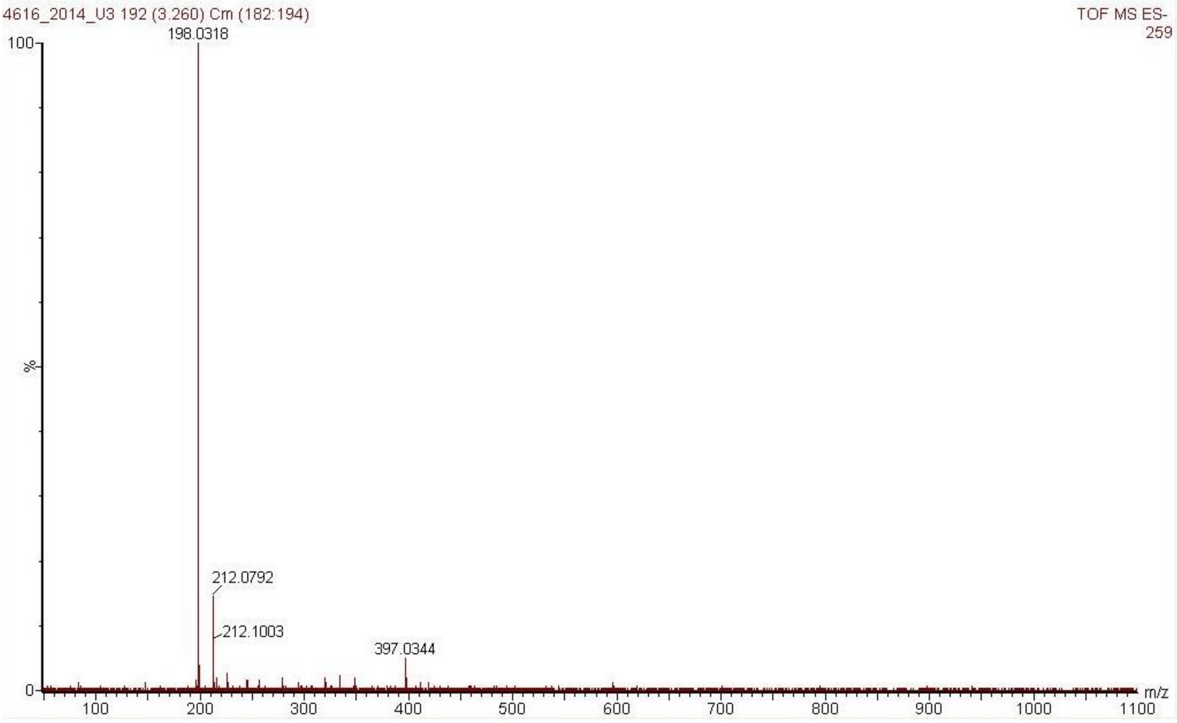


Figure S8: The HRMS spectra of the compound **4**

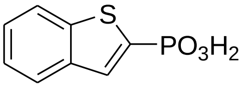


**8**

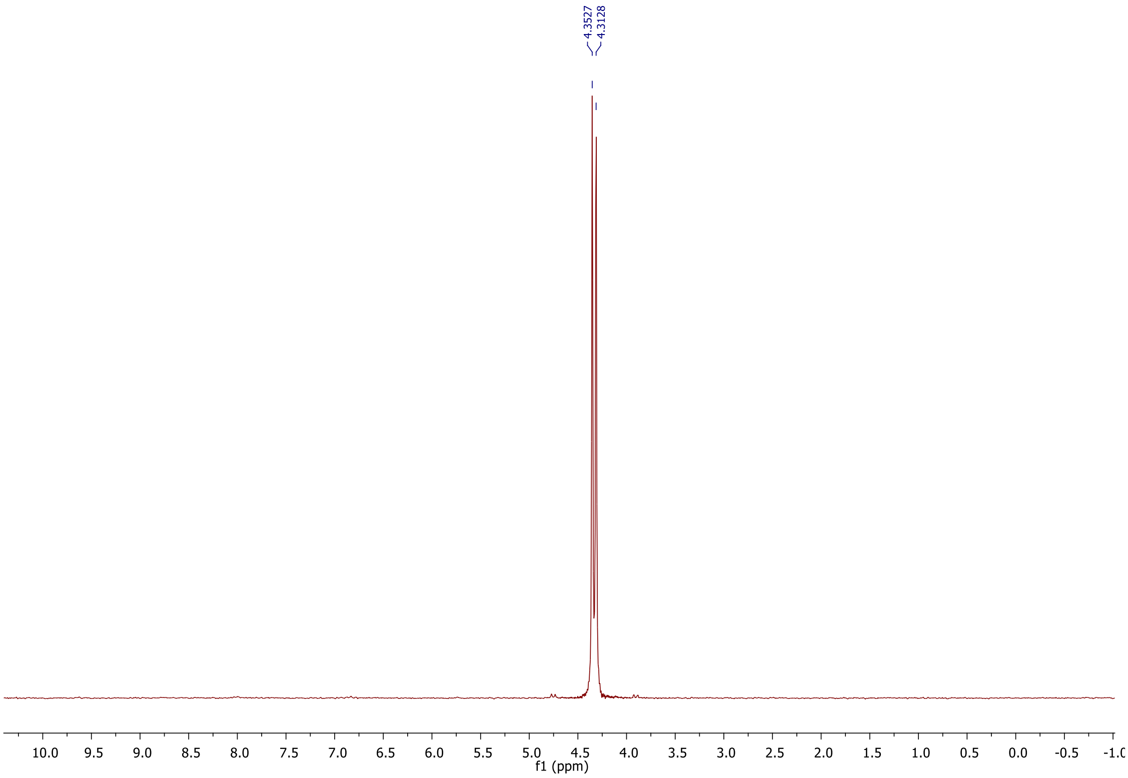


Figure S9: The $^{31}$P NMR spectra of the compound **8**

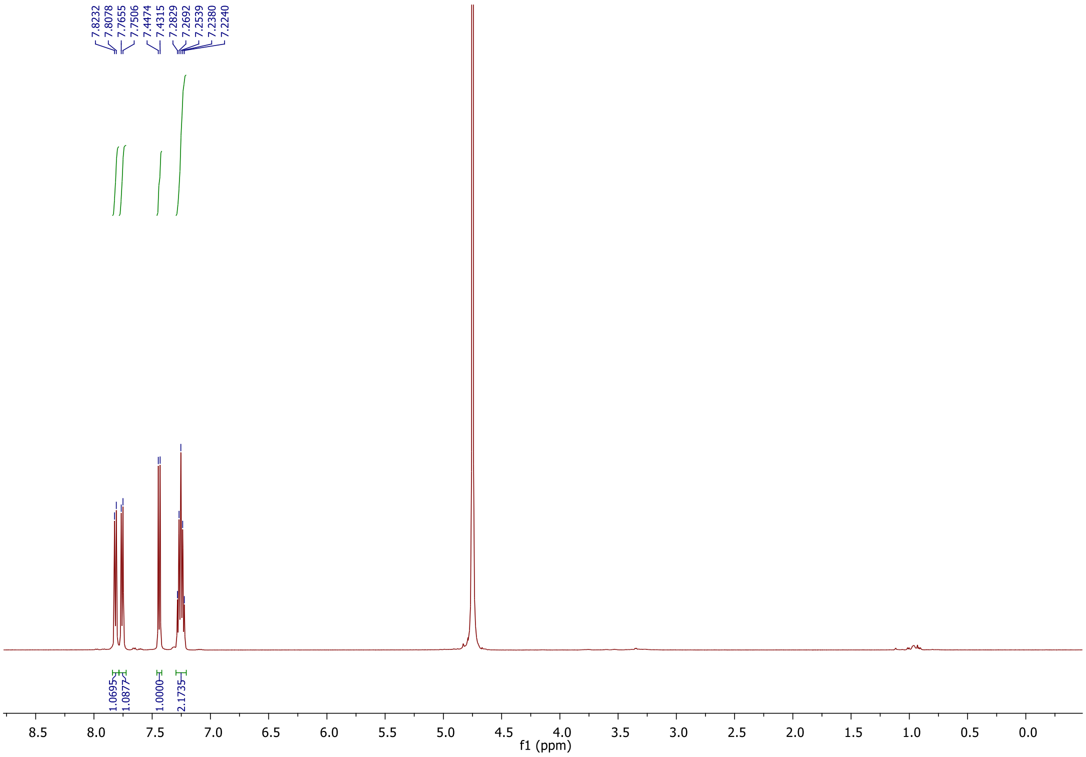


Figure S10: The [1] H NMR spectra of the compound **8**

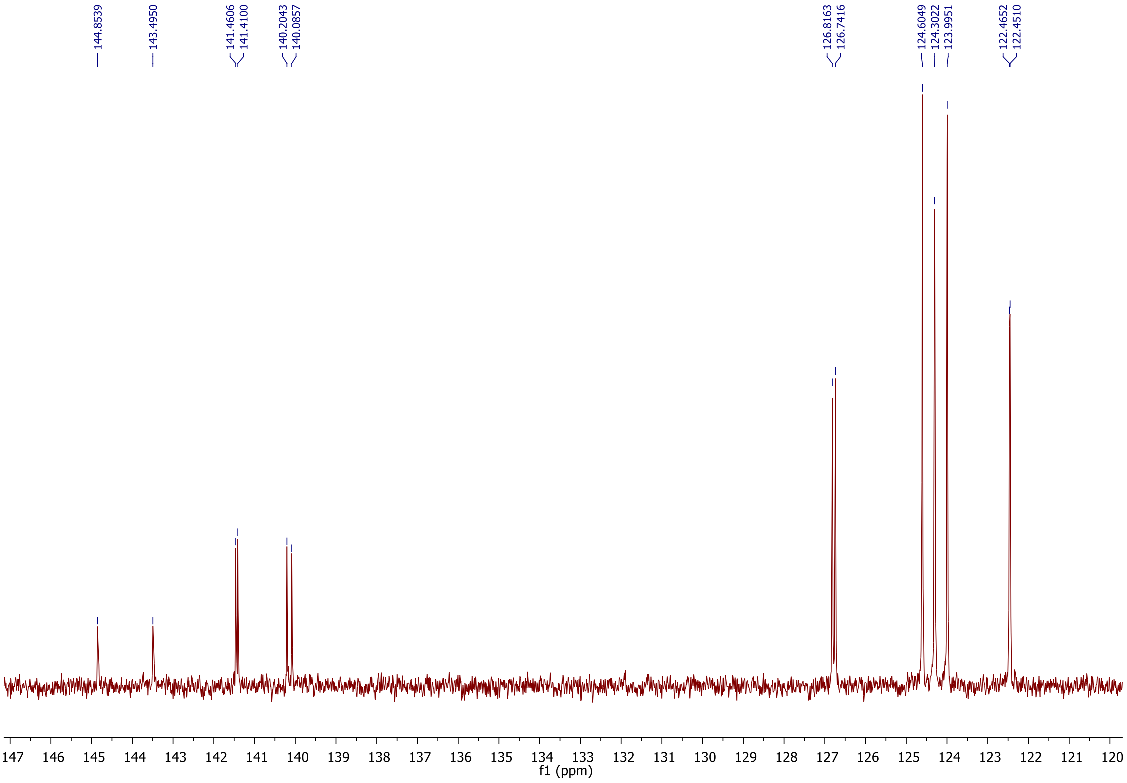


Figure S11: The [13] C NMR spectra of the compound **8**

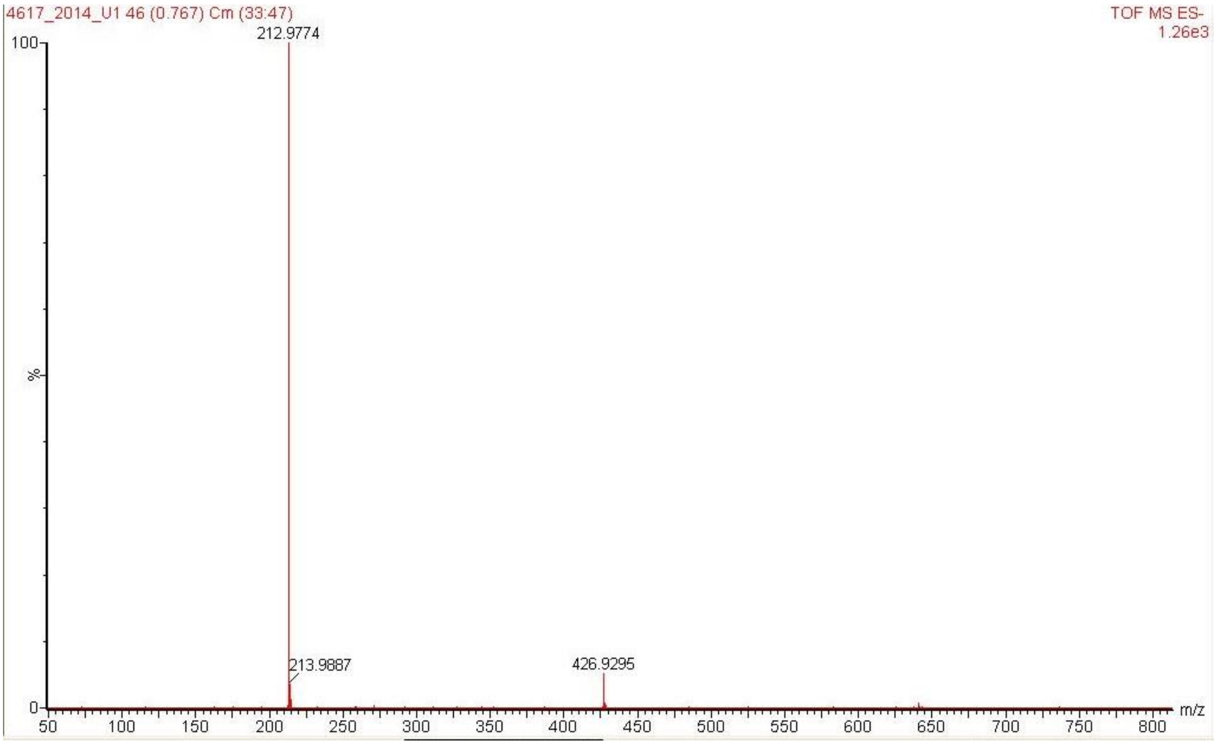


Figure S12: The HRMS spectra of the compound **8**

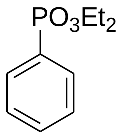


**21**

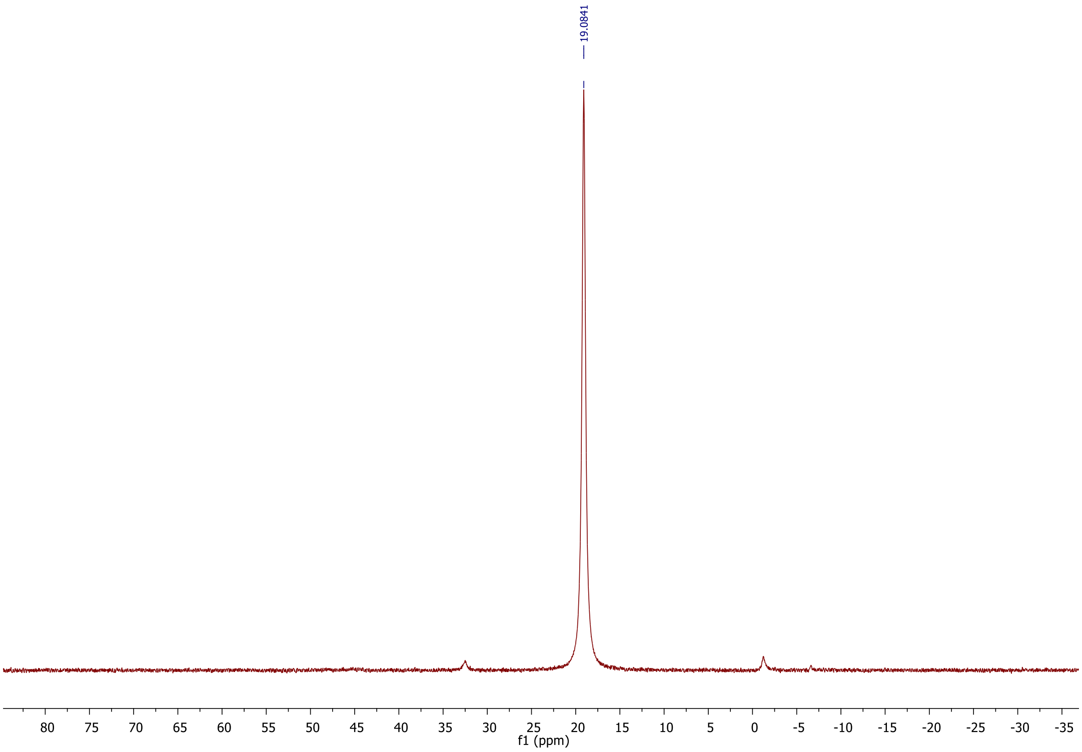


Figure S13: The $^{31}$P NMR spectra of the compound **21**

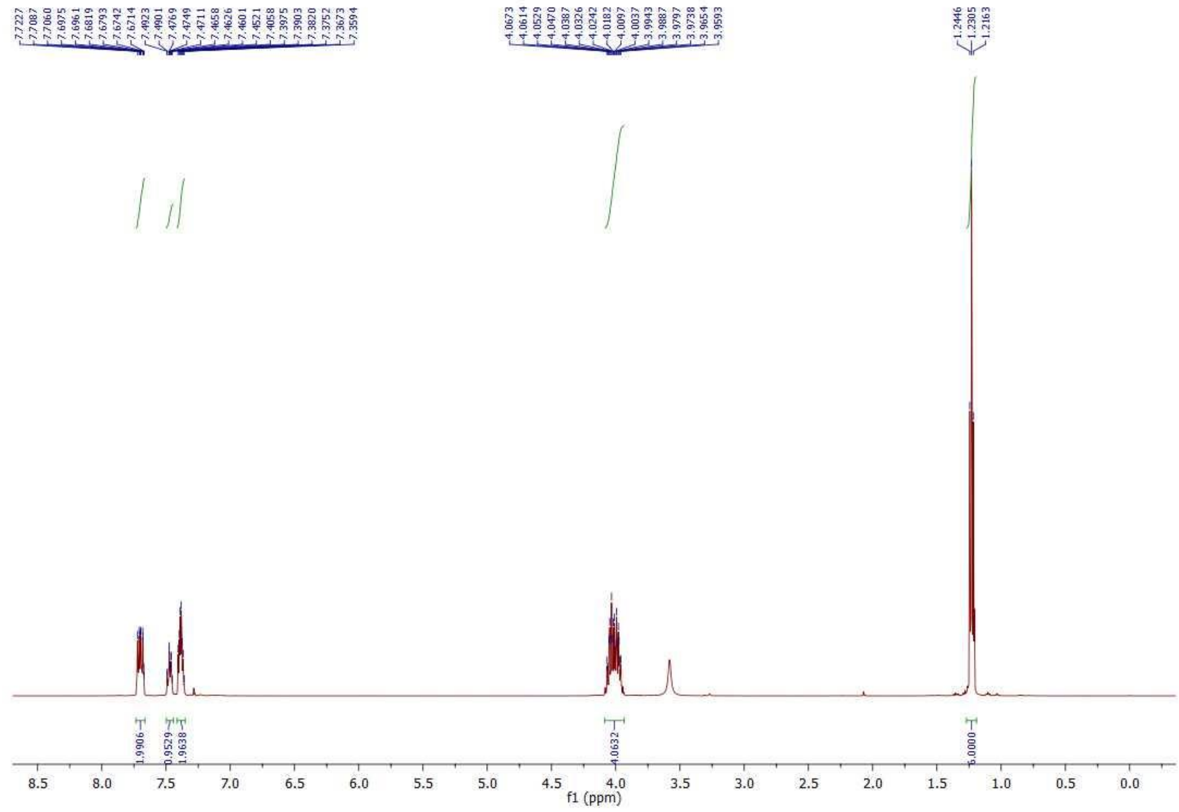


Figure S14: The [1] H NMR spectra of the compound **21**

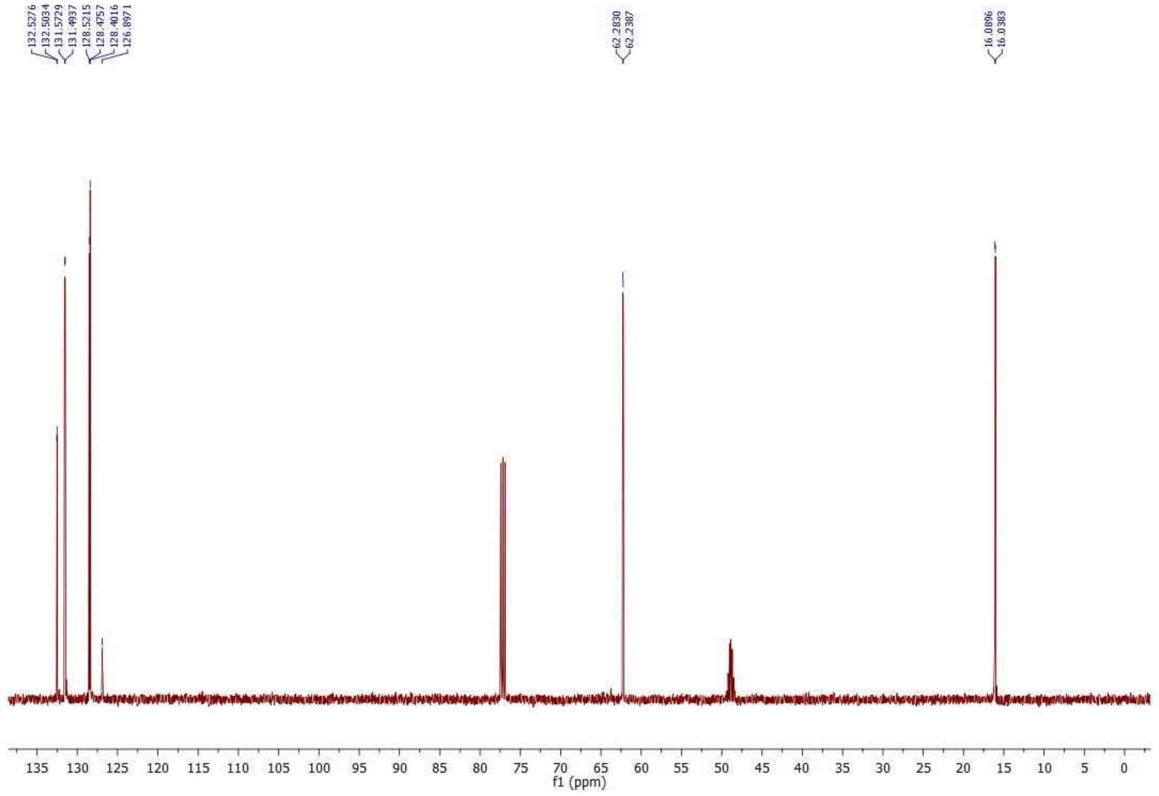


Figure S15: The [13] C NMR spectra of the compound **21**

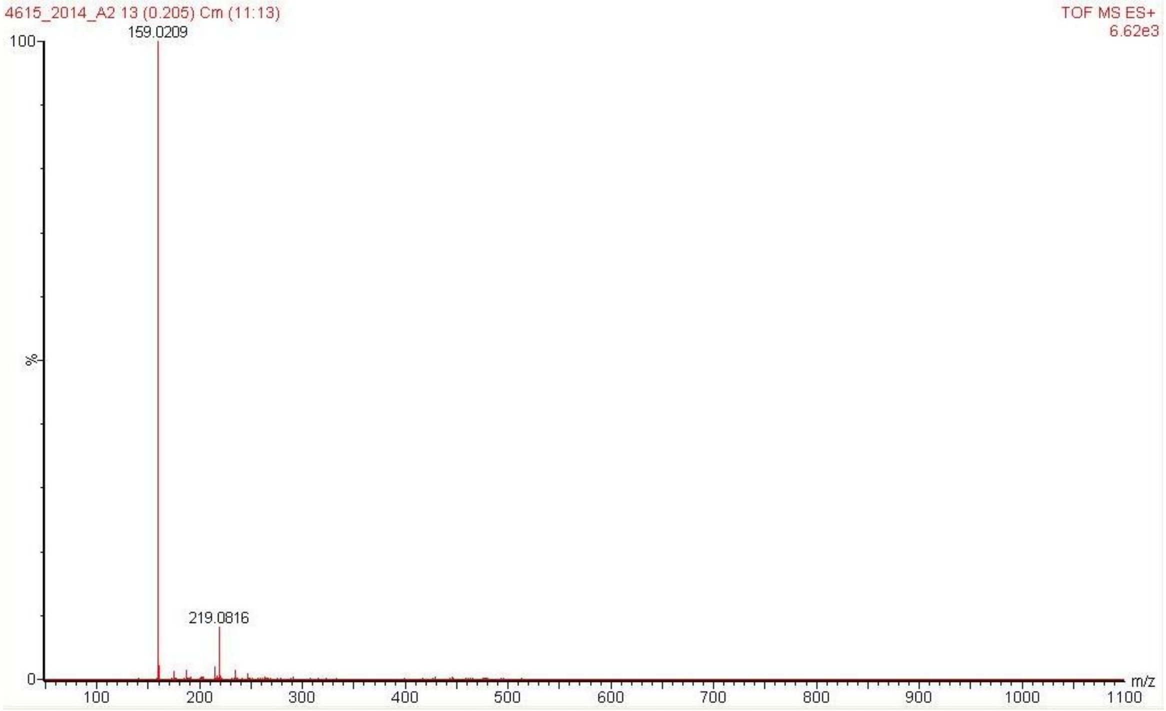


Figure S16: The HRMS spectra of the compound **21**

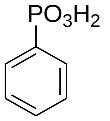


**24**

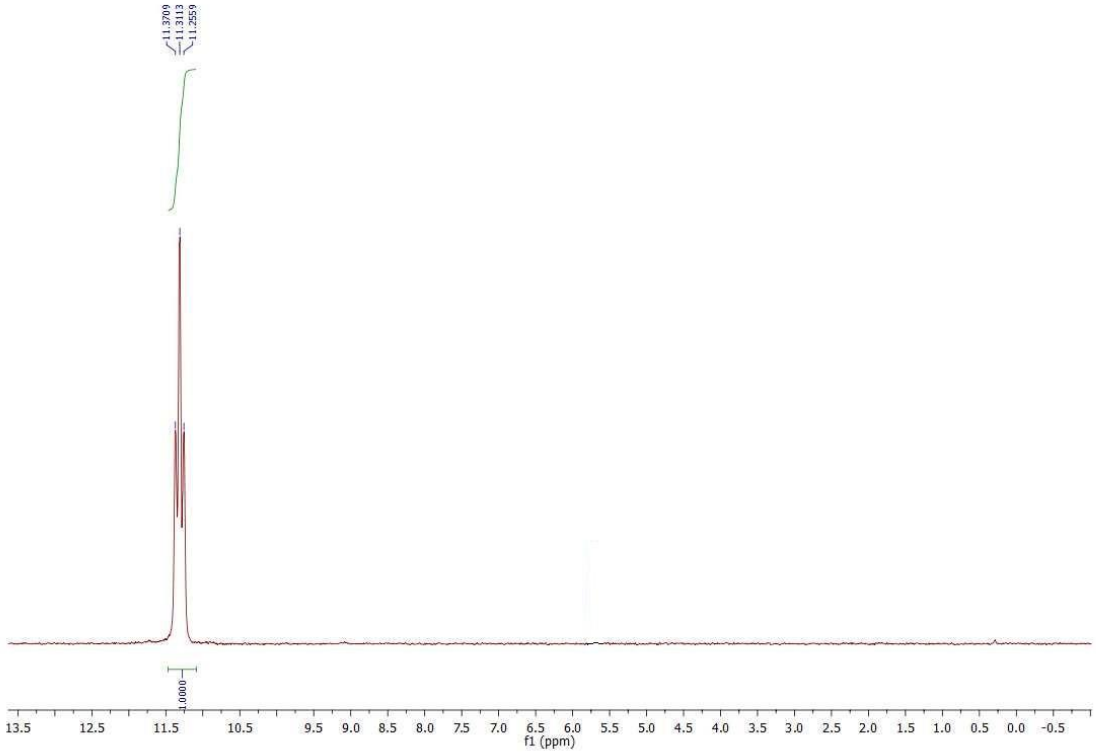


Figure S17: The $^{31}$ P NMR spectra of the compound **24**

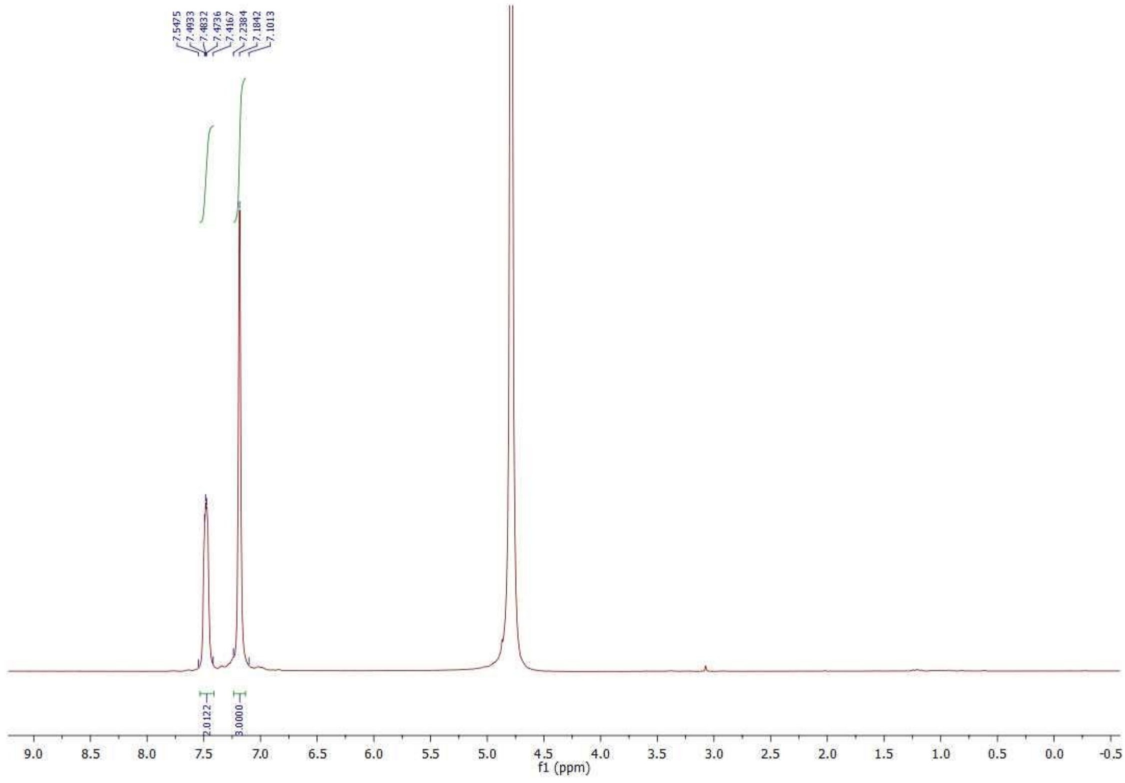


Figure S18: The [1] H NMR spectra of the compound **24**

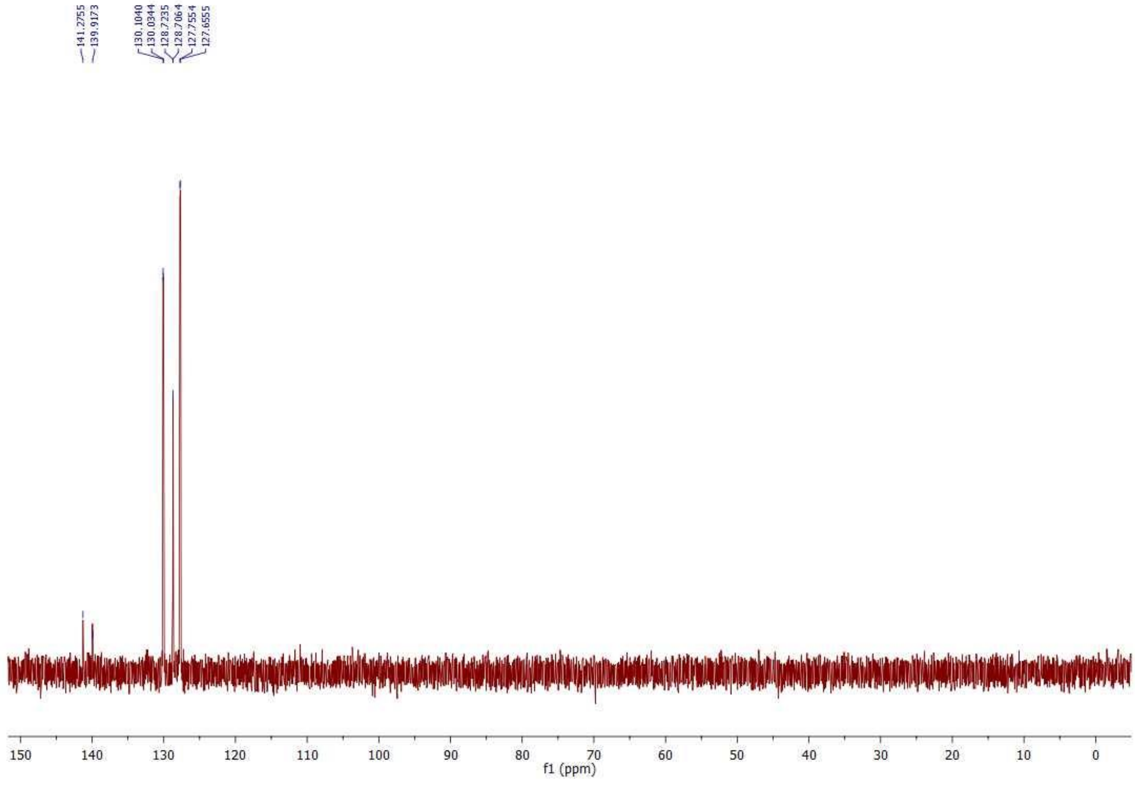


Figure S19: The [13] C NMR spectra of the compound **24**

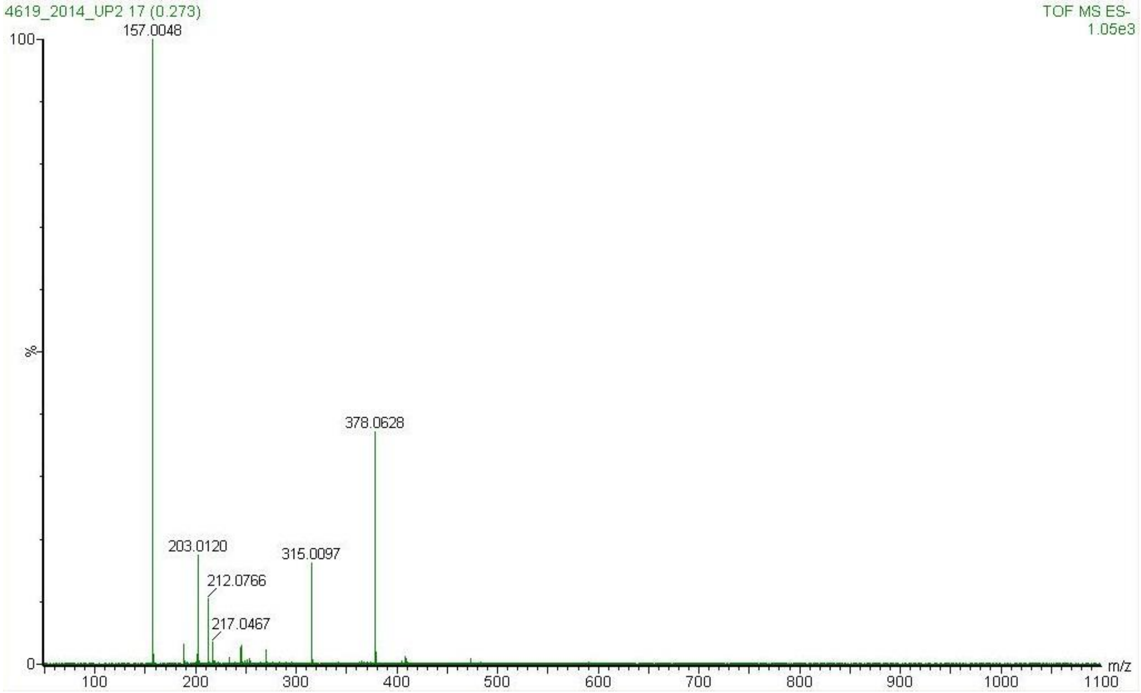


Figure S20: The HRMS spectra of the compound **24**

**22** 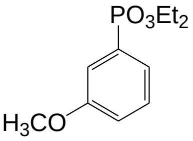


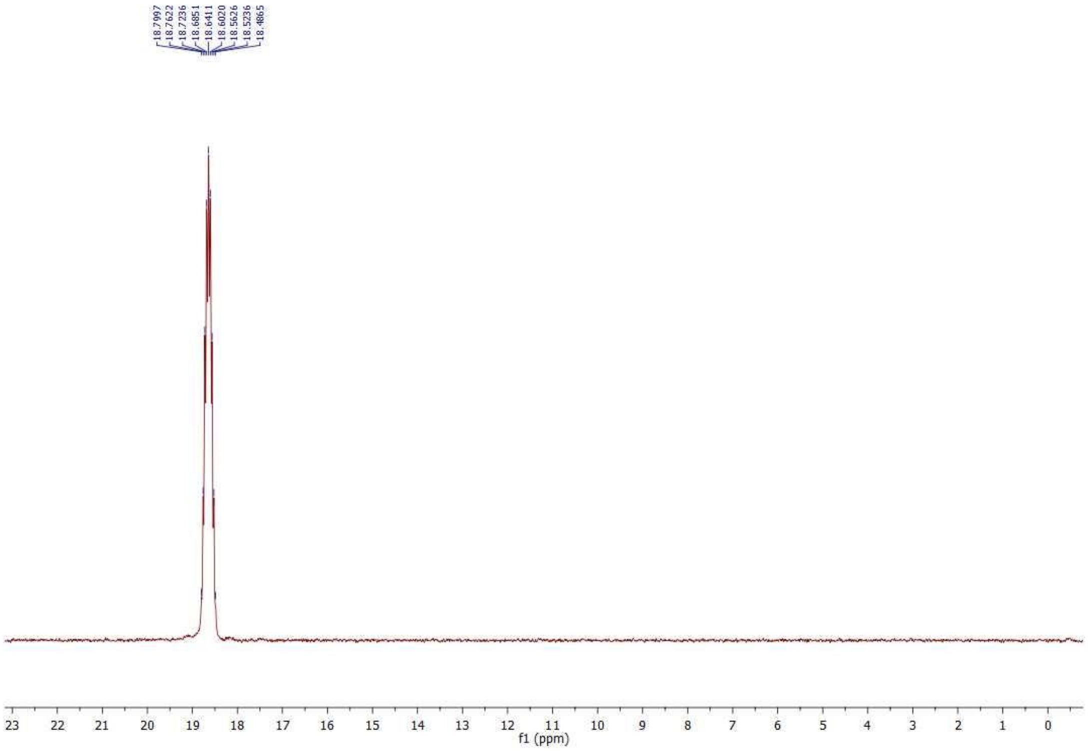


Figure S21: The $^{31}$ P NMR spectra of the compound **22**

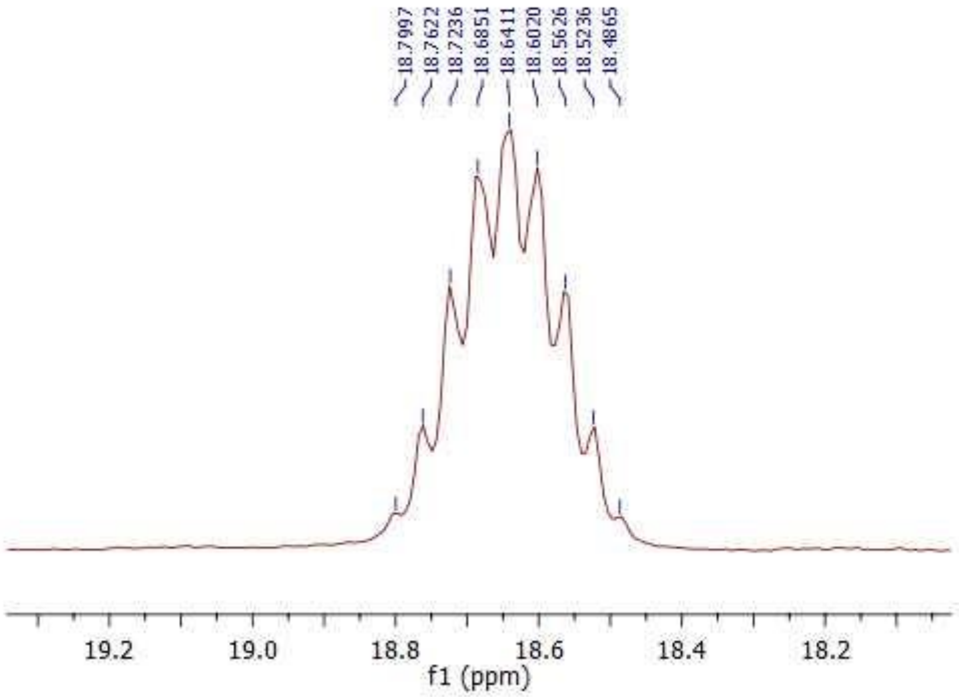


Figure S22: The [31] P NMR spectra of the compound **22**

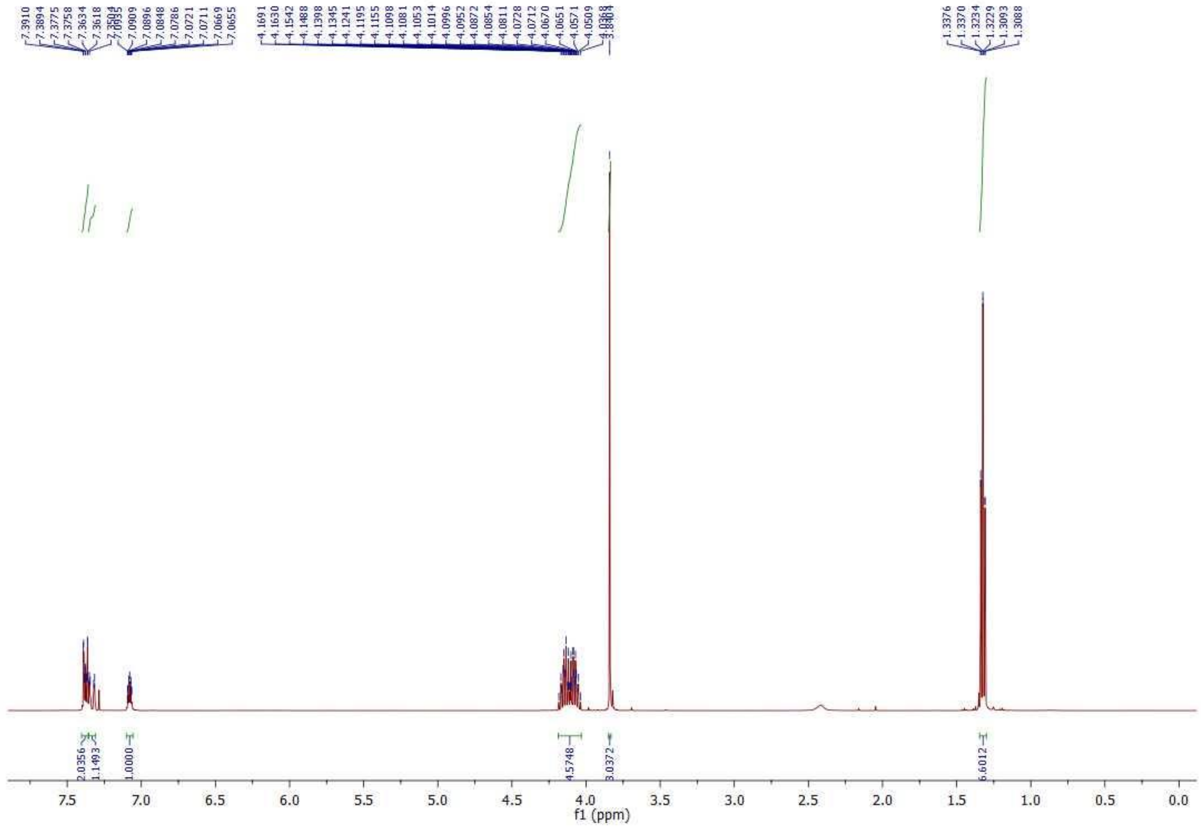


Figure S23: The [1] H NMR spectra of the compound **22**

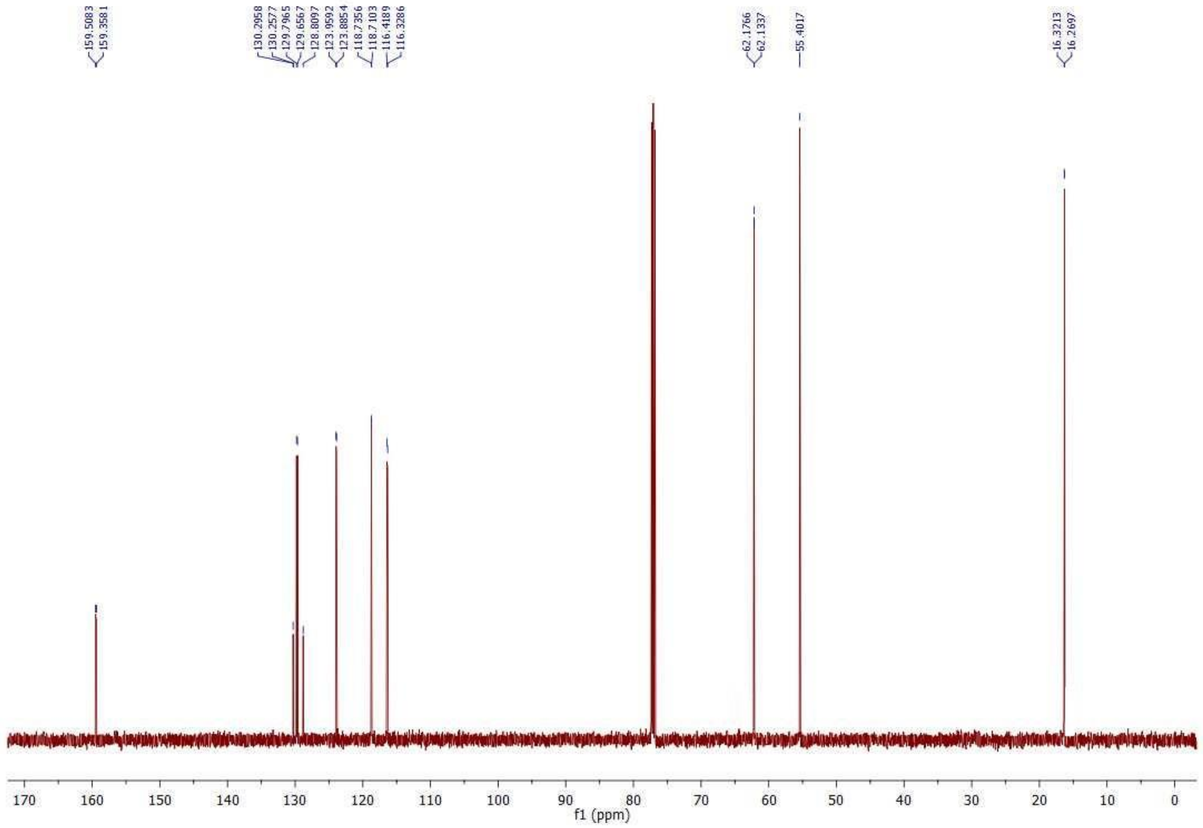


Figure S24: The [13] C NMR spectra of the compound **22**

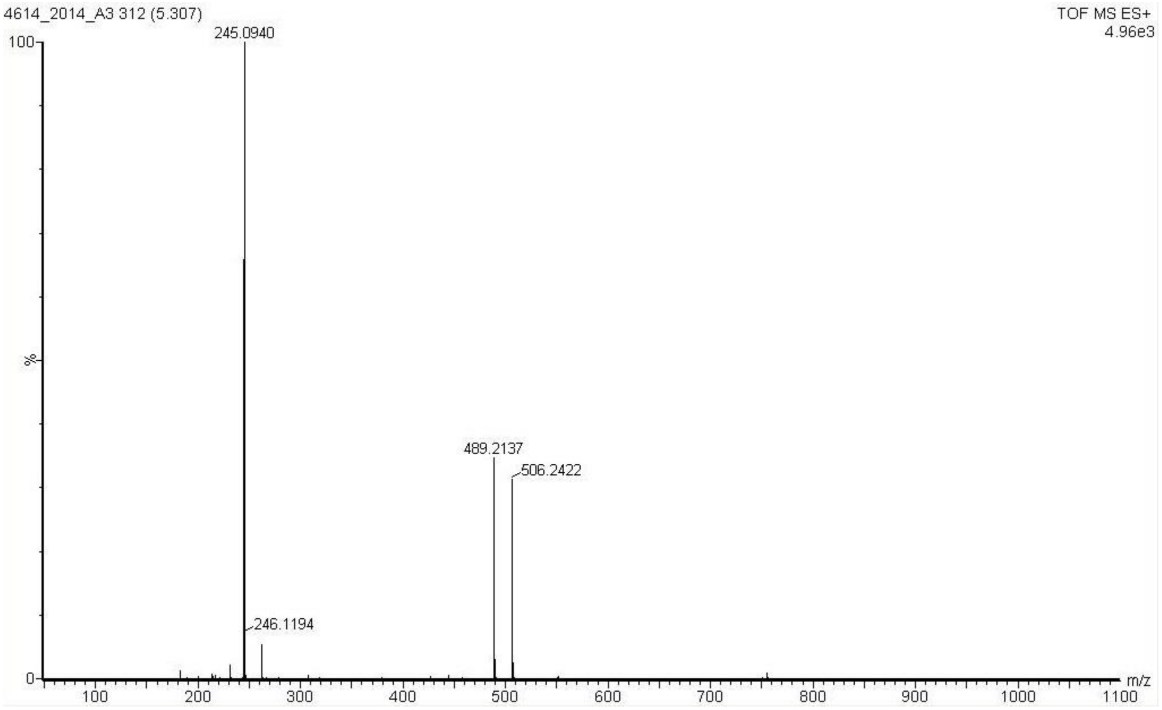


Figure S25: The HRMS spectra of the compound **22**

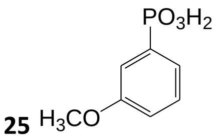

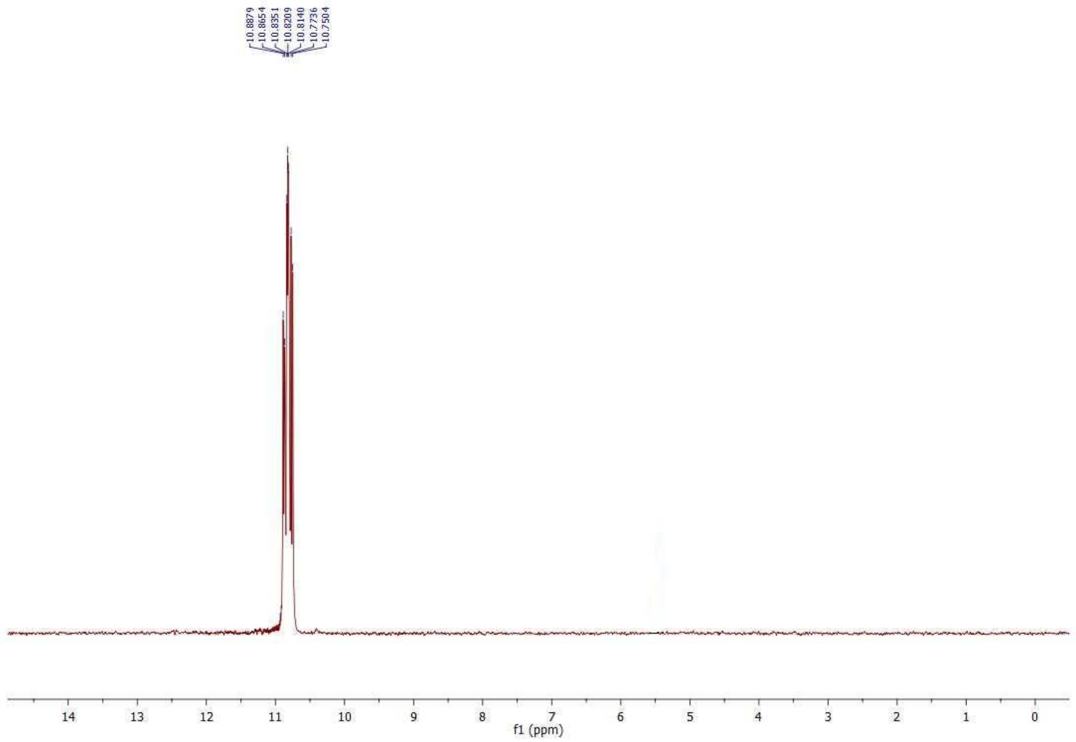

Figure S26: The [31] P NMR spectra of the compound **25**

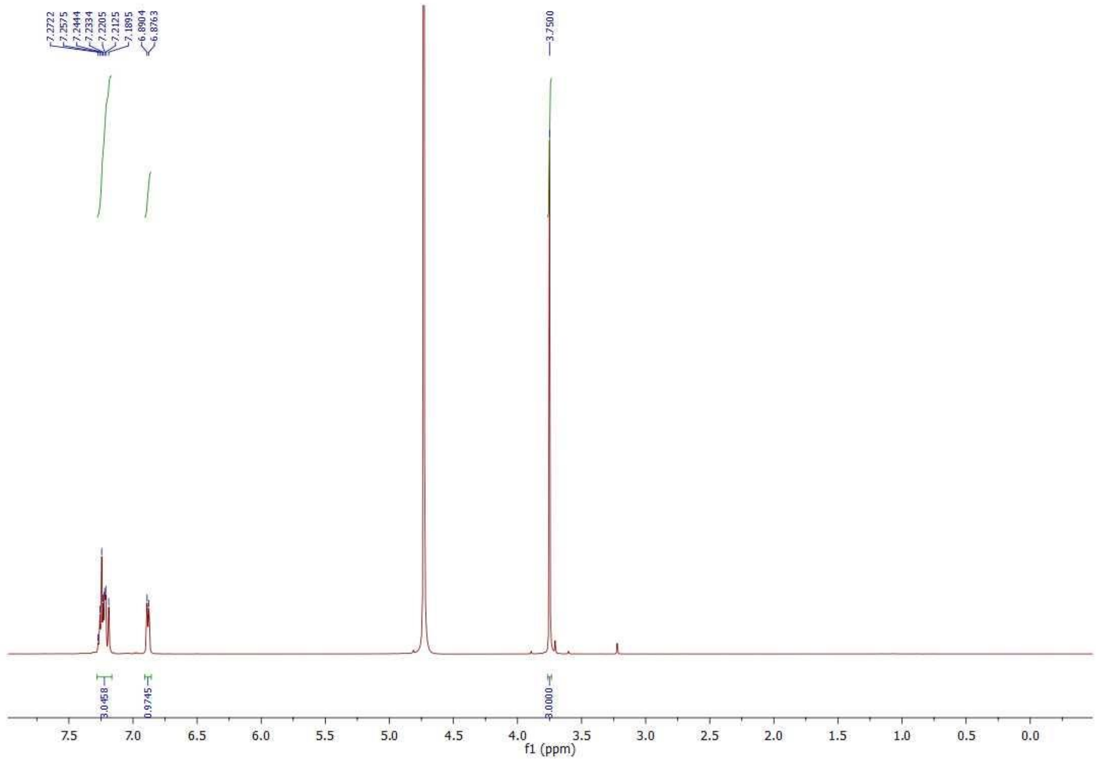

Figure S27: The [1] H NMR spectra of the compound **25**

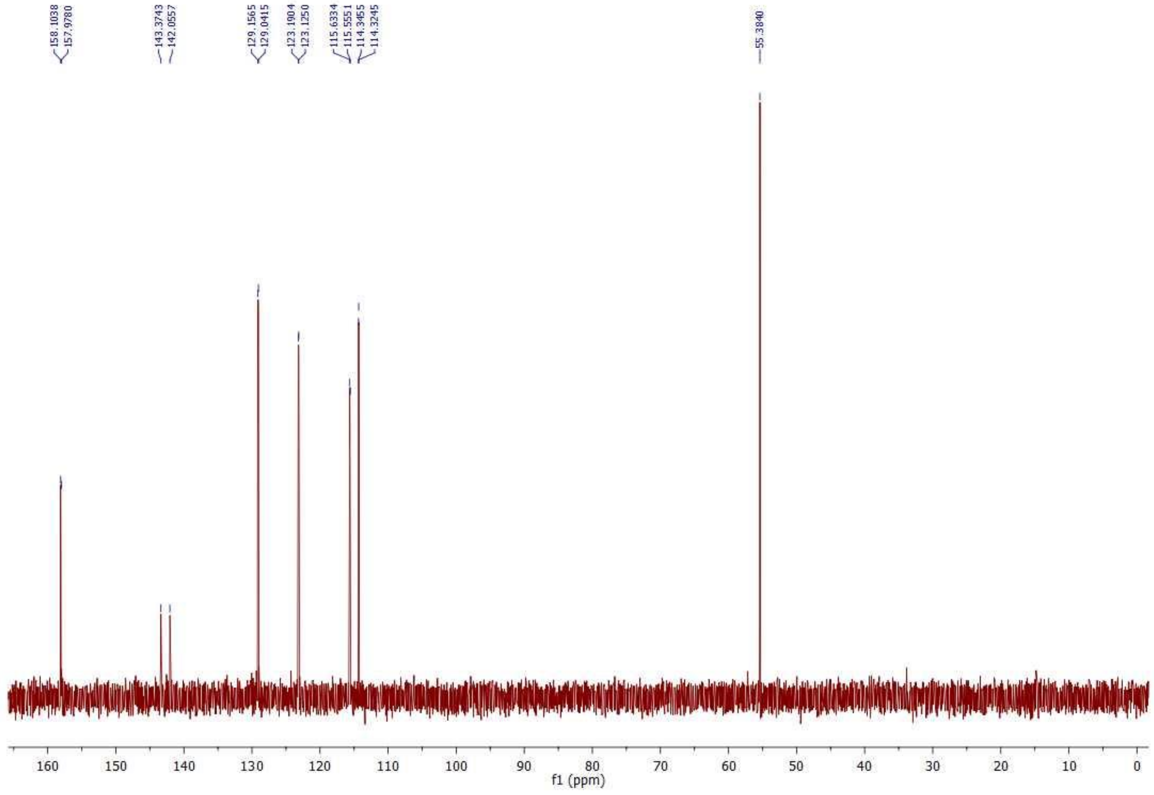


Figure S28: The [13] C NMR spectra of the compound **25**

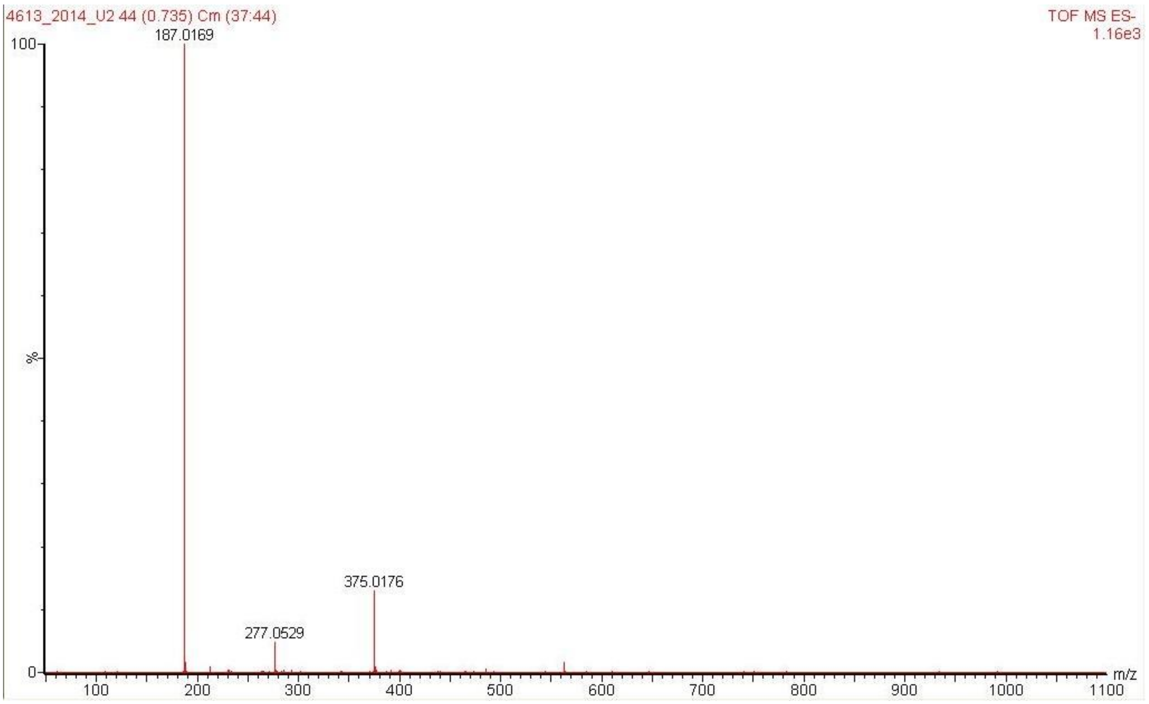


Figure S29: The HRMS spectra of the compound **25**

26 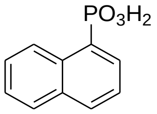


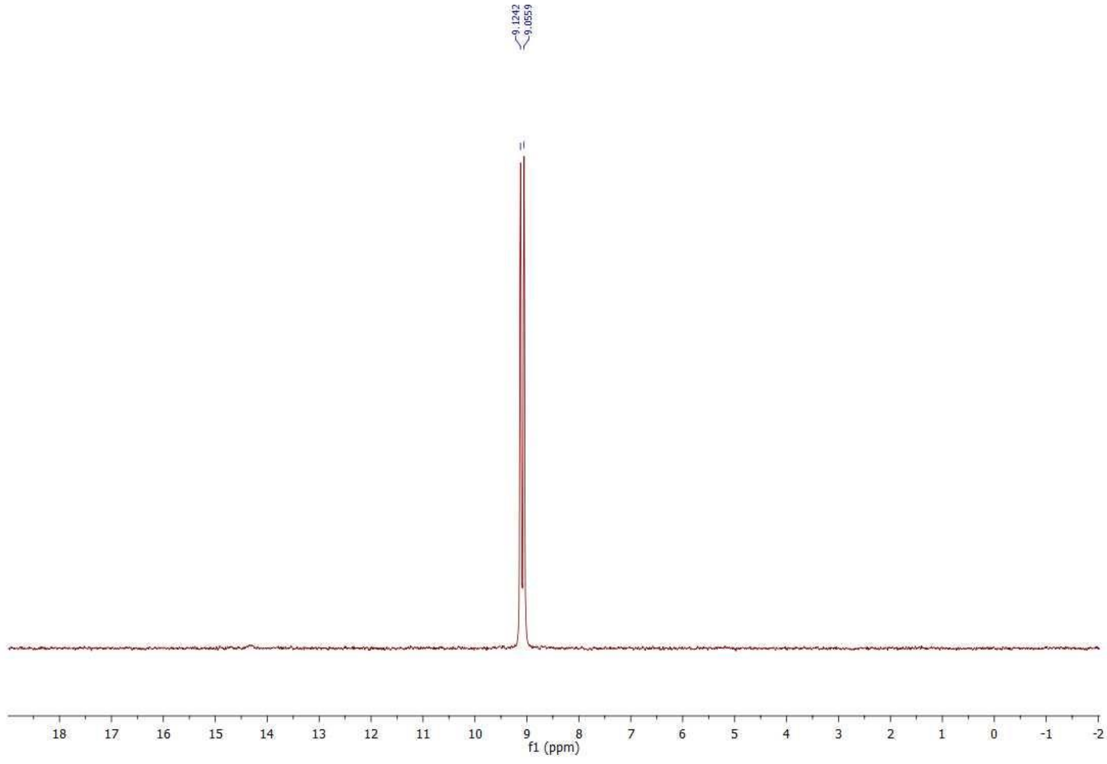


Figure S30: The [31] P NMR spectra of the compound **26**

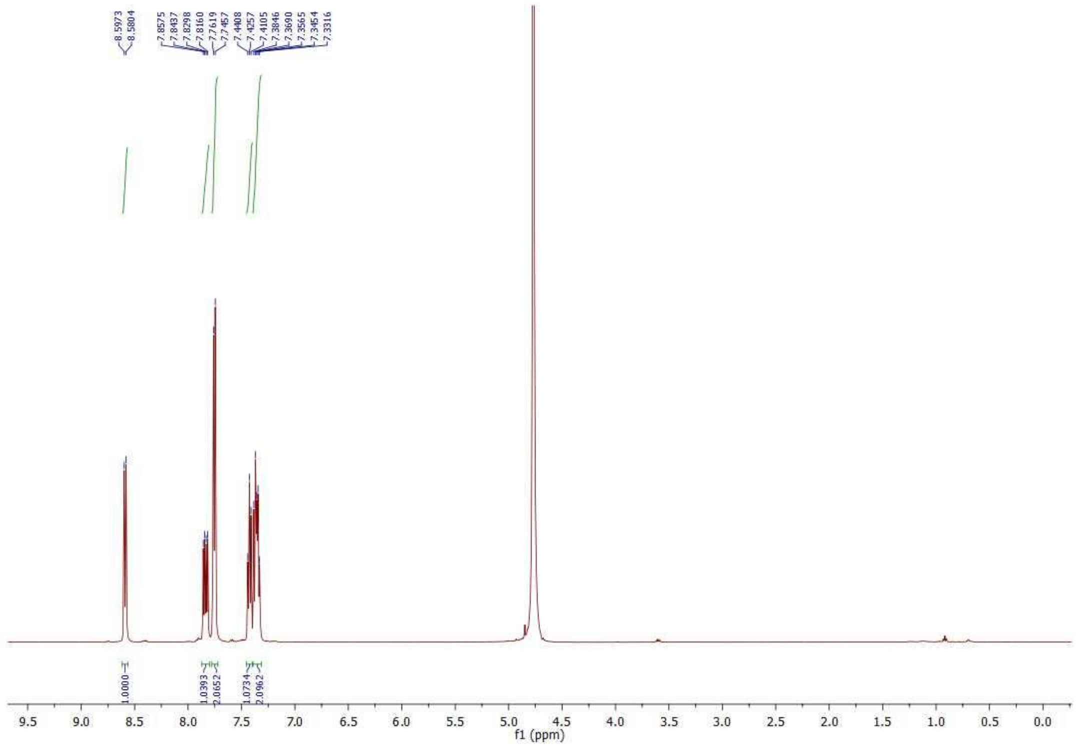


Figure S31: The [1] H NMR spectra of the compound **26**

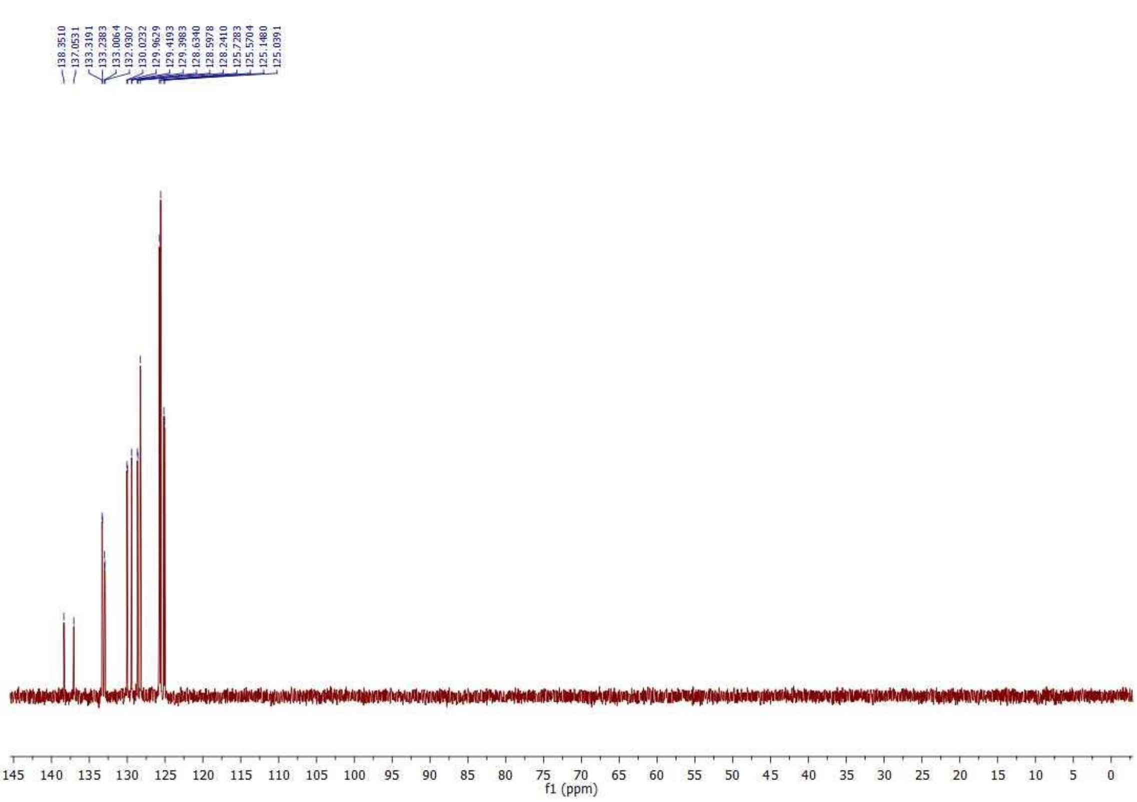


Figure S32: The [13] C NMR spectra of the compound **26**

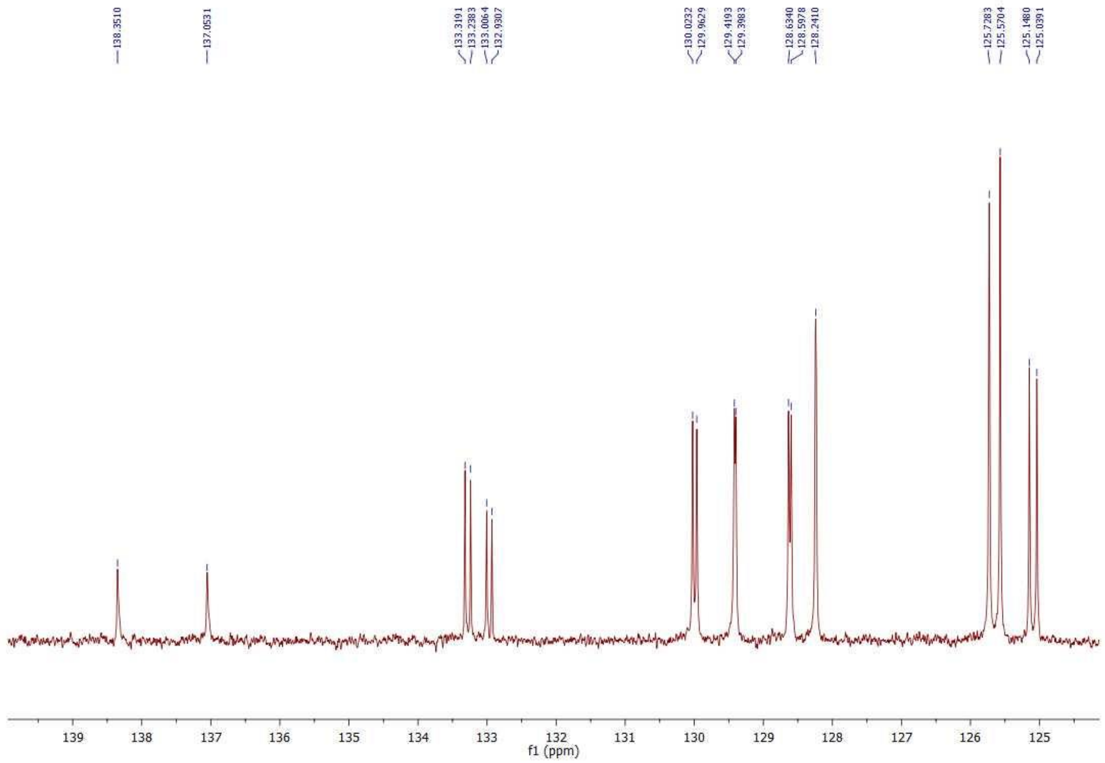


Figure S33: The [13] C NMR spectra of the compound **26**

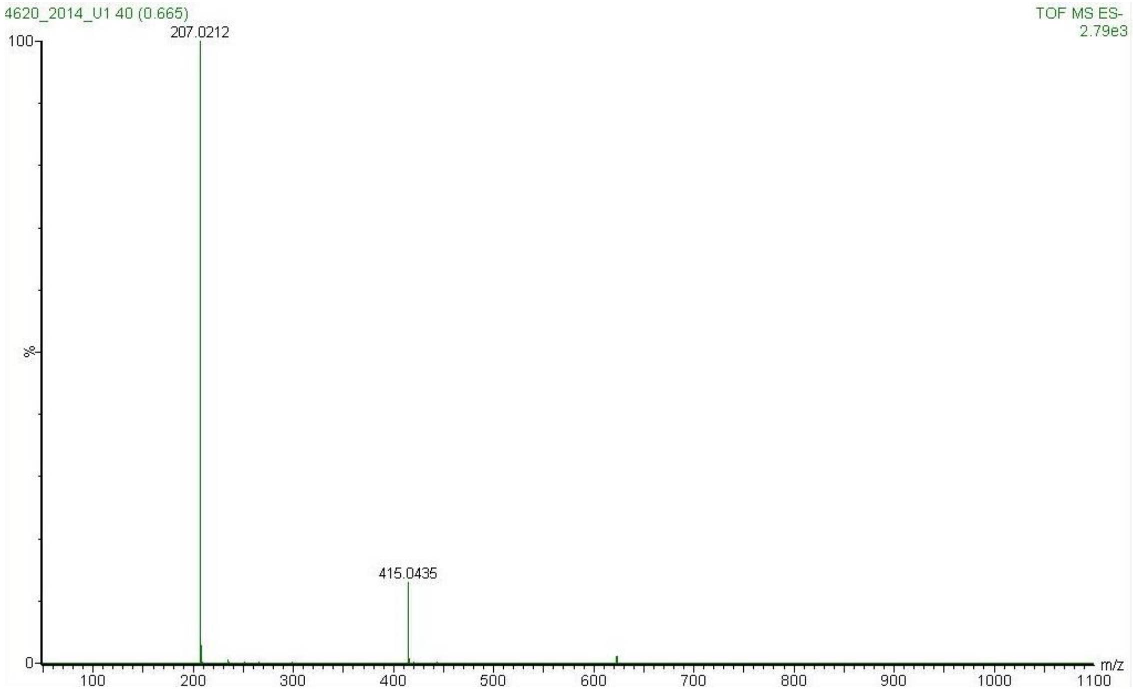


Figure S34: The HRMS spectra of the compound **26**

**7** 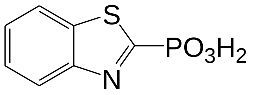


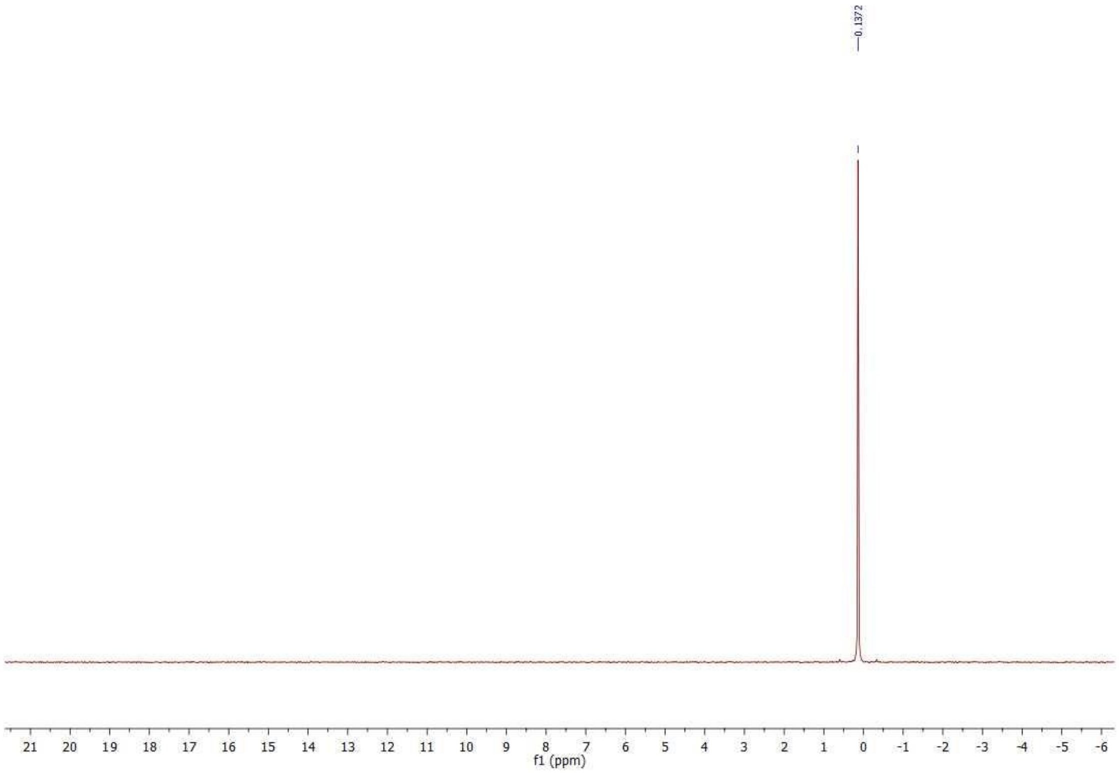


Figure S35: The $^{31}$ P NMR spectra of the compound **7**

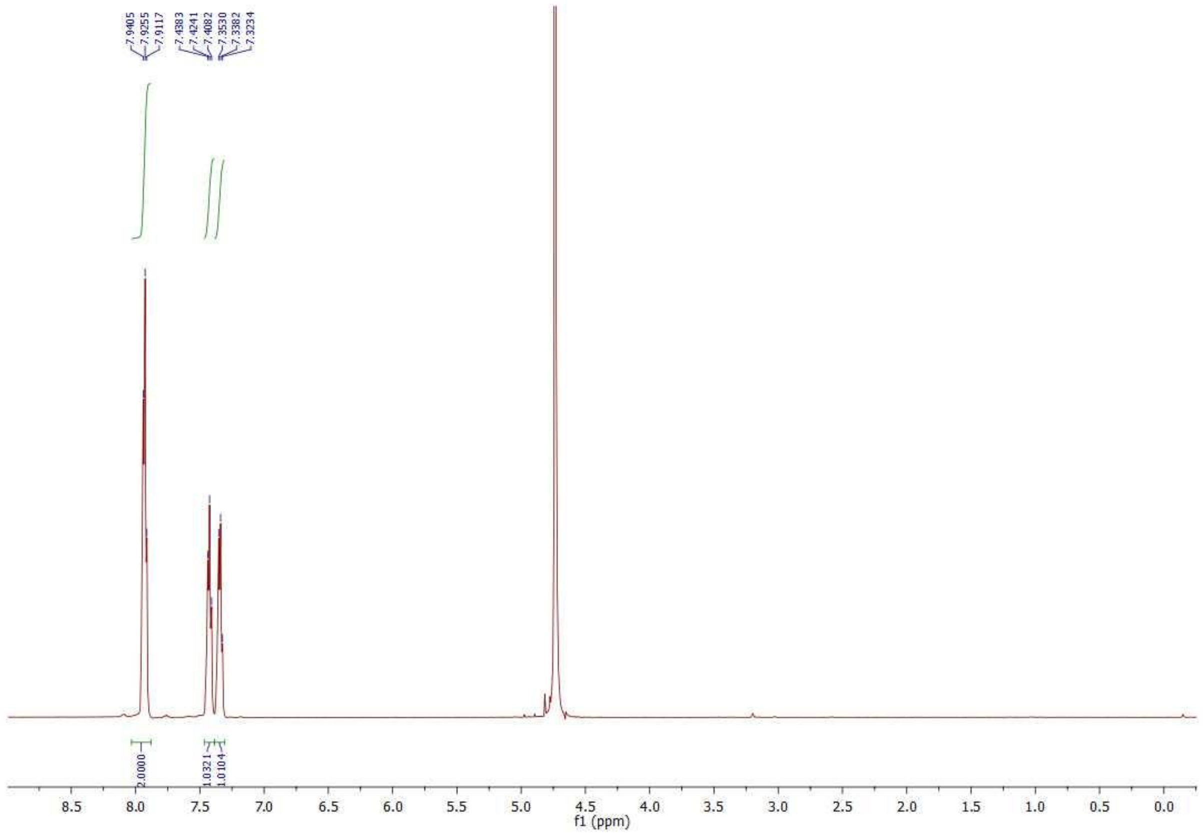


Figure S36: The [1] H NMR spectra of the compound **7**

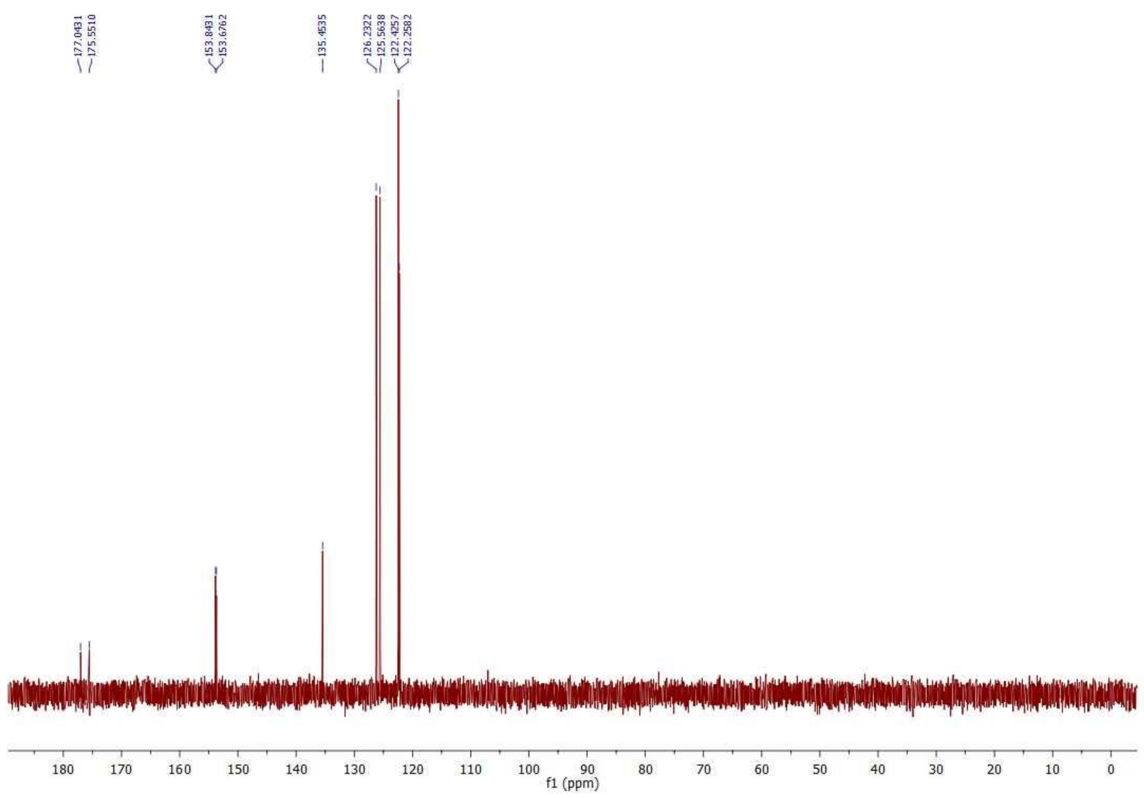


Figure S37: The [13] C NMR spectra of the compound **7**

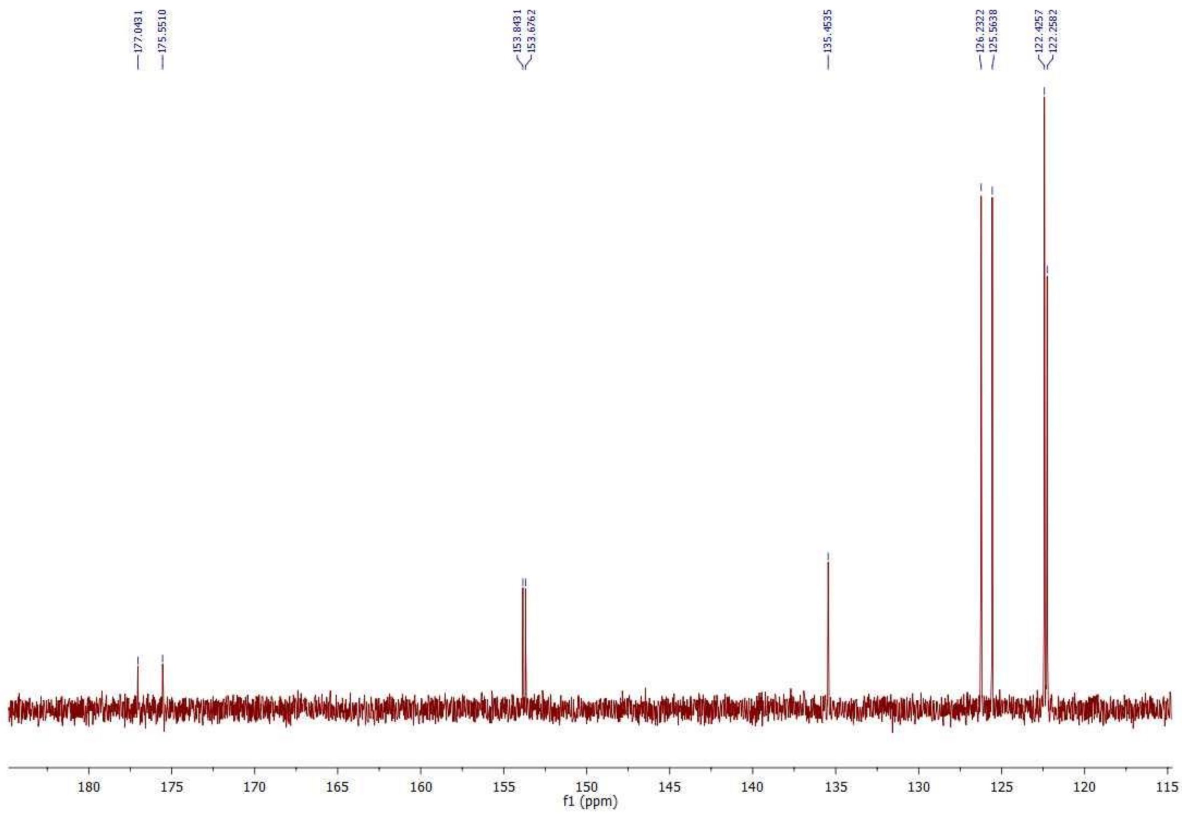


Figure S38: The [13] C NMR spectra of the compound **7**

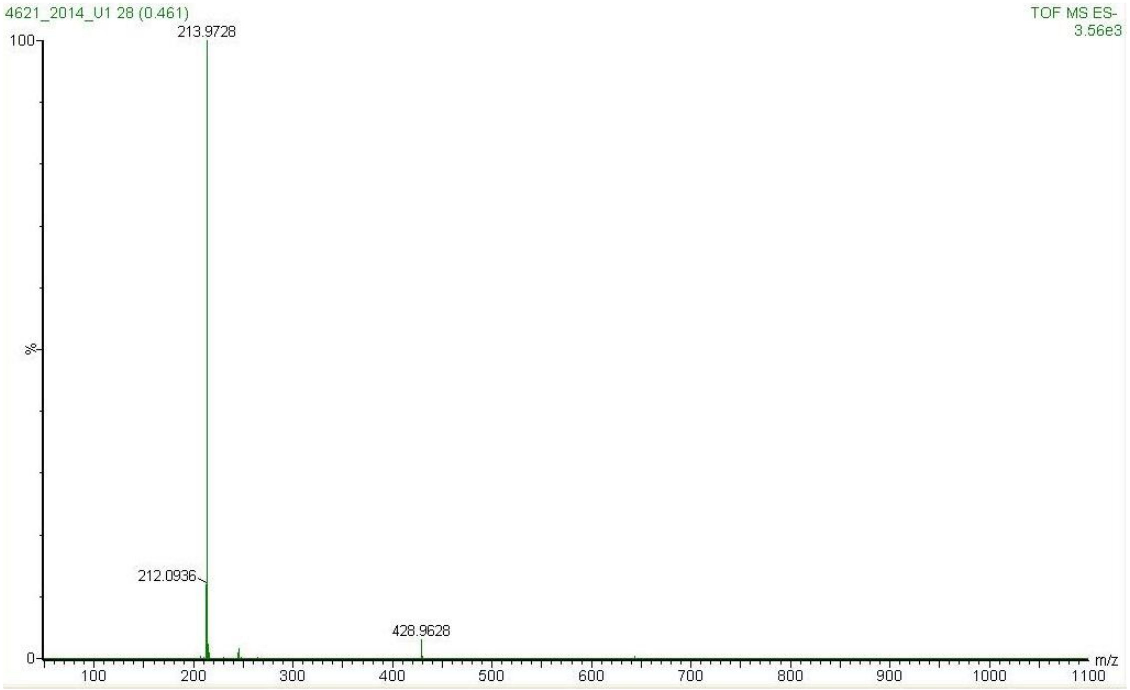


Figure S39: The HRMS spectra of the compound **7**

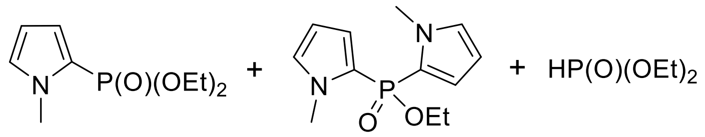


**18** **19** **20**

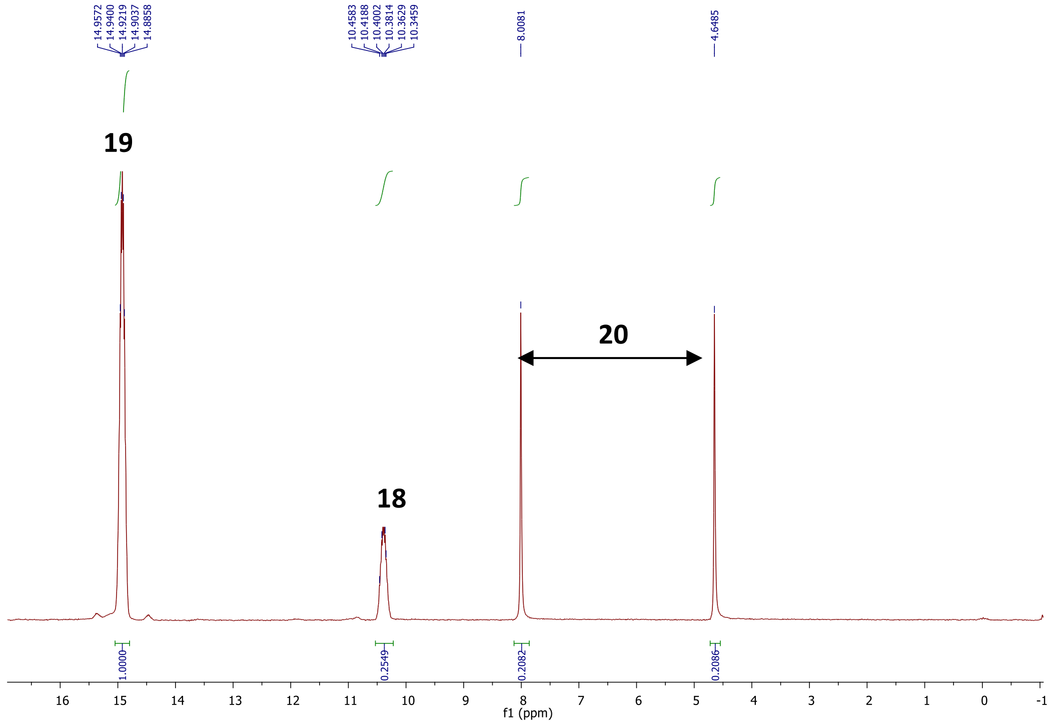


Figure S40: The $^{31}$ P NMR spectra of the compounds **18**, **19** and **20**

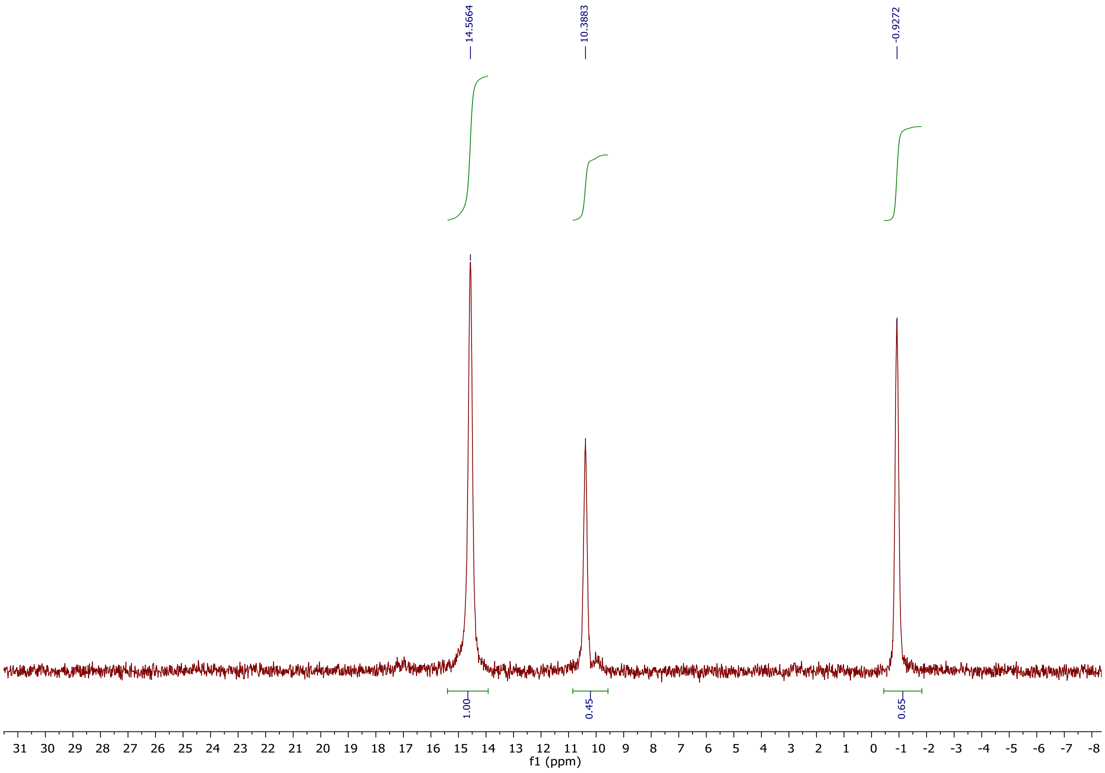

Figure S41: The $^{31}$ P{$^{1}$H} NMR spectra of the compounds **18**, **19** and **20**

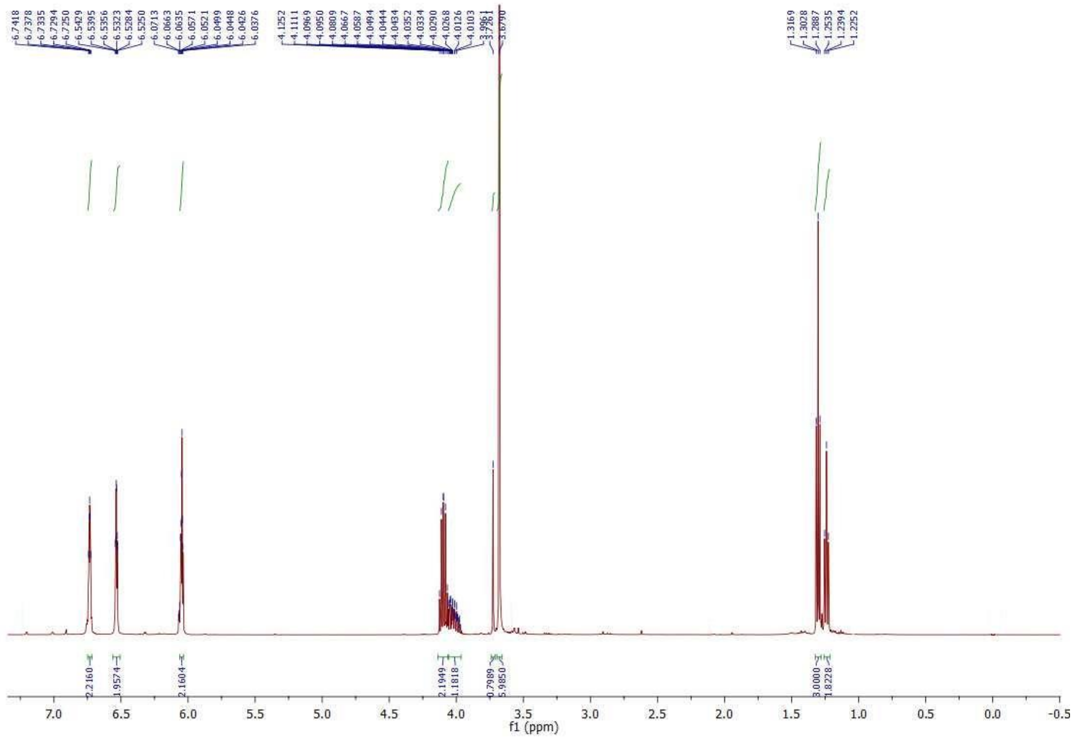

Figure S42: The $^{1}$ H NMR spectra of the compounds **18**, **19** and **20**

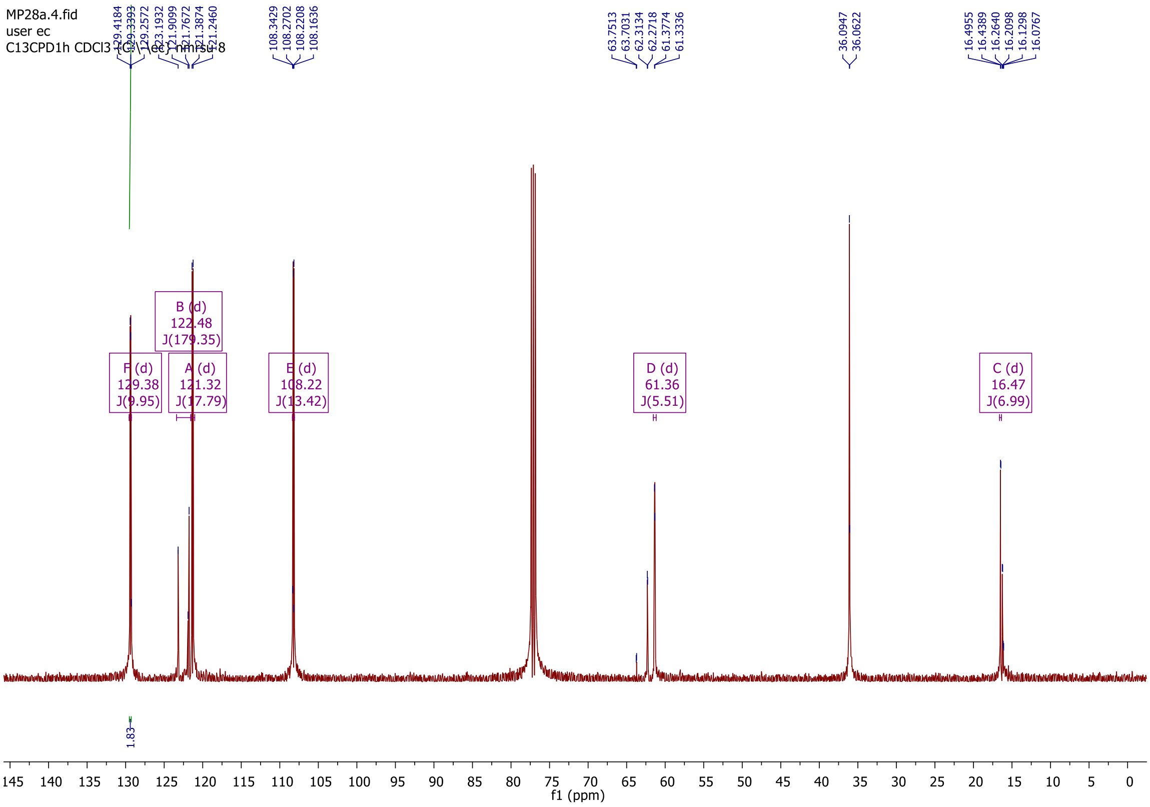


Figure S43: The [1] H NMR spectra of the compounds **18**, **19** and **20**

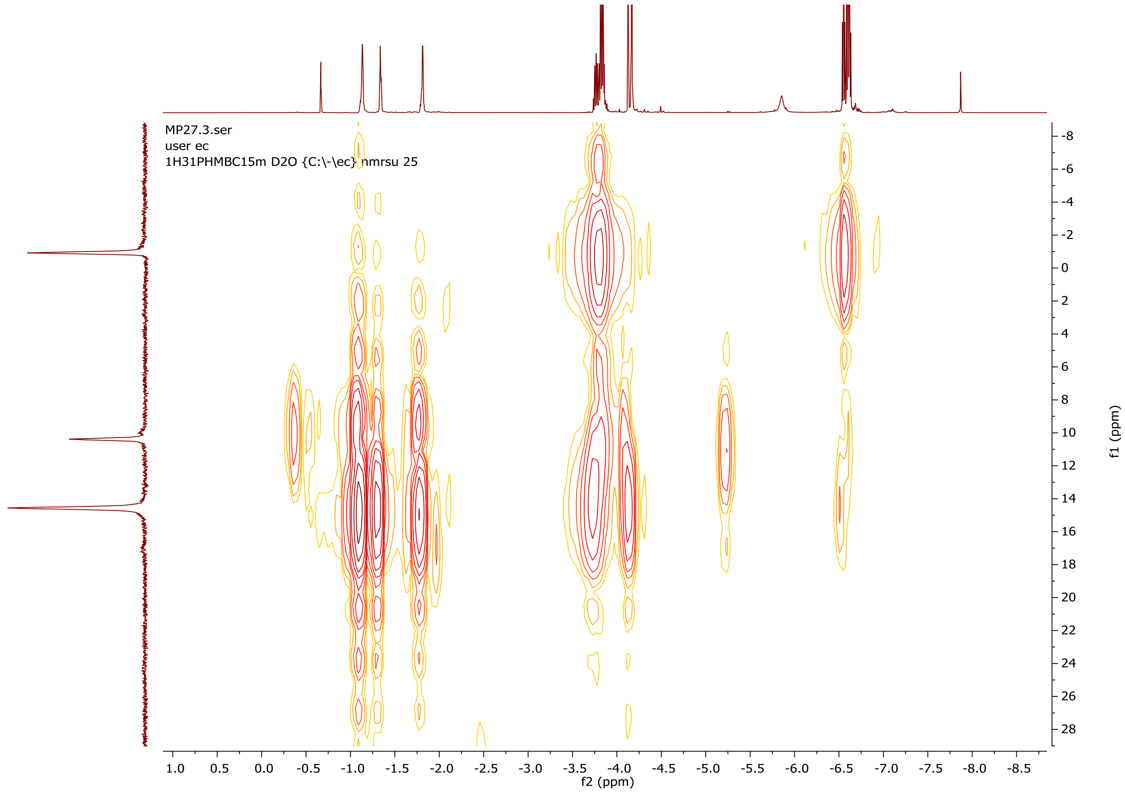


Figure S44: The [1] H -[31] P NMR spectra of the compounds **18**, **19** and **20**

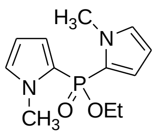

**19**

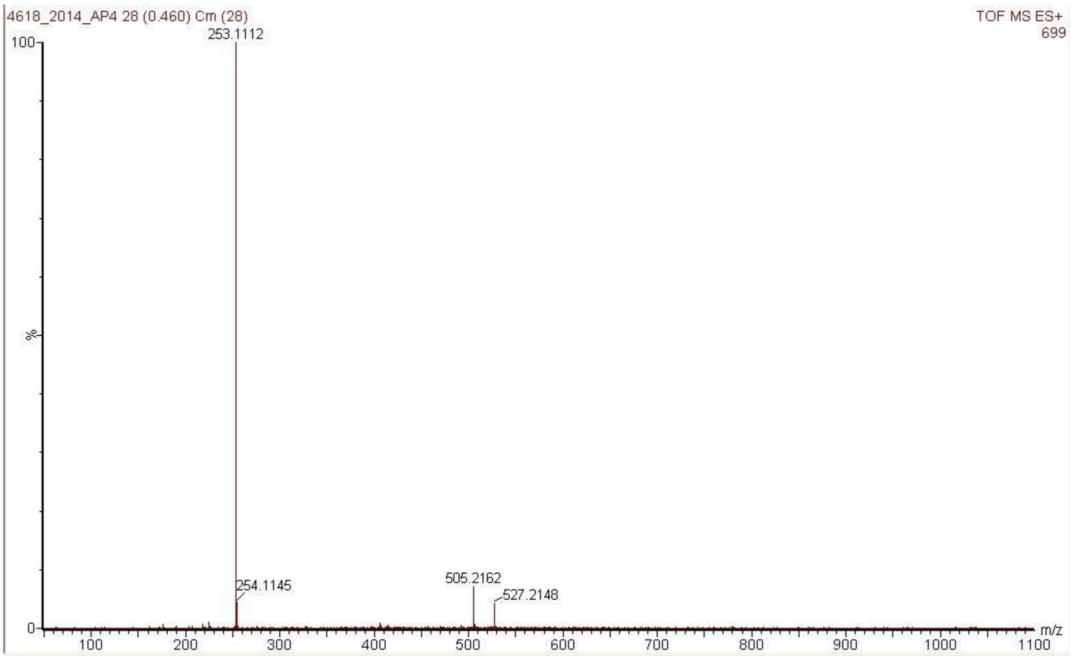


Figure S45: The HRMS spectra of the compound **19**

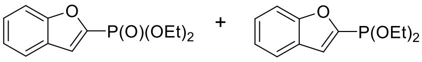

**10** **11**

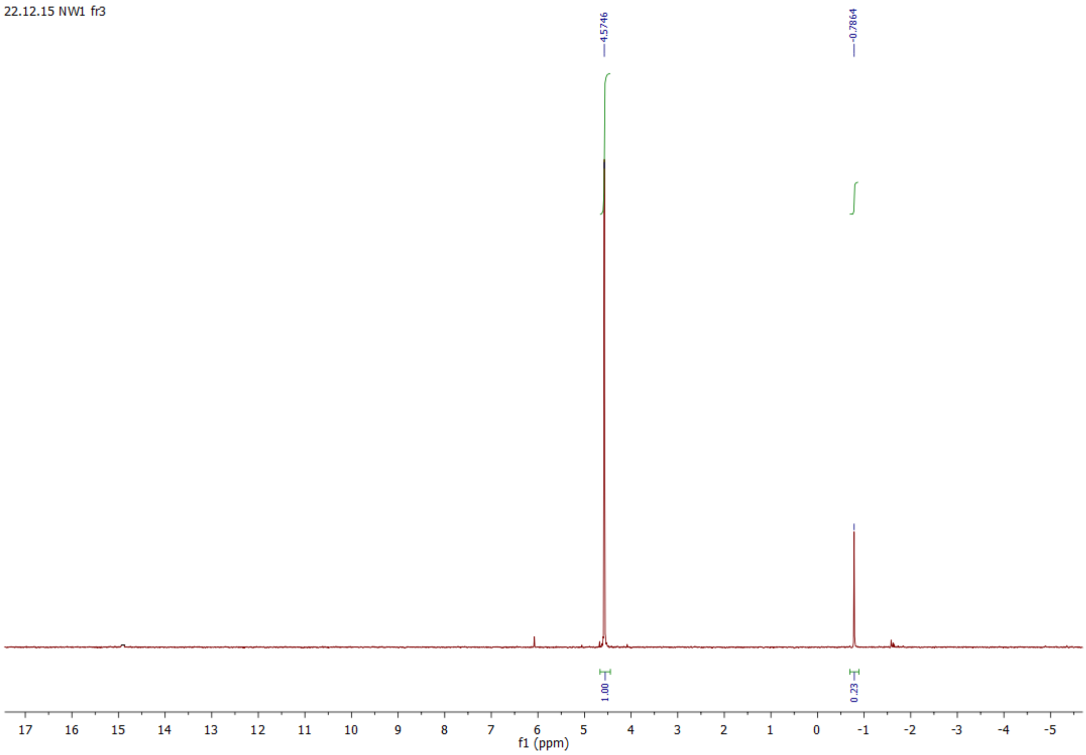


Figure S46: The $^{31}$ P NMR spectra of the compounds **10** and **11**

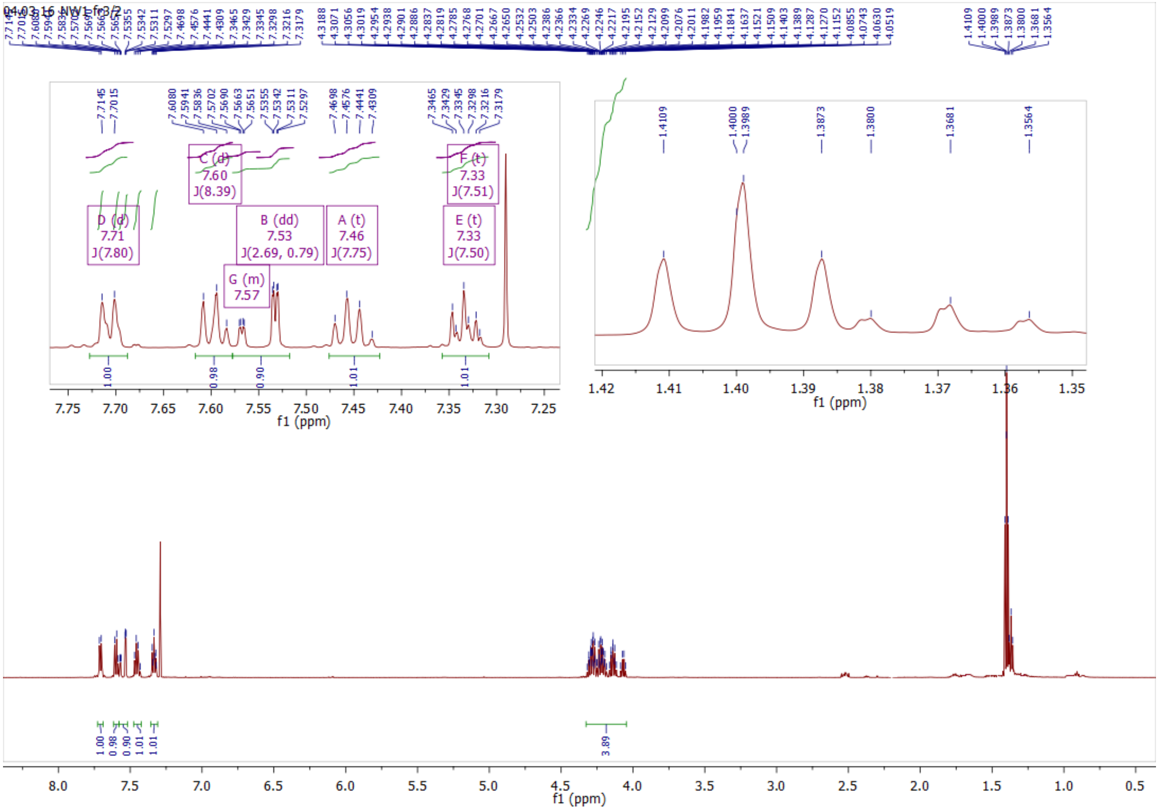

Figure S47: The [1] H NMR spectra of the compounds **10** and **11**

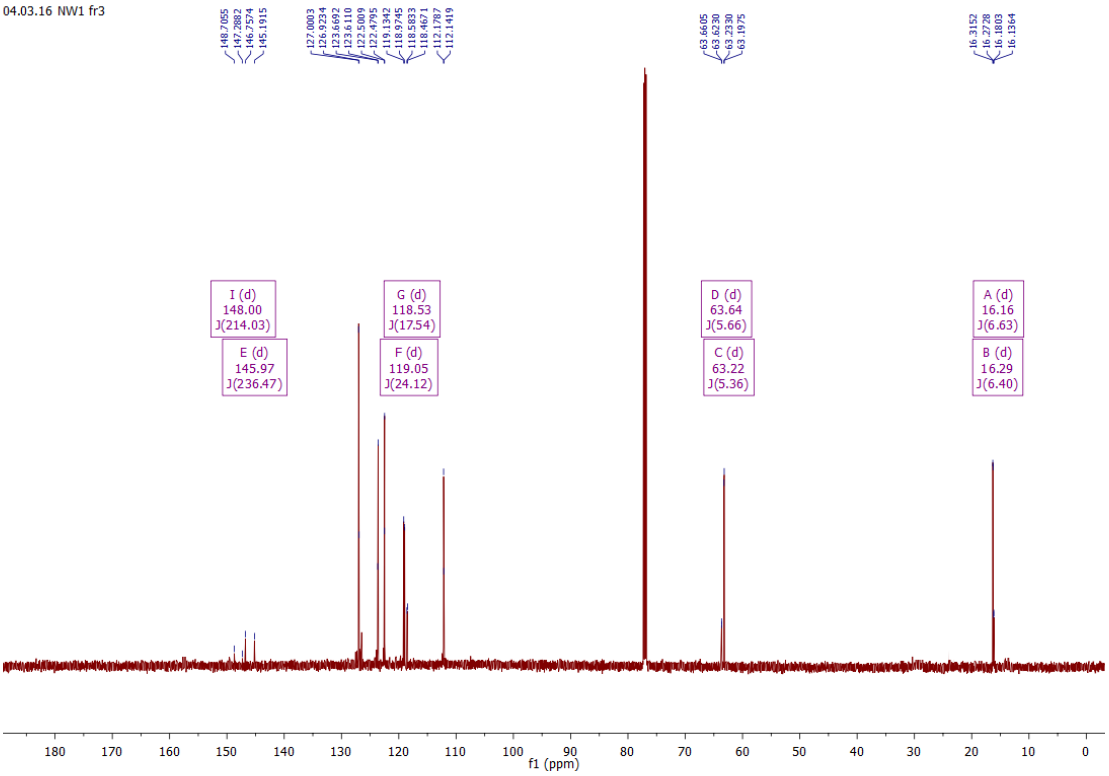

Figure S48: The [13] C NMR spectra of the compounds **10** and **11**

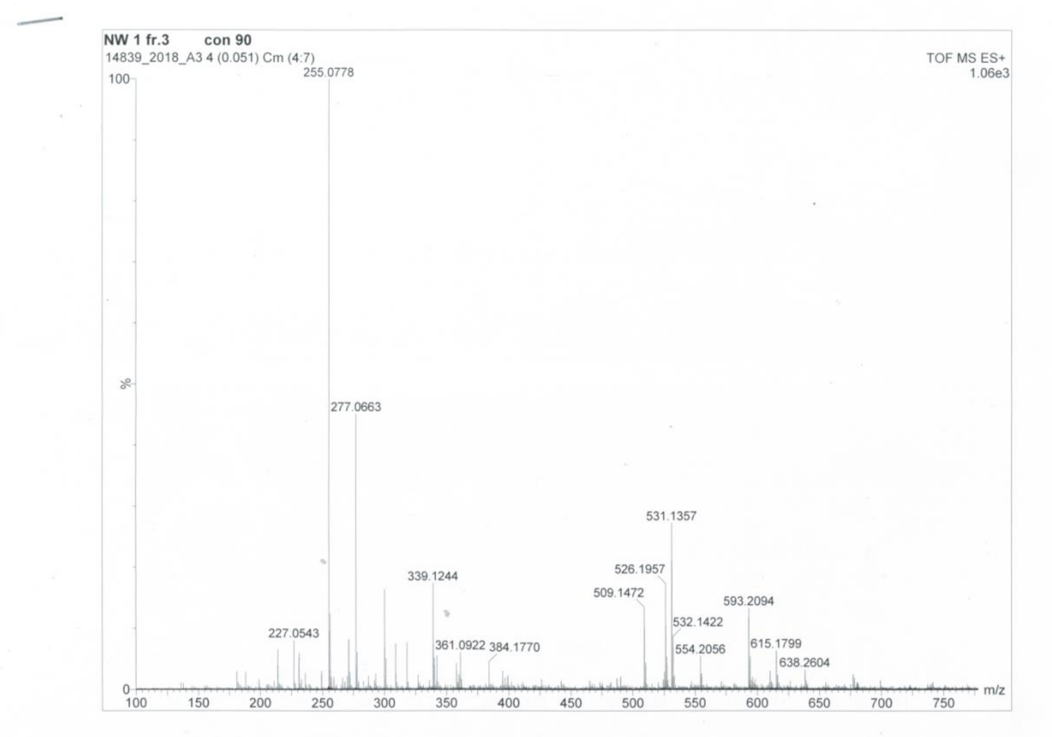


Figure S49: The HRMS spectra of the compounds **10** and **11**

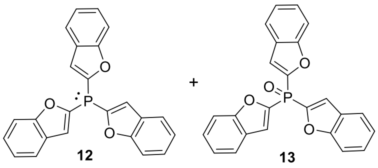


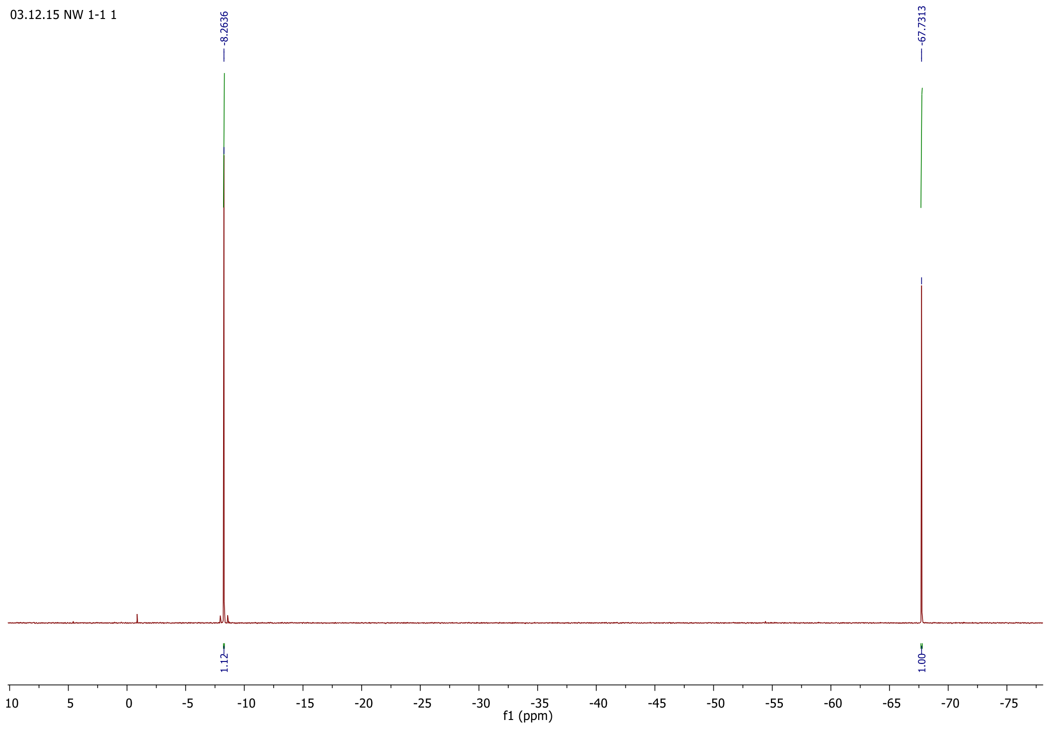


Figure S50: The $^{31}$ P NMR spectra of the compounds **12** and **13**

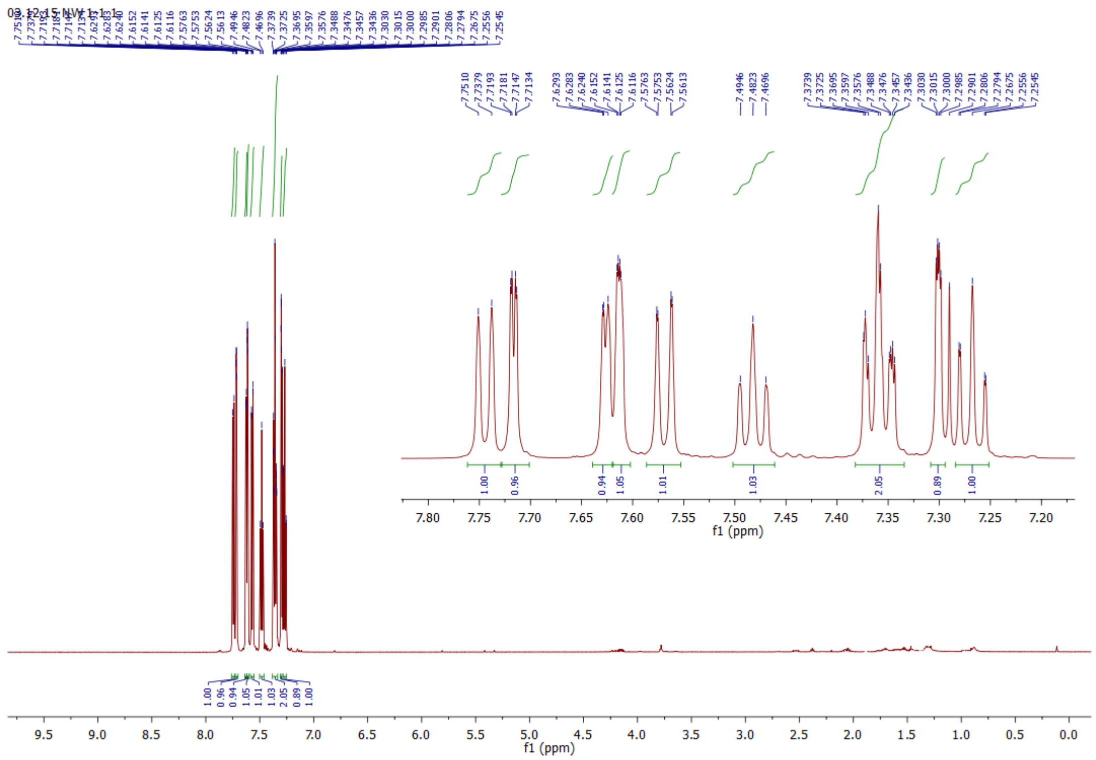


Figure S51: The [31] P NMR spectra of the compounds **12** and **13**

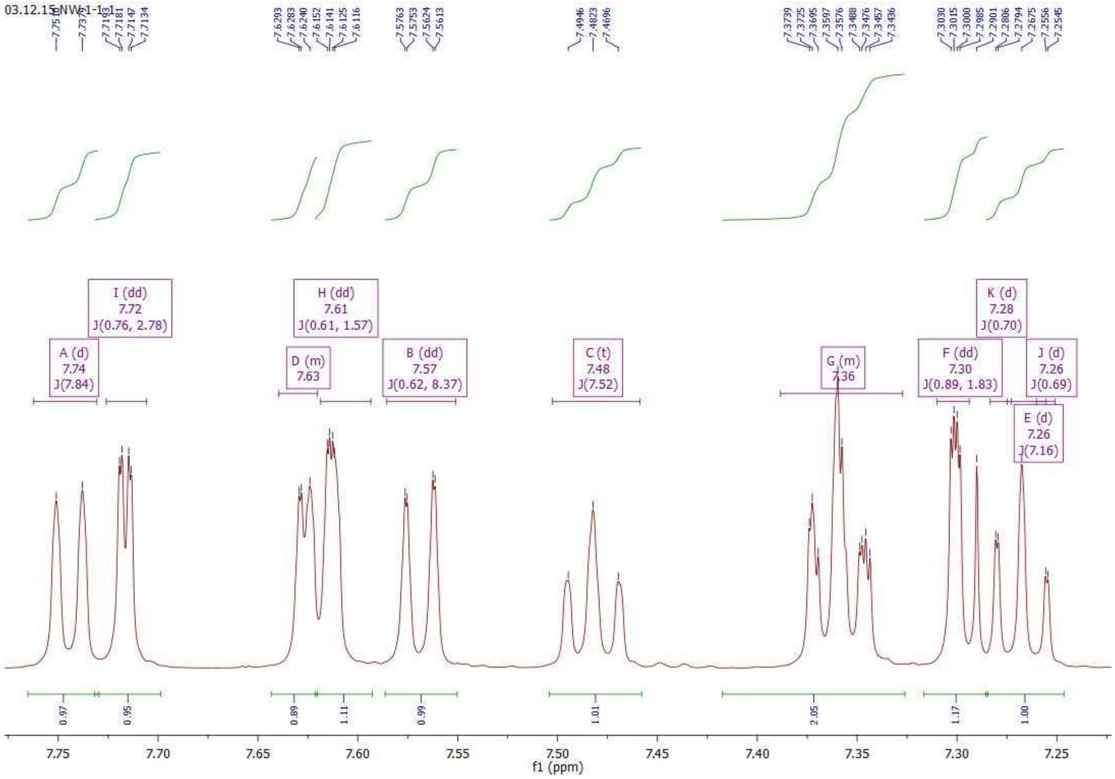


Figure S52: The [1] H NMR spectra of the compounds **12** and **13**

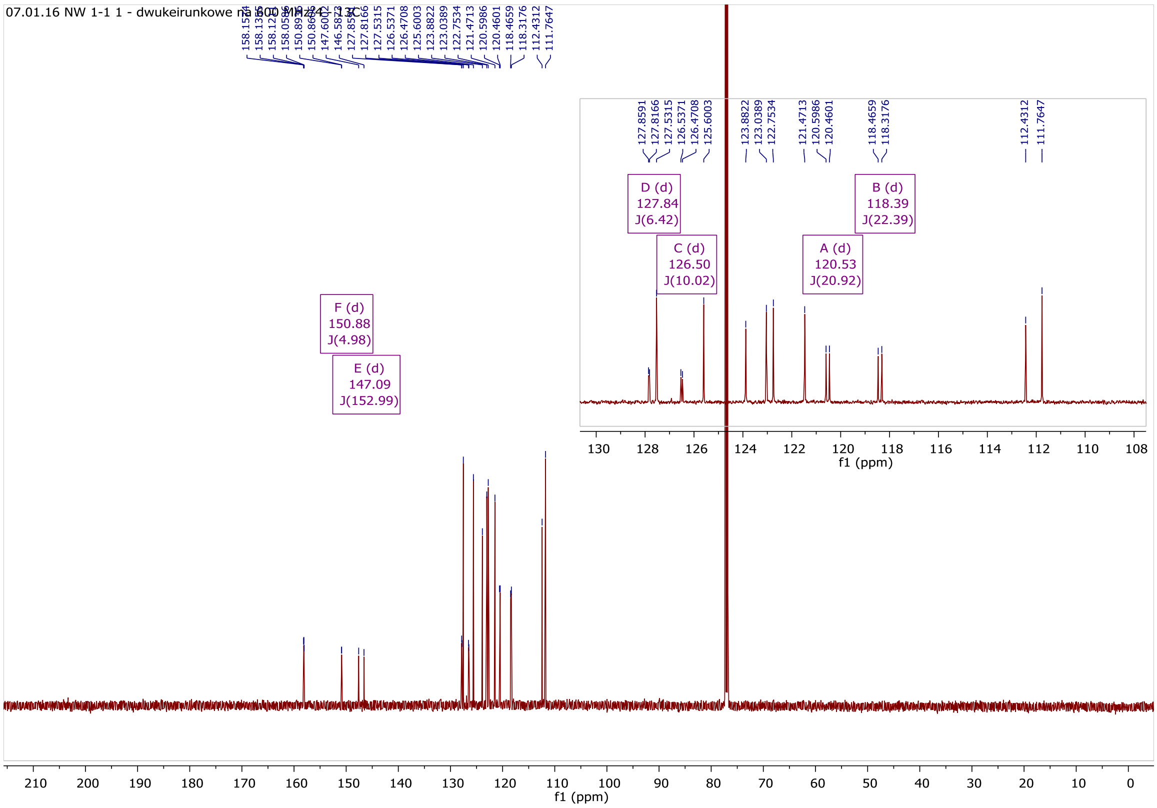


Figure S53: The [13] C NMR spectra of the compounds **12** and **13**

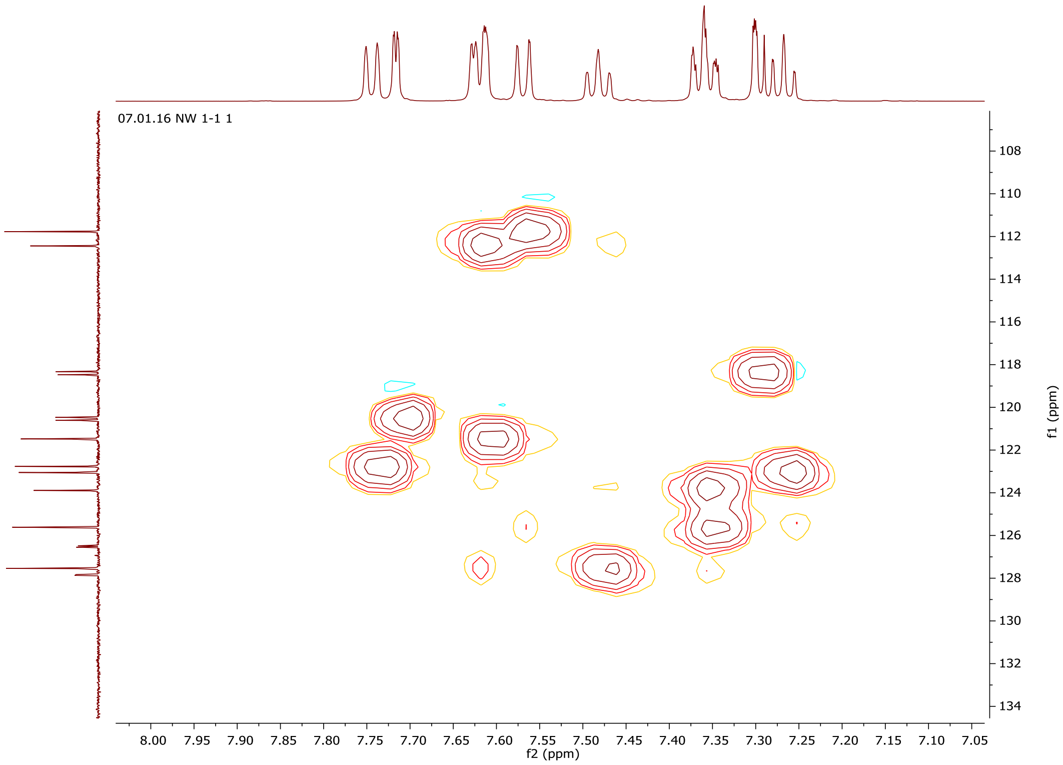


Figure S54: The [1] H - $^{13}$C NMR spectra of the compounds **12** and **13**

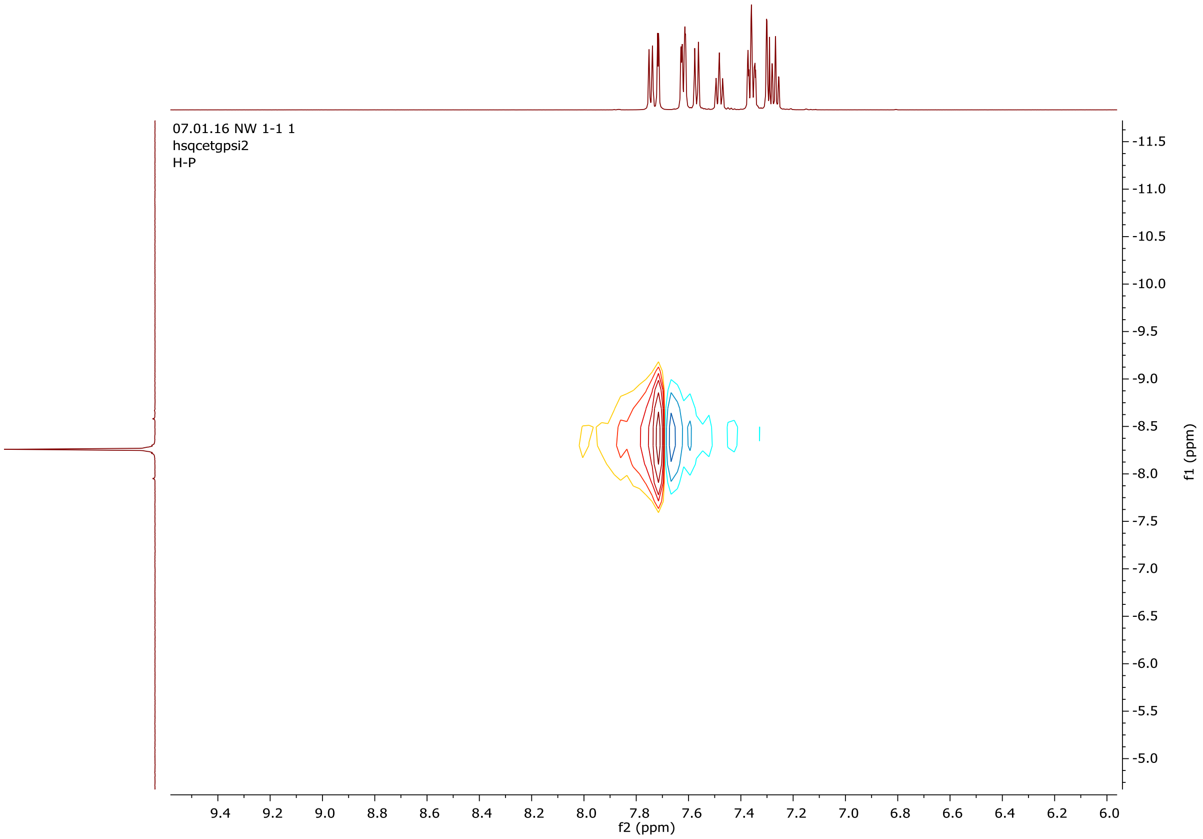


Figure S55: The ¹ H - ³¹P NMR spectra of the compounds **12** and **13**

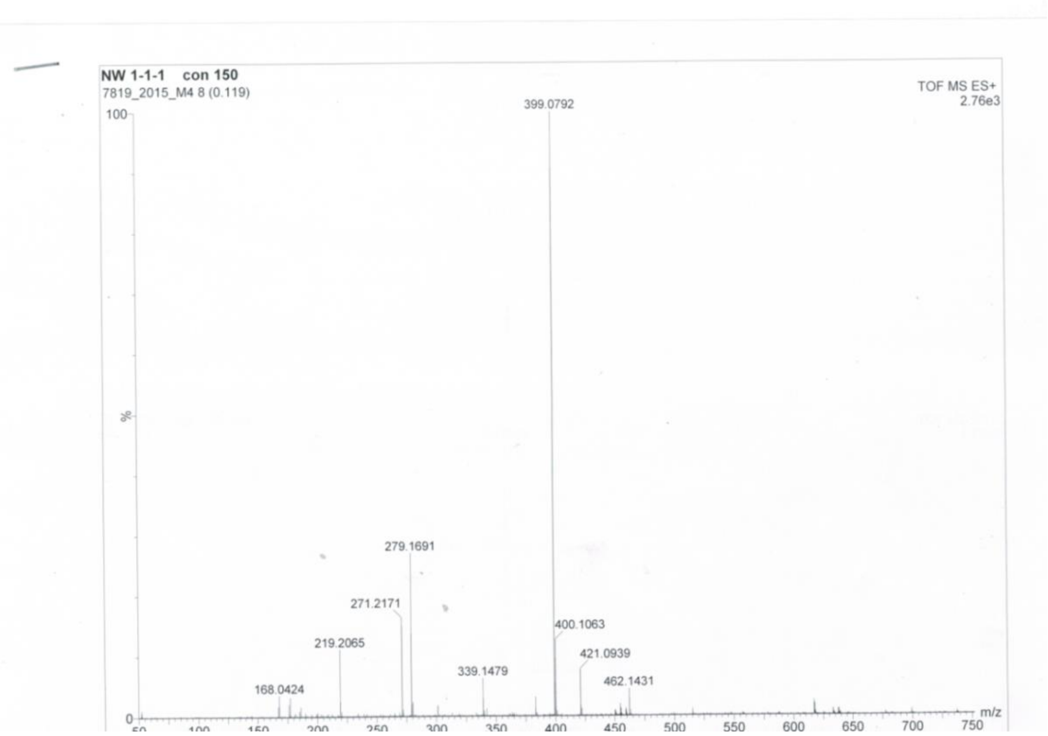


Figure S56: The HRMS NMR spectra of the compounds **12** and **13**

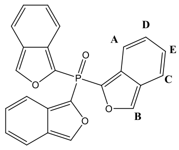


**13**

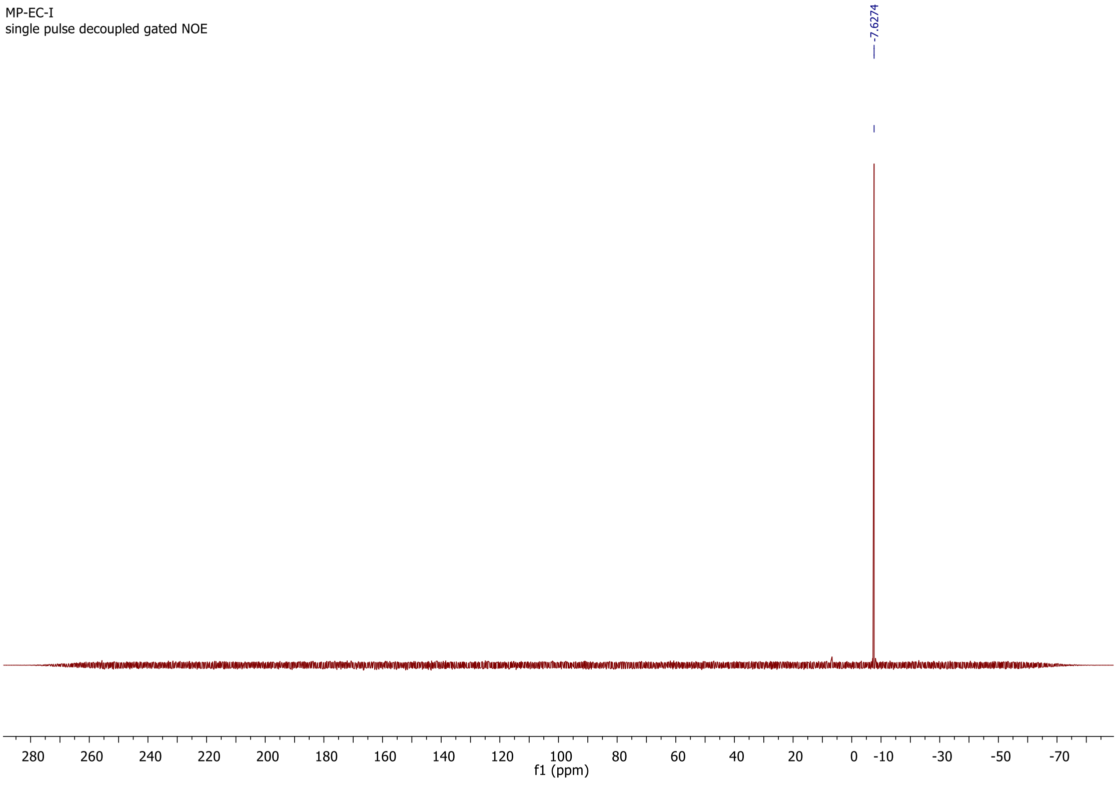


Figure S57: The $^{31}$ P NMR spectra of the compound **13**

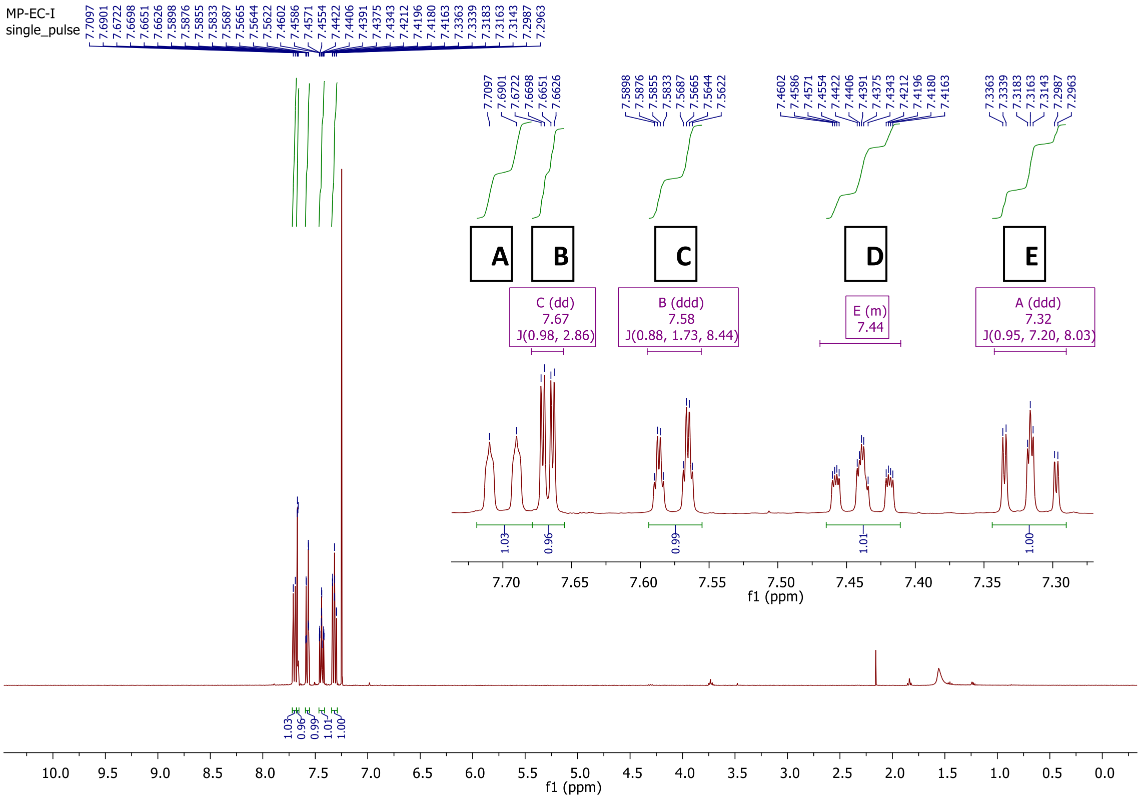

Figure S58: The [1] H NMR spectra of the compound **13**

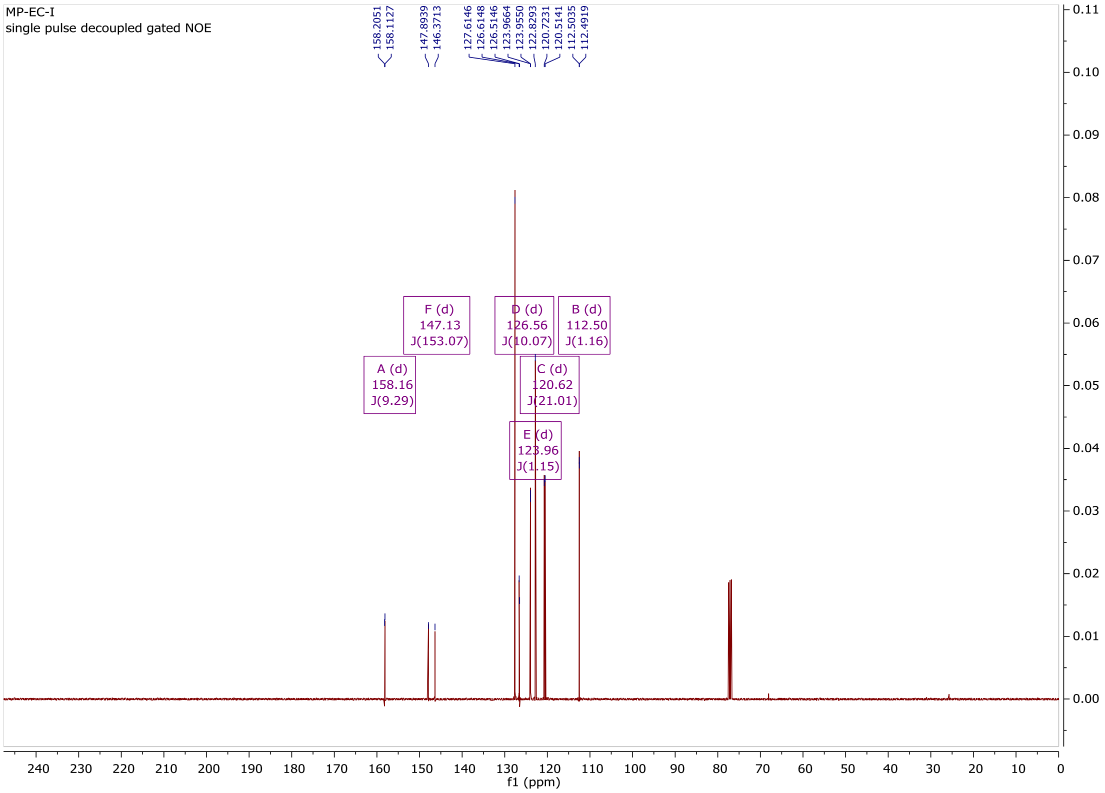

Figure S59: The [13] C NMR spectra of the compound **13**

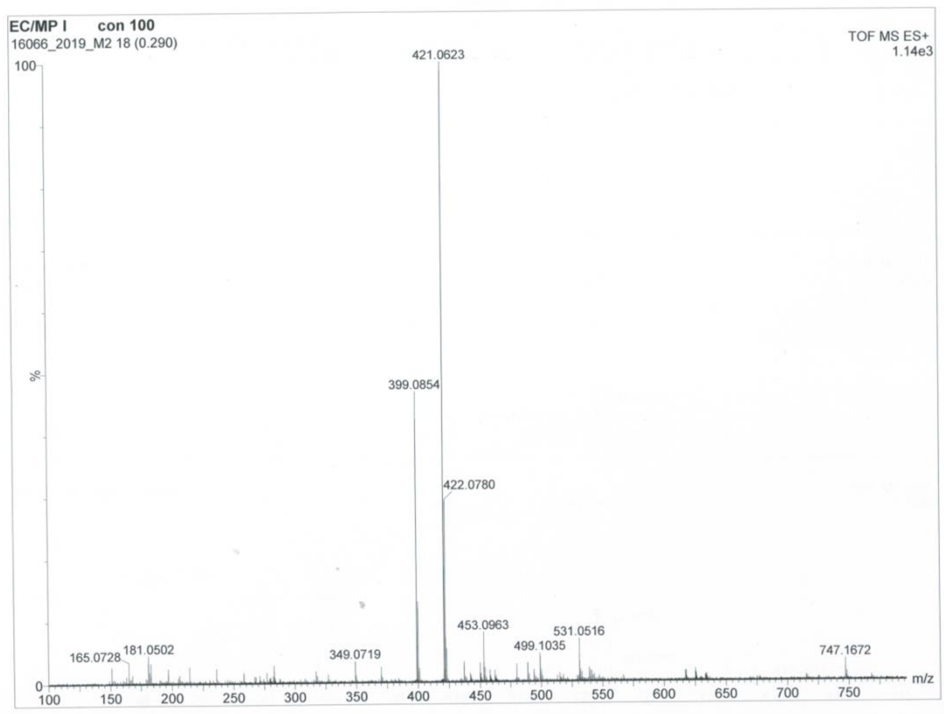


Figure S60: The HRMS spectra of the compound **13**

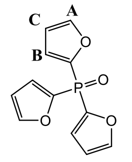


**15**

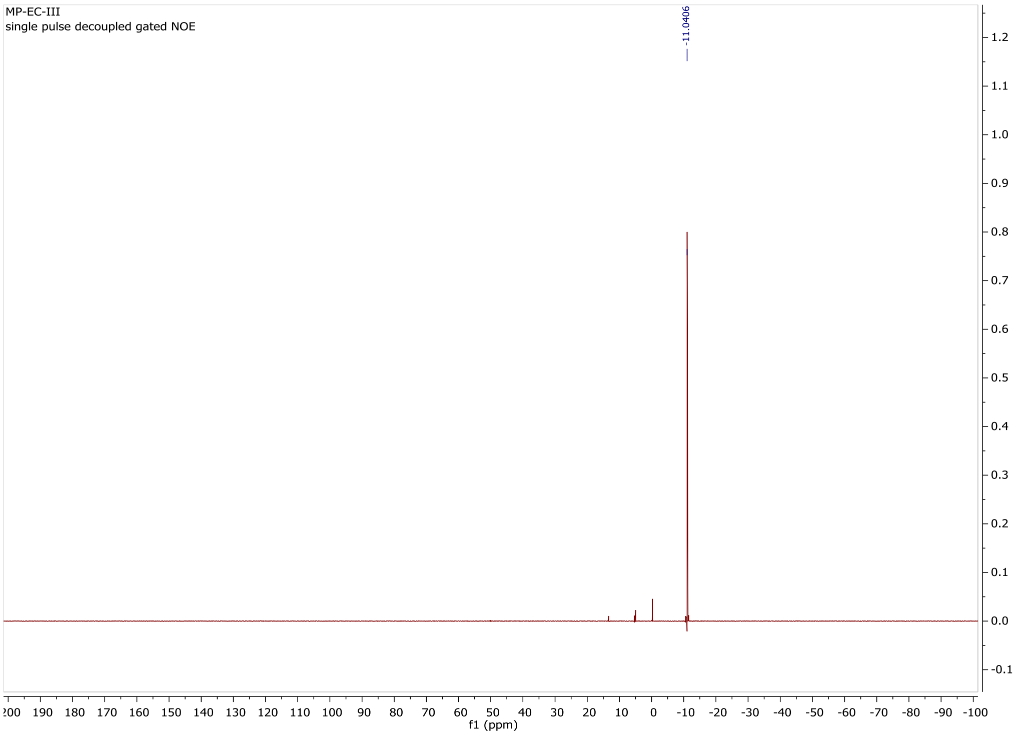


Figure S61: The $^{31}$ P NMR spectra of the compound **13**

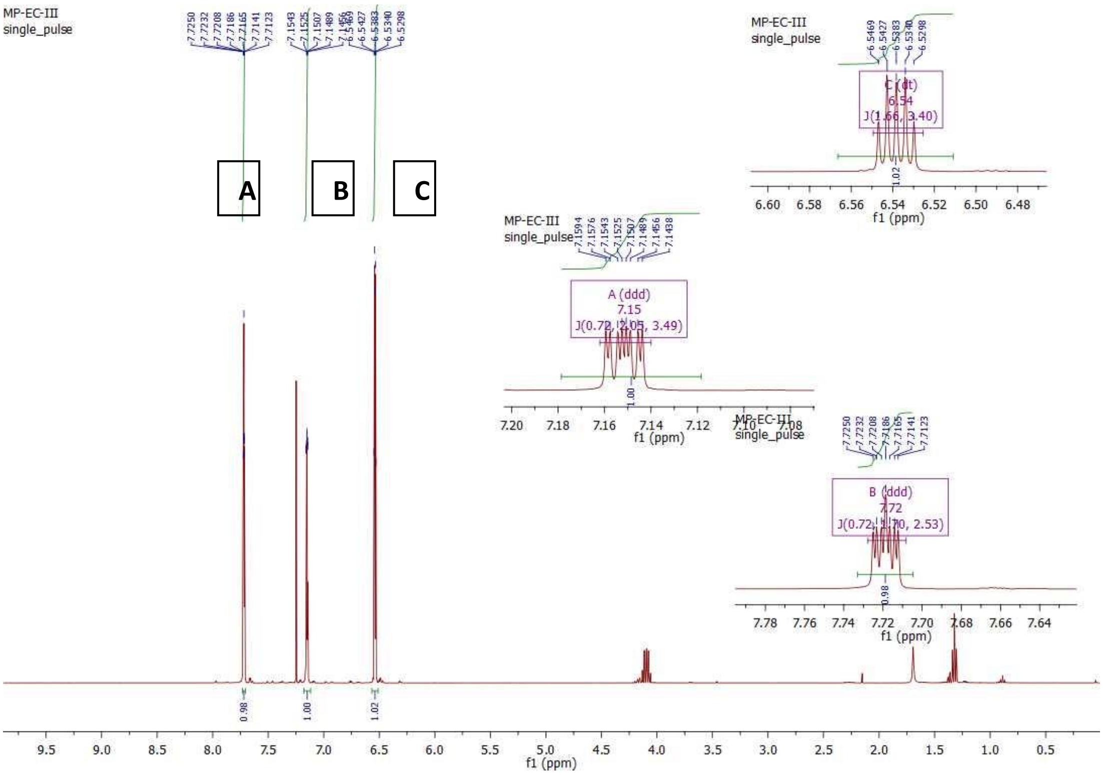


Figure S62: The [1] H NMR spectra of the compound **13**

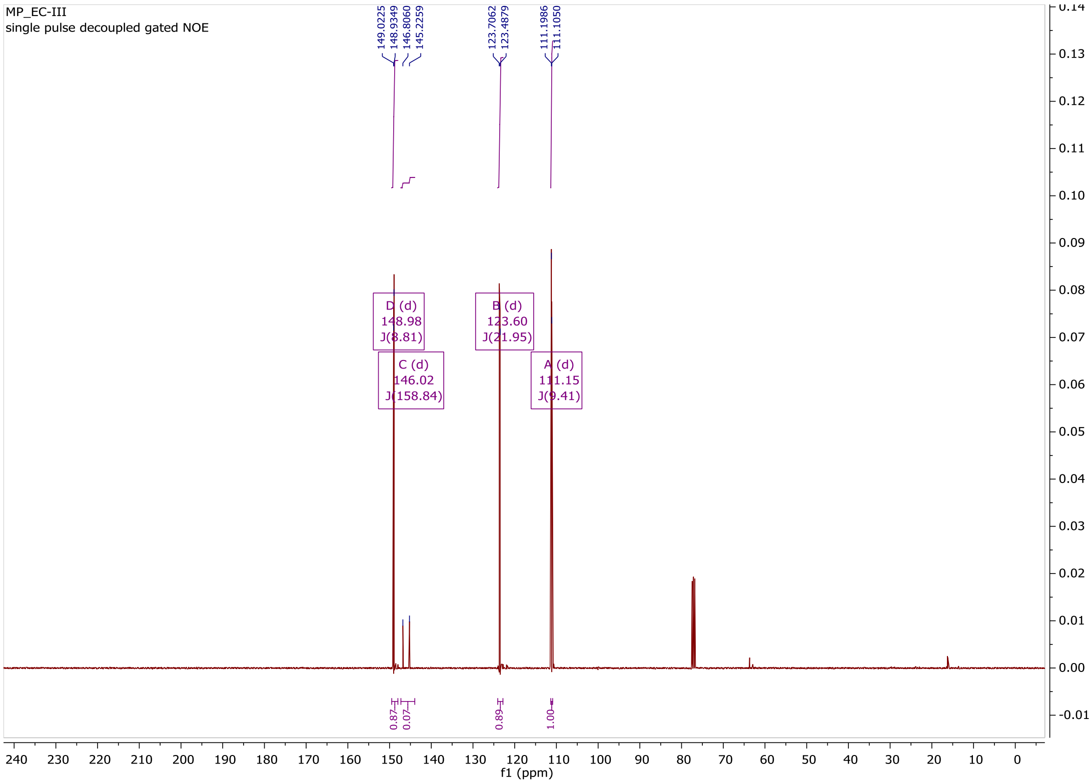


Figure S63: The [13] C NMR spectra of the compound **13**

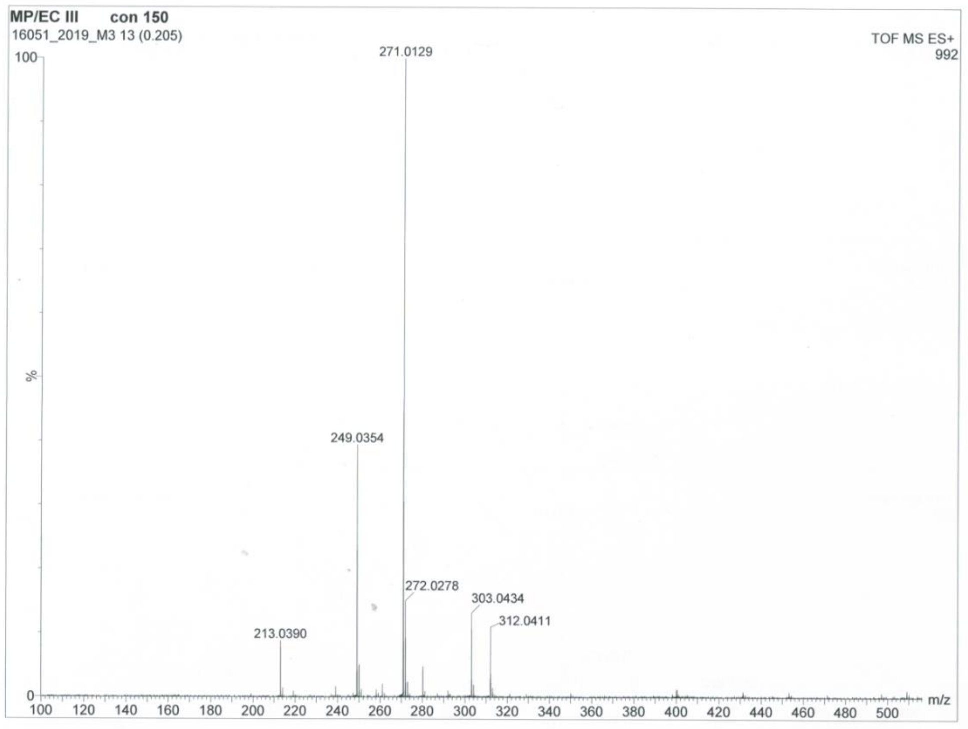


Figure S64: The HRMS spectra of the compound **13**

**14** 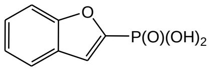


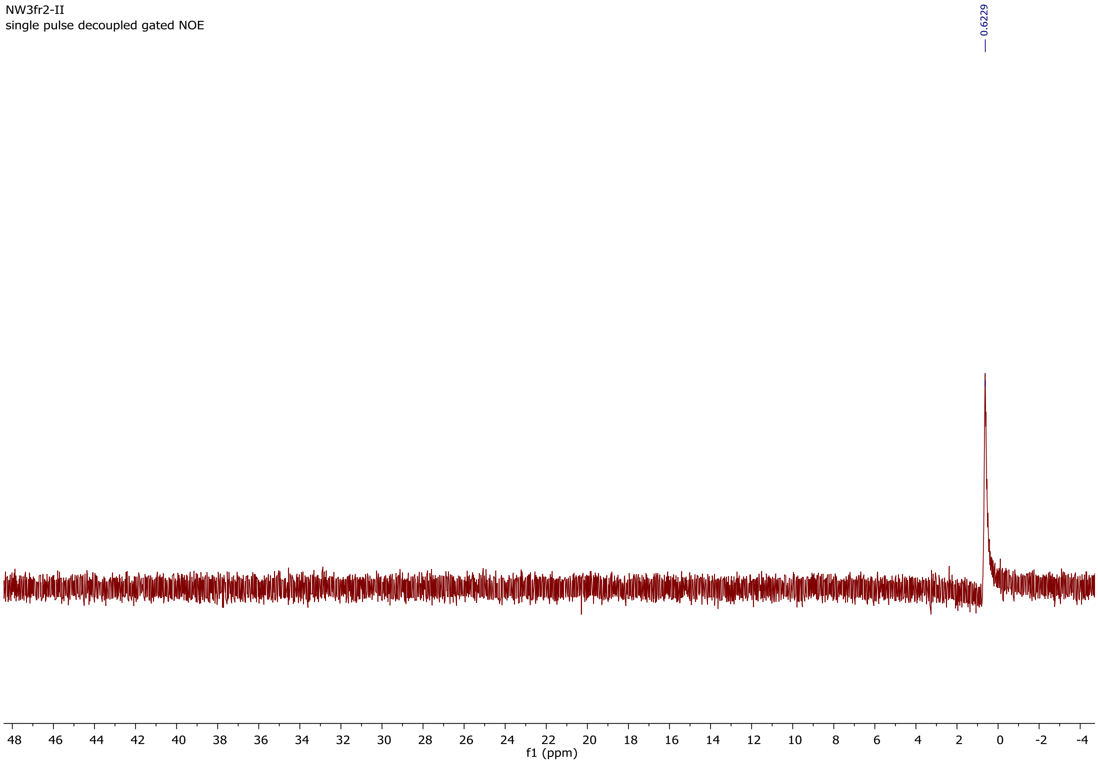


Figure S65: The $^{31}$ P NMR spectra of the compound **14**

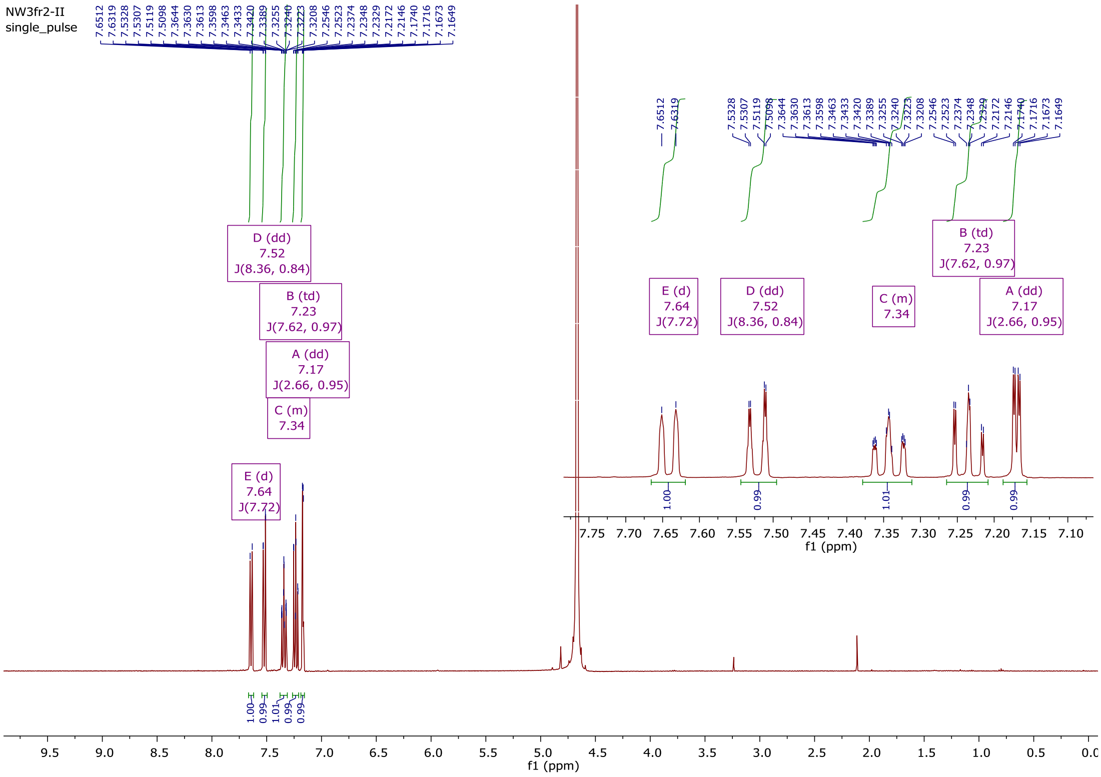


Figure S66: The [1] H NMR spectra of the compound **14**

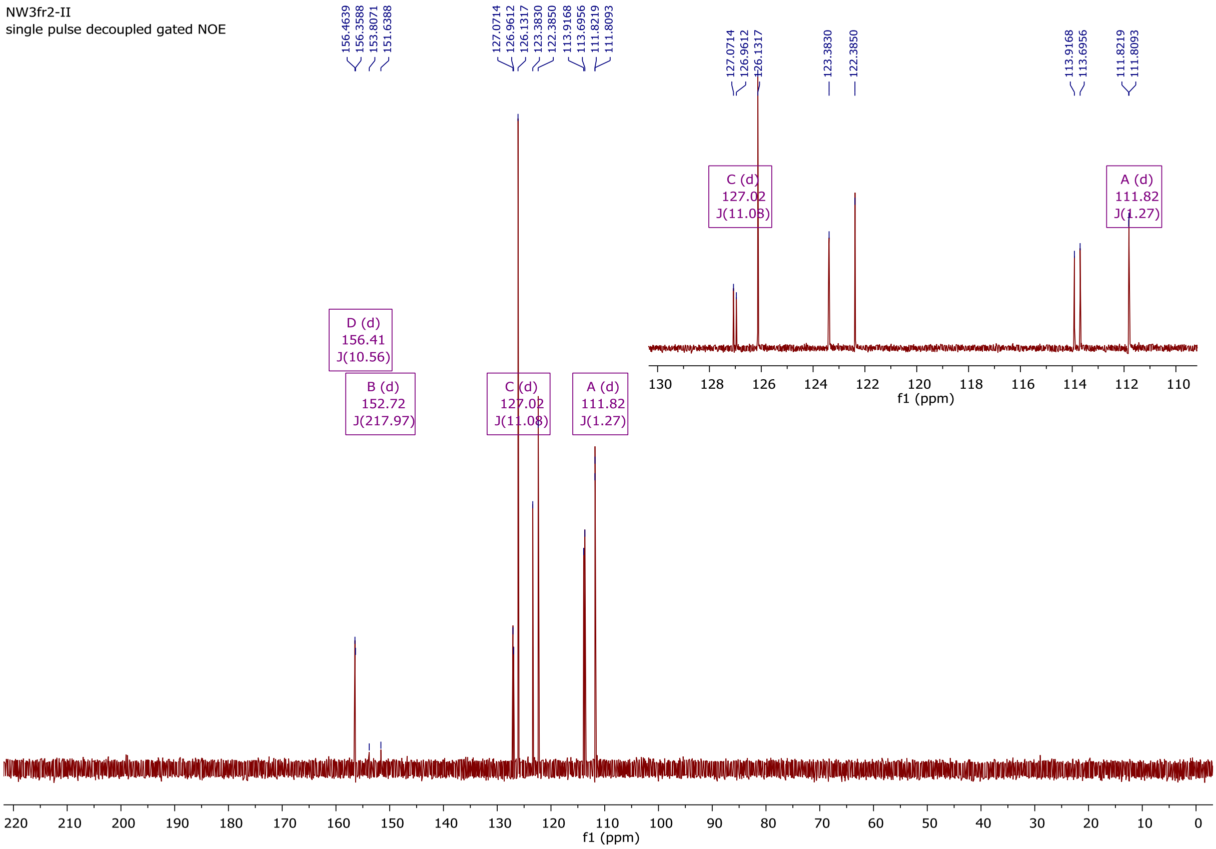


Figure S67: The [13] C NMR spectra of the compound **14**

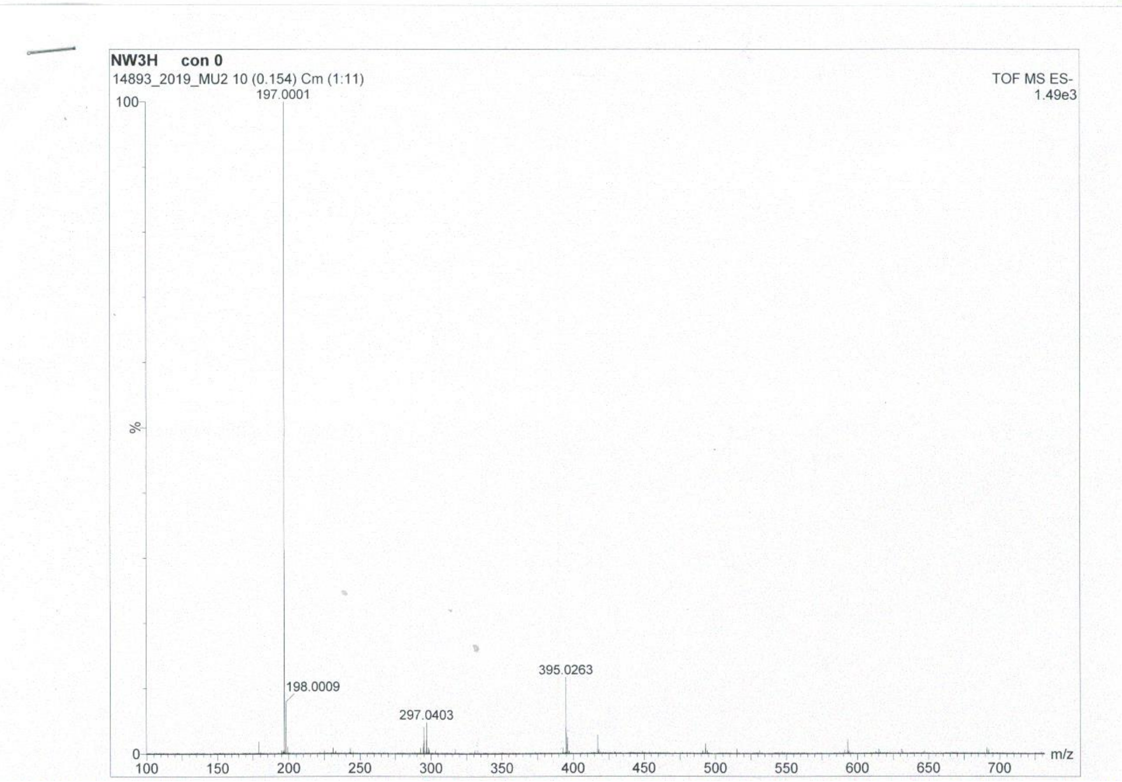


Figure S68: The HRMS spectra of the compound **14**

**16** 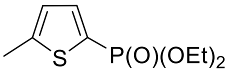


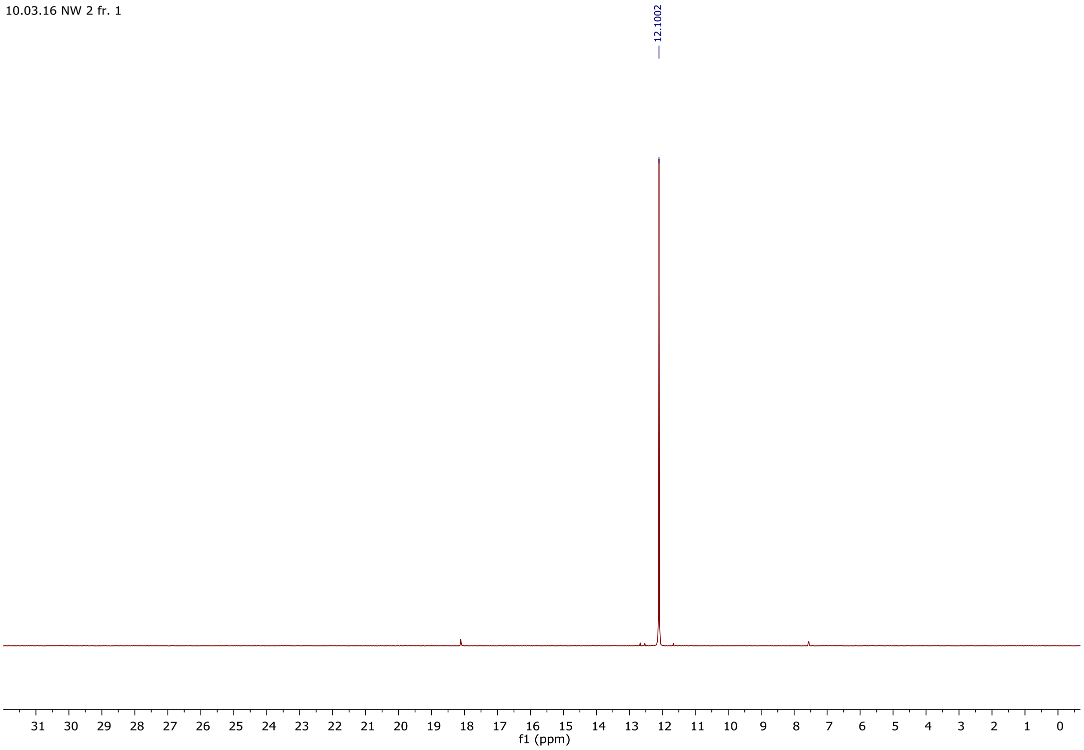


Figure S69: The $^{31}$ P NMR spectra of the compound **16**

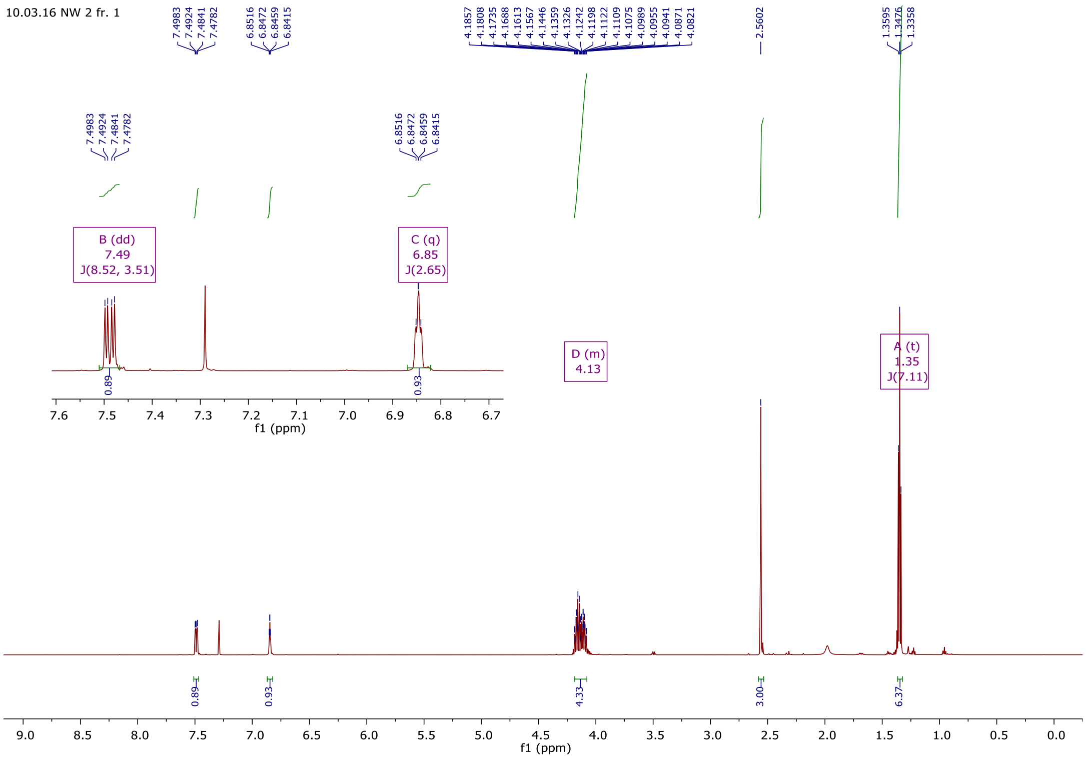


Figure S70: The [1] H NMR spectra of the compound **16**

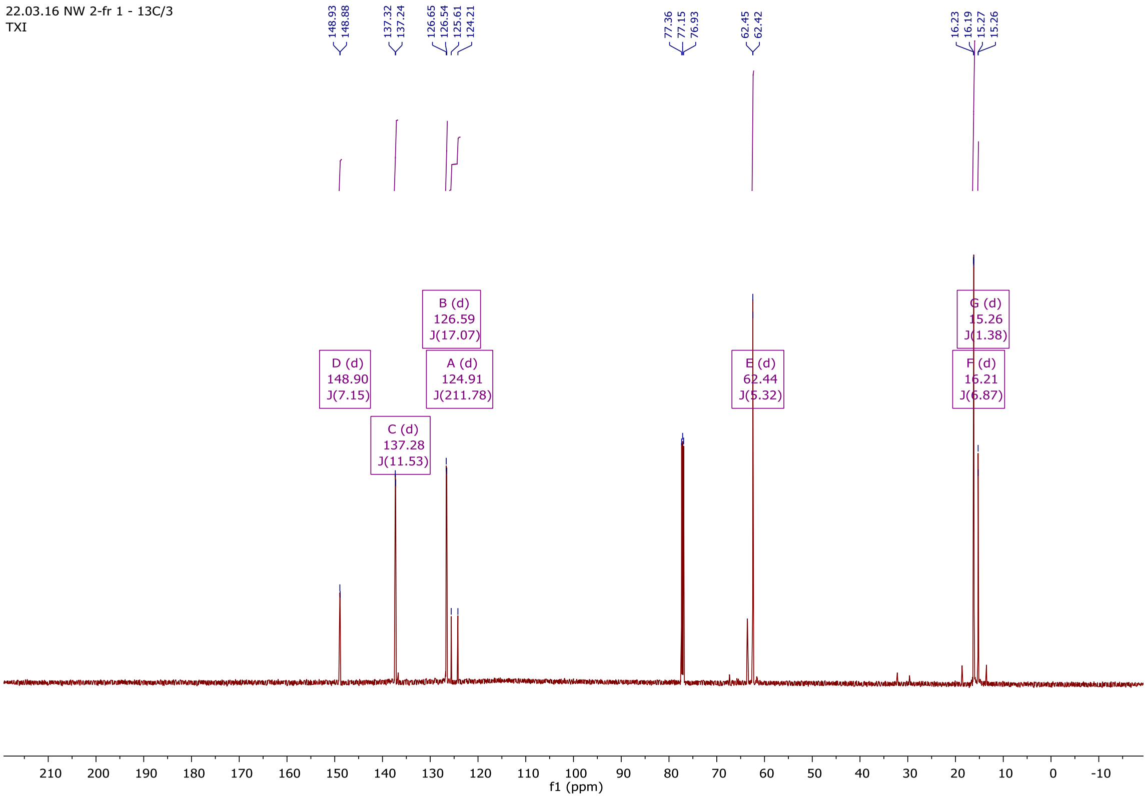


Figure S71: The [13] C NMR spectra of the compound **16**

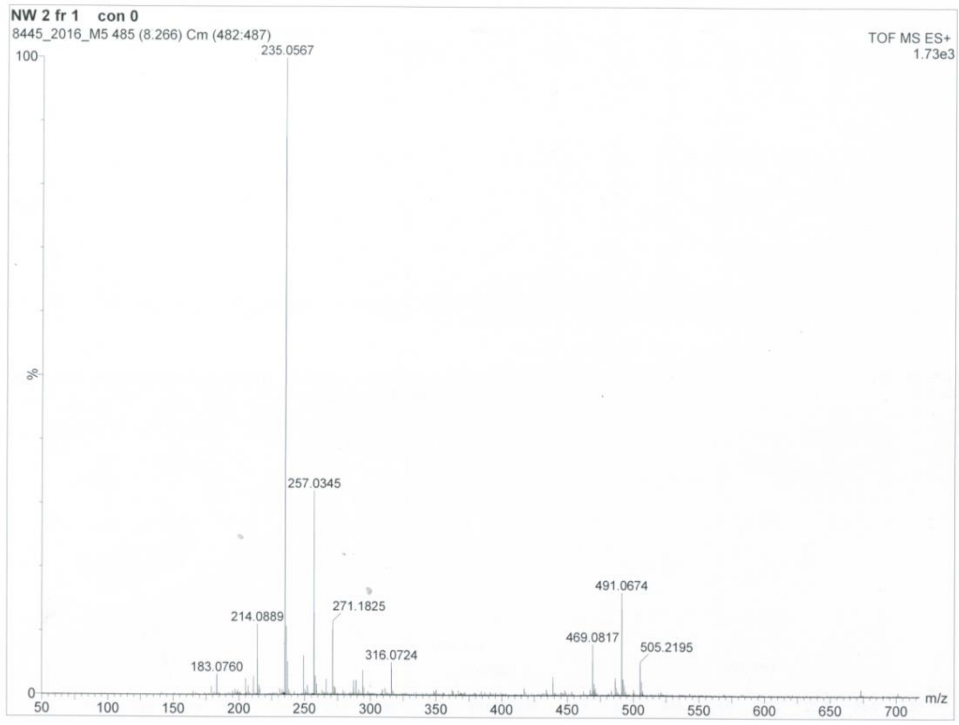


Figure S72: The HRMS spectra of the compound **16**

**17** 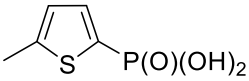


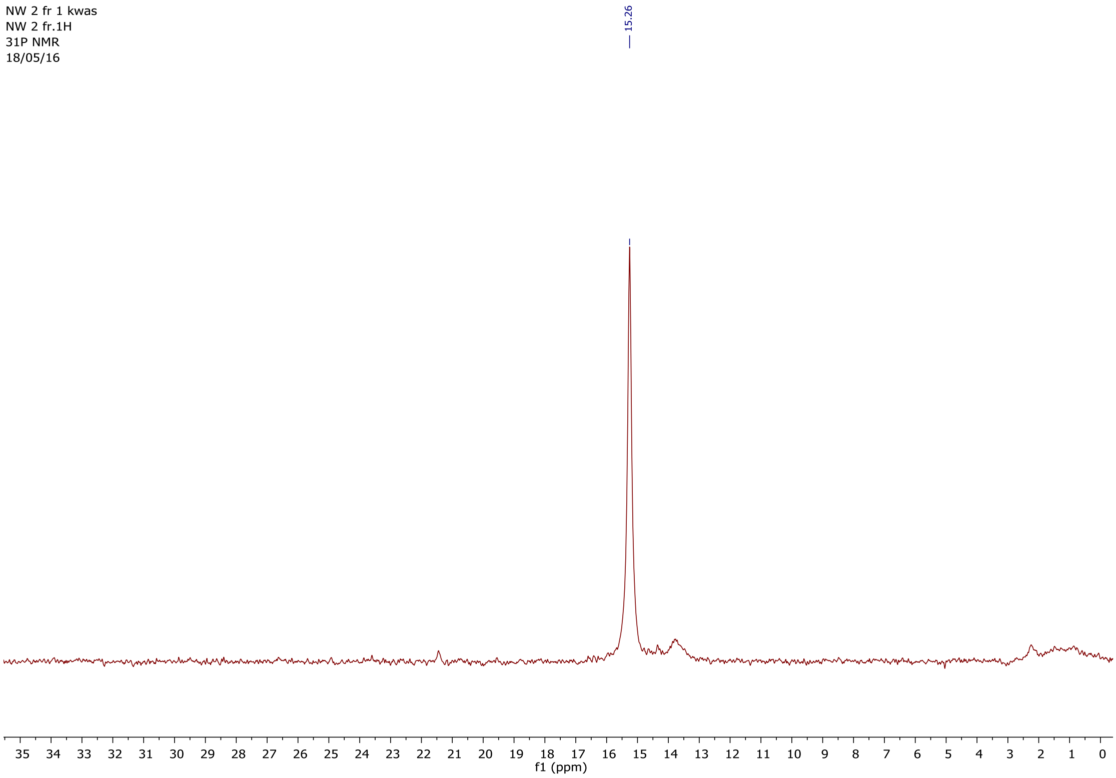


Figure S73: The $^{31}$P NMR spectra of the compound **17**

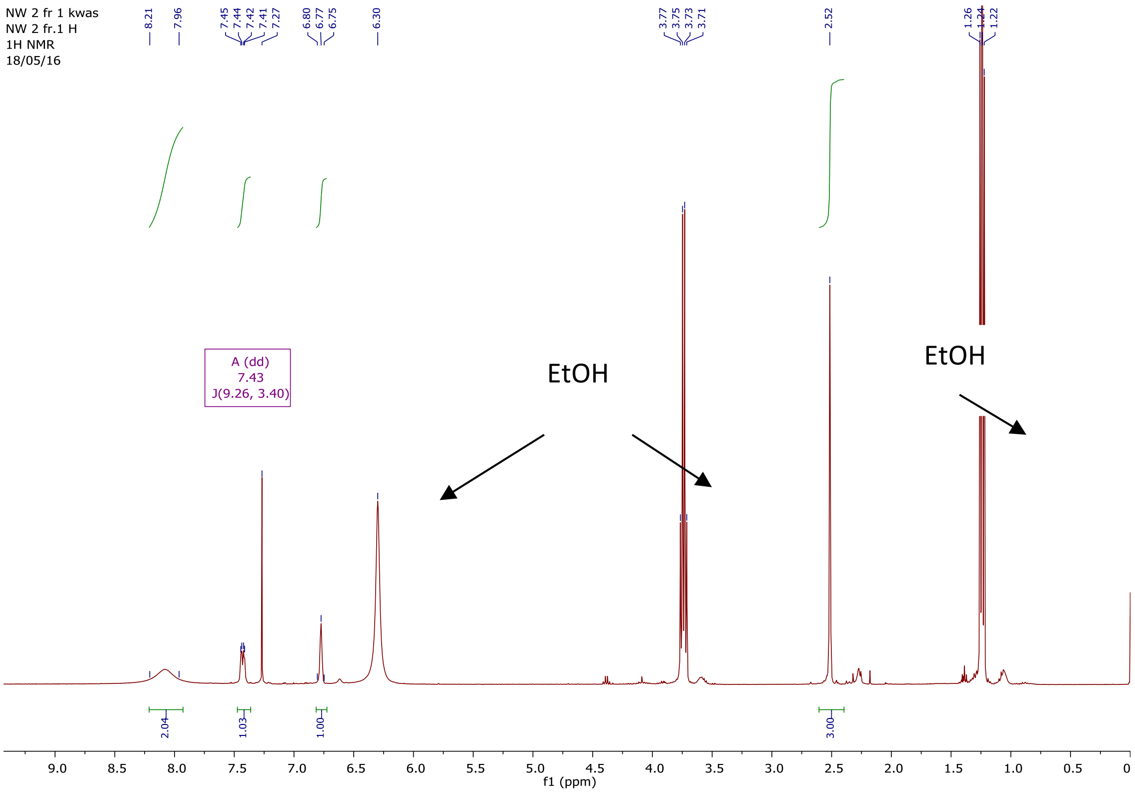


Figure S74: The [1] H NMR spectra of the compound **17**

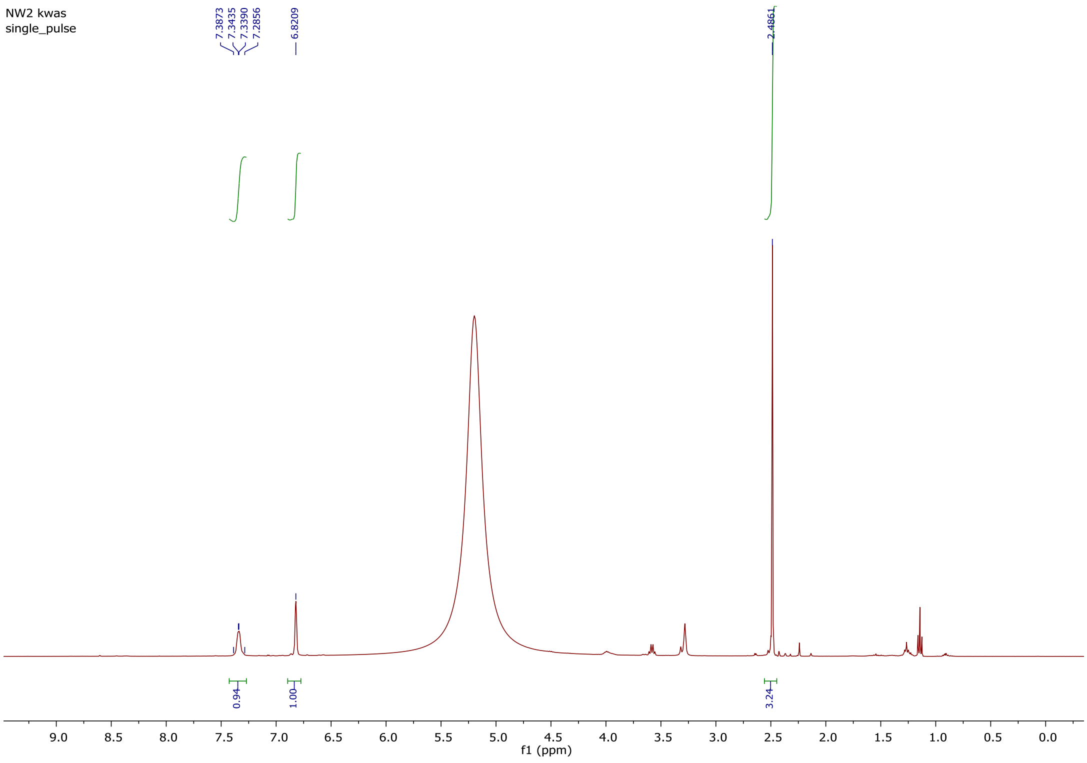


Figure S75: The [1] H NMR spectra of the compound **17**

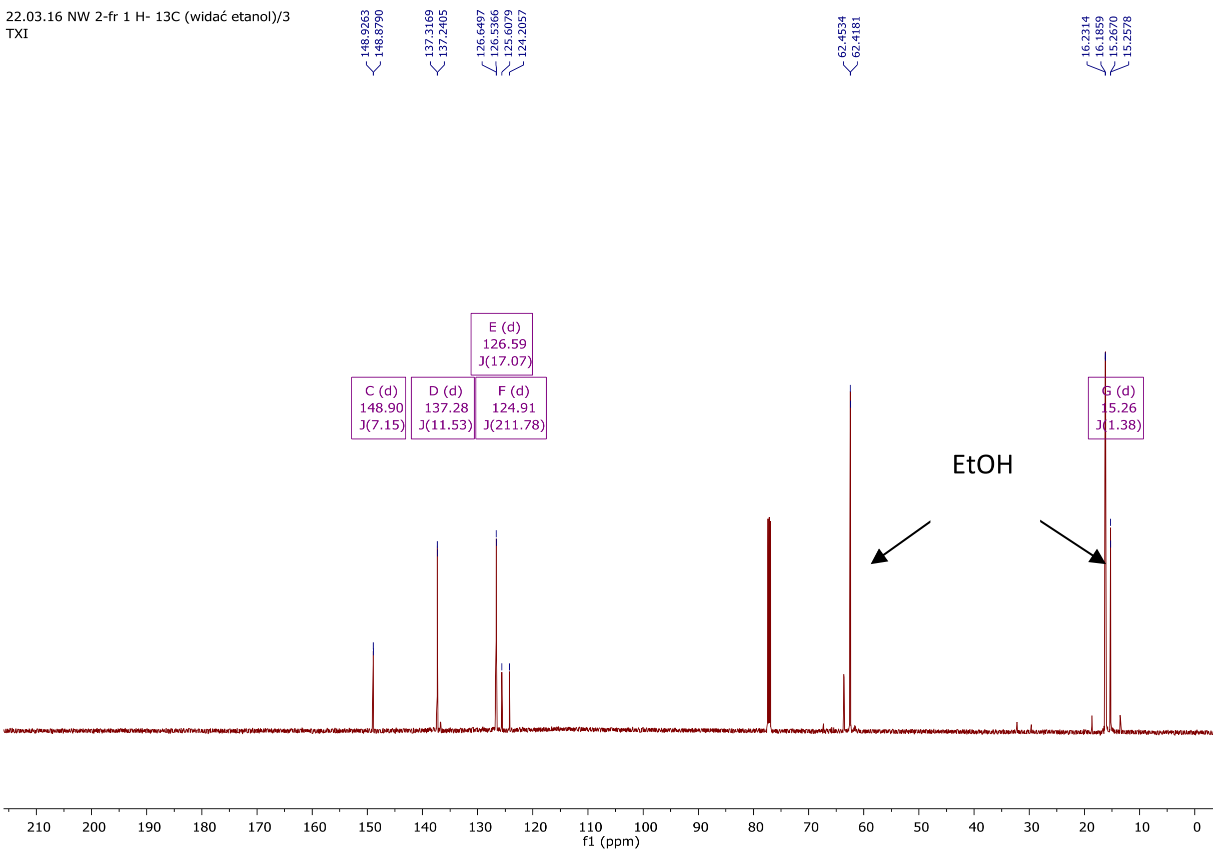


Figure S76: The [13] C NMR spectra of the compound **17**

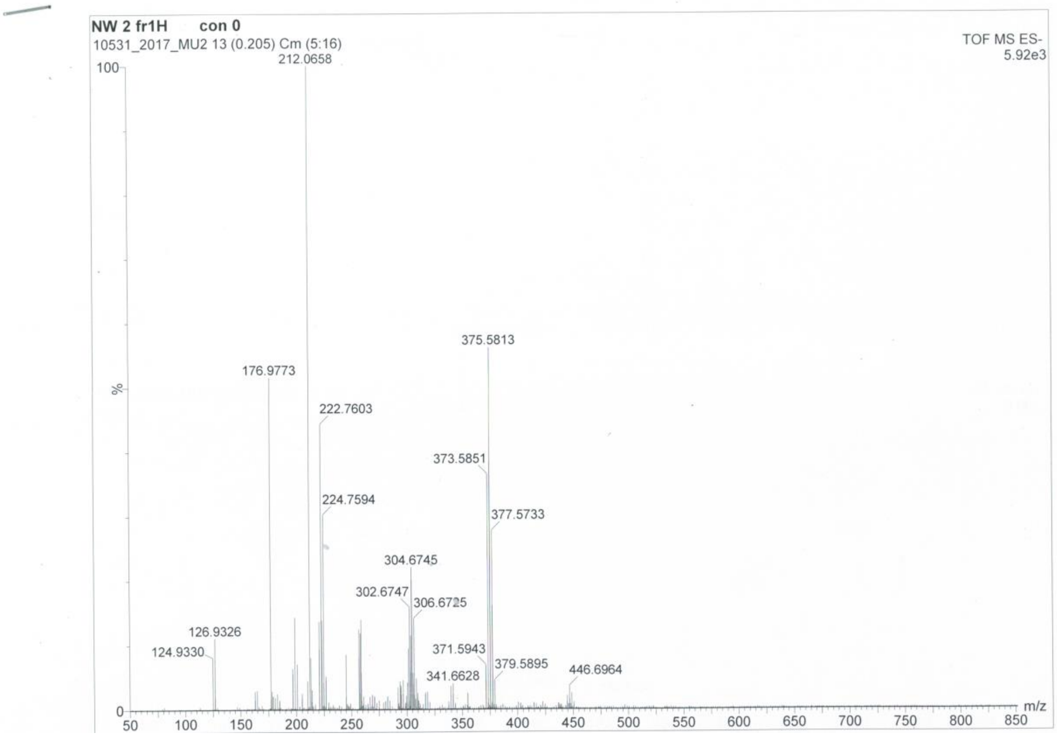


Figure S77: The CHRMS spectra of the compound **17**

**Table S1: Relevant crystallographic data for the molecule and the full geometrical information (Å, º)**

| | | | |
|---|---|---|---|
| **13** | | | |
| P1—O1 | 1.447 (3) | C10—C11 | 1.437 (2) |
| P1—C1 | 1.7930 (19) | C10—H10 | 0.9300 |
| P1—C17 | 1.7936 (19) | C11—C16 | 1.383 (2) |
| P1—C9 | 1.7947 (19) | C11—C12 | 1.400 (2) |
| O2—C8 | 1.382 (2) | C12—C13 | 1.375 (3) |
| O2—C1 | 1.404 (2) | C12—H12 | 0.9300 |
| O3—C16 | 1.381 (2) | C13—C14 | 1.386 (3) |
| O3—C9 | 1.394 (2) | C13—H13 | 0.9300 |
| O4—C24 | 1.380 (2) | C14—C15 | 1.370 (2) |
| O4—C17 | 1.384 (2) | C14—H14 | 0.9300 |
| O5—H5A | 1.08 (9) | C15—C16 | 1.382 (2) |
| O5—H5B | 1.09 (7) | C15—H15 | 0.9300 |
| C1—C2 | 1.338 (2) | C17—C18 | 1.336 (2) |
| C4—C5 | 1.374 (3) | C18—C19 | 1.430 (2) |
| C4—C3 | 1.406 (3) | C18—H18 | 0.9300 |
| C4—H4 | 0.9300 | C19—C24 | 1.377 (2) |
| C3—C8 | 1.382 (3) | C19—C20 | 1.403 (3) |
| C3—C2 | 1.437 (3) | C20—C21 | 1.388 (3) |
| C2—H2 | 0.9300 | C20—H20 | 0.9300 |
| C5—C6 | 1.388 (3) | C21—C22 | 1.393 (3) |
| C5—H5 | 0.9300 | C21—H21 | 0.9300 |
| C6—C7 | 1.382 (3) | C22—C23 | 1.362 (3) |
| C6—H6 | 0.9300 | C22—H22 | 0.9300 |
| C7—C8 | 1.382 (3) | C23—C24 | 1.381 (3) |
| C7—H7 | 0.9300 | C23—H23 | 0.9300 |
| C9—C10 | 1.342 (2) | | |
| O1—P1—C1 | 115.48 (13) | C16—C11—C10 | 105.64 (16) |
| O1—P1—C17 | 119.54 (13) | C12—C11—C10 | 136.13 (19) |
| C1—P1—C17 | 101.55 (9) | C13—C12—C11 | 118.49 (19) |
| O1—P1—C9 | 113.30 (13) | C13—C12—H12 | 120.8 |
| C1—P1—C9 | 103.49 (9) | C11—C12—H12 | 120.8 |
| C17—P1—C9 | 101.24 (9) | C12—C13—C14 | 121.31 (19) |
| C8—O2—C1 | 105.36 (15) | C12—C13—H13 | 119.3 |
| C16—O3—C9 | 105.62 (14) | C14—C13—H13 | 119.3 |
| C24—O4—C17 | 105.37 (14) | C15—C14—C13 | 121.65 (19) |
| H5A—O5—H5B | 113 (5) | C15—C14—H14 | 119.2 |
| C2—C1—O2 | 110.94 (17) | C13—C14—H14 | 119.2 |
| C2—C1—P1 | 129.31 (17) | C14—C15—C16 | 116.28 (19) |
| O2—C1—P1 | 119.73 (14) | C14—C15—H15 | 121.9 |
| C5—C4—C3 | 118.3 (2) | C16—C15—H15 | 121.9 |
| C5—C4—H4 | 120.9 | O3—C16—C15 | 125.58 (18) |
| C3—C4—H4 | 120.9 | O3—C16—C11 | 110.41 (16) |

| | | | |
|---|---|---|---|
| C8—C3—C4 | 118.5 (2) | C15—C16—C11 | 124.01 (18) |
| C8—C3—C2 | 105.92 (18) | C18—C17—O4 | 111.12 (17) |
| C4—C3—C2 | 135.5 (2) | C18—C17—P1 | 134.34 (16) |
| C1—C2—C3 | 107.37 (19) | O4—C17—P1 | 114.53 (13) |
| C1—C2—H2 | 126.3 | C17—C18—C19 | 107.51 (17) |
| C3—C2—H2 | 126.3 | C17—C18—H18 | 126.2 |
| C4—C5—C6 | 121.5 (2) | C19—C18—H18 | 126.2 |
| C4—C5—H5 | 119.2 | C24—C19—C20 | 118.01 (19) |
| C6—C5—H5 | 119.2 | C24—C19—C18 | 105.40 (17) |
| C7—C6—C5 | 121.6 (2) | C20—C19—C18 | 136.6 (2) |
| C7—C6—H6 | 119.2 | C21—C20—C19 | 118.1 (2) |
| C5—C6—H6 | 119.2 | C21—C20—H20 | 120.9 |
| C6—C7—C8 | 116.1 (2) | C19—C20—H20 | 120.9 |
| C6—C7—H7 | 122.0 | C20—C21—C22 | 120.7 (2) |
| C8—C7—H7 | 122.0 | C20—C21—H21 | 119.6 |
| C3—C8—O2 | 110.40 (18) | C22—C21—H21 | 119.6 |
| C3—C8—C7 | 124.1 (2) | C23—C22—C21 | 122.5 (2) |
| O2—C8—C7 | 125.5 (2) | C23—C22—H22 | 118.8 |
| C10—C9—O3 | 110.96 (16) | C21—C22—H22 | 118.8 |
| C10—C9—P1 | 130.01 (15) | C22—C23—C24 | 115.4 (2) |
| O3—C9—P1 | 118.88 (13) | C22—C23—H23 | 122.3 |
| C9—C10—C11 | 107.33 (17) | C24—C23—H23 | 122.3 |
| C9—C10—H10 | 126.3 | C19—C24—O4 | 110.58 (17) |
| C11—C10—H10 | 126.3 | C19—C24—C23 | 125.2 (2) |
| C16—C11—C12 | 118.22 (18) | O4—C24—C23 | 124.1 (2) |
| C8—O2—C1—C2 | 0.8 (2) | C10—C11—C12—C13 | −179.1 (2) |
| C8—O2—C1—P1 | 179.29 (13) | C11—C12—C13—C14 | −0.6 (3) |
| O1—P1—C1—C2 | 9.8 (2) | C12—C13—C14—C15 | −1.3 (3) |
| C17—P1—C1—C2 | −121.06 (19) | C13—C14—C15—C16 | 1.3 (3) |
| C9—P1—C1—C2 | 134.24 (19) | C9—O3—C16—C15 | −179.29 (17) |
| O1—P1—C1—O2 | −168.28 (17) | C9—O3—C16—C11 | 1.09 (19) |
| C17—P1—C1—O2 | 60.81 (16) | C14—C15—C16—O3 | −178.86 (17) |
| C9—P1—C1—O2 | −43.89 (16) | C14—C15—C16—C11 | 0.7 (3) |
| C5—C4—C3—C8 | 0.4 (3) | C12—C11—C16—O3 | 177.03 (16) |
| C5—C4—C3—C2 | 177.2 (2) | C10—C11—C16—O3 | −1.8 (2) |
| O2—C1—C2—C3 | −0.7 (2) | C12—C11—C16—C15 | −2.6 (3) |
| P1—C1—C2—C3 | −178.93 (14) | C10—C11—C16—C15 | 178.52 (17) |
| C8—C3—C2—C1 | 0.2 (2) | C24—O4—C17—C18 | 0.4 (2) |
| C4—C3—C2—C1 | −176.9 (2) | C24—O4—C17—P1 | 179.45 (12) |
| C3—C4—C5—C6 | −0.4 (3) | O1—P1—C17—C18 | −122.2 (2) |
| C4—C5—C6—C7 | 0.6 (4) | C1—P1—C17—C18 | 6.2 (2) |
| C5—C6—C7—C8 | −0.6 (3) | C9—P1—C17—C18 | 112.7 (2) |
| C4—C3—C8—O2 | 177.98 (16) | O1—P1—C17—O4 | 59.10 (19) |
| C2—C3—C8—O2 | 0.3 (2) | C1—P1—C17—O4 | −172.55 (13) |
| C4—C3—C8—C7 | −0.5 (3) | C9—P1—C17—O4 | −66.08 (14) |
| C2—C3—C8—C7 | −178.15 (18) | O4—C17—C18—C19 | 0.2 (2) |
| C1—O2—C8—C3 | −0.7 (2) | P1—C17—C18—C19 | −178.62 (15) |
| C1—O2—C8—C7 | 177.74 (19) | C17—C18—C19—C24 | −0.7 (2) |

| | | | |
|---|---|---|---|
| C6—C7—C8—C3 | 0.6 (3) | C17—C18—C19—C20 | 178.7 (2) |
| C6—C7—C8—O2 | −177.61 (18) | C24—C19—C20—C21 | 0.0 (3) |
| C16—O3—C9—C10 | 0.2 (2) | C18—C19—C20—C21 | −179.3 (2) |
| C16—O3—C9—P1 | −175.86 (12) | C19—C20—C21—C22 | 0.9 (3) |
| O1—P1—C9—C10 | 17.1 (2) | C20—C21—C22—C23 | −1.0 (3) |
| C1—P1—C9—C10 | −108.72 (19) | C21—C22—C23—C24 | 0.2 (3) |
| C17—P1—C9—C10 | 146.35 (18) | C20—C19—C24—O4 | −178.54 (16) |
| O1—P1—C9—O3 | −167.76 (16) | C18—C19—C24—O4 | 0.9 (2) |
| C1—P1—C9—O3 | 66.44 (15) | C20—C19—C24—C23 | −0.9 (3) |
| C17—P1—C9—O3 | −38.49 (15) | C18—C19—C24—C23 | 178.6 (2) |
| O3—C9—C10—C11 | −1.3 (2) | C17—O4—C24—C19 | −0.9 (2) |
| P1—C9—C10—C11 | 174.16 (14) | C17—O4—C24—C23 | −178.54 (19) |
| C9—C10—C11—C16 | 1.9 (2) | C22—C23—C24—C19 | 0.8 (3) |
| C9—C10—C11—C12 | −176.7 (2) | C22—C23—C24—O4 | 178.13 (19) |
| C16—C11—C12—C13 | 2.5 (3) | | |
| **15** | | | |
| P1—O1 | 1.4843 (16) | C3—H3 | 0.9300 |
| P1—C5 | 1.774 (2) | C4—H4 | 0.9300 |
| P1—C9 | 1.777 (2) | C5—C6 | 1.355 (3) |
| P1—C1 | 1.788 (2) | C6—C7 | 1.423 (4) |
| O2—C4 | 1.372 (3) | C6—H6 | 0.9300 |
| O2—C1 | 1.380 (2) | C7—C8 | 1.341 (4) |
| O3—C8 | 1.357 (3) | C7—H7 | 0.9300 |
| O3—C5 | 1.377 (3) | C8—H8 | 0.9300 |
| O4—C12 | 1.366 (3) | C9—C10 | 1.345 (4) |
| O4—C9 | 1.374 (3) | C10—C11 | 1.420 (4) |
| C1—C2 | 1.340 (3) | C10—H10 | 0.9300 |
| C2—C3 | 1.425 (3) | C11—C12 | 1.332 (4) |
| C2—H2 | 0.9300 | C11—H11 | 0.9300 |
| C3—C4 | 1.339 (3) | C12—H12 | 0.9300 |
| O1—P1—C5 | 115.84 (11) | O3—C5—P1 | 115.52 (15) |
| O1—P1—C9 | 111.50 (11) | C5—C6—C7 | 106.4 (2) |
| C5—P1—C9 | 104.79 (11) | C5—C6—H6 | 126.8 |
| O1—P1—C1 | 110.82 (10) | C7—C6—H6 | 126.8 |
| C5—P1—C1 | 105.84 (11) | C8—C7—C6 | 106.5 (2) |
| C9—P1—C1 | 107.50 (11) | C8—C7—H7 | 126.7 |
| C4—O2—C1 | 105.79 (16) | C6—C7—H7 | 126.7 |
| C8—O3—C5 | 106.22 (17) | C7—C8—O3 | 111.0 (2) |
| C12—O4—C9 | 106.0 (2) | C7—C8—H8 | 124.5 |
| C2—C1—O2 | 110.05 (18) | O3—C8—H8 | 124.5 |
| C2—C1—P1 | 132.50 (17) | C10—C9—O4 | 109.8 (2) |
| O2—C1—P1 | 117.42 (15) | C10—C9—P1 | 129.7 (2) |
| C1—C2—C3 | 107.03 (19) | O4—C9—P1 | 120.43 (18) |
| C1—C2—H2 | 126.5 | C9—C10—C11 | 106.7 (2) |
| C3—C2—H2 | 126.5 | C9—C10—H10 | 126.6 |
| C4—C3—C2 | 106.3 (2) | C11—C10—H10 | 126.6 |
| C4—C3—H3 | 126.8 | C12—C11—C10 | 106.6 (2) |

| | | | |
|---|---|---|---|
| C2—C3—H3 | 126.8 | C12—C11—H11 | 126.7 |
| C3—C4—O2 | 110.8 (2) | C10—C11—H11 | 126.7 |
| C3—C4—H4 | 124.6 | C11—C12—O4 | 110.8 (2) |
| O2—C4—H4 | 124.6 | C11—C12—H12 | 124.6 |
| C6—C5—O3 | 109.8 (2) | O4—C12—H12 | 124.6 |
| C6—C5—P1 | 134.64 (19) | | |
| C4—O2—C1—C2 | 0.3 (2) | C1—P1—C5—O3 | −62.84 (18) |
| C4—O2—C1—P1 | −178.03 (17) | O3—C5—C6—C7 | 0.3 (3) |
| O1—P1—C1—C2 | −7.3 (3) | P1—C5—C6—C7 | −179.7 (2) |
| C5—P1—C1—C2 | 119.0 (3) | C5—C6—C7—C8 | −0.3 (3) |
| C9—P1—C1—C2 | −129.4 (3) | C6—C7—C8—O3 | 0.1 (3) |
| O1—P1—C1—O2 | 170.58 (15) | C5—O3—C8—C7 | 0.1 (3) |
| C5—P1—C1—O2 | −63.07 (18) | C12—O4—C9—C10 | 1.0 (3) |
| C9—P1—C1—O2 | 48.5 (2) | C12—O4—C9—P1 | 178.51 (18) |
| O2—C1—C2—C3 | −0.4 (3) | O1—P1—C9—C10 | −9.1 (3) |
| P1—C1—C2—C3 | 177.62 (19) | C5—P1—C9—C10 | −135.2 (2) |
| C1—C2—C3—C4 | 0.3 (3) | C1—P1—C9—C10 | 112.5 (3) |
| C2—C3—C4—O2 | −0.1 (3) | O1—P1—C9—O4 | 173.96 (18) |
| C1—O2—C4—C3 | −0.1 (3) | C5—P1—C9—O4 | 47.9 (2) |
| C8—O3—C5—C6 | −0.3 (2) | C1—P1—C9—O4 | −64.4 (2) |
| C8—O3—C5—P1 | 179.76 (16) | O4—C9—C10—C11 | −1.0 (3) |
| O1—P1—C5—C6 | −119.5 (2) | P1—C9—C10—C11 | −178.2 (2) |
| C9—P1—C5—C6 | 3.8 (3) | C9—C10—C11—C12 | 0.6 (3) |
| C1—P1—C5—C6 | 117.2 (3) | C10—C11—C12—O4 | 0.0 (3) |
| O1—P1—C5—O3 | 60.38 (19) | C9—O4—C12—C11 | −0.6 (3) |
| C9—P1—C5—O3 | −176.31 (16) | | |

Table S2: Selected hydrogen-bond parameters

| *D*—H···*A* | *D*—H (Å) | H···*A* (Å) | *D*···*A* (Å) | *D*—H···*A* (°) |
|---|---|---|---|---|
| **13** | | | | |
| O5—H5A···O1$^{i}$ | 1.08 (9) | 1.91 (9) | 2.862 (3) | 145 (7) |
| C2—H2···O5 | 0.93 | 2.45 | 3.190 (3) | 136.5 |
| **15** | | | | |
| C2—H2···O2$^{ii}$ | 0.93 | 2.44 | 3.348 (3) | 165.6 |
| C4—H4···O1$^{iii}$ | 0.93 | 2.49 | 3.286 (3) | 143.5 |
| C7—H7···O3$^{iv}$ | 0.93 | 2.65 | 3.342 (3) | 132.1 |
| C6—H6···O1$^{v}$ | 0.93 | 2.47 | 3.373 (3) | 164.7 |
| C8—H8···O1$^{vi}$ | 0.93 | 2.39 | 3.264 (3) | 155.7 |

Symmetry code(s): (i) *y*+1/3, −*x*+*y*+2/3, −*z*+2/3; (ii) −*x*+1, *y*+1/2, −*z*+1/2; (iii) −*x*+1, *y*−1/2, −*z*+1/2; (iv) *x*+1/2, −*y*+1/2, −*z*+1; (v) −*x*+2, *y*−1/2, −*z*+1/2; (vi) −*x*+3/2, −*y*+1, *z*+1/2.
